# Discordance in orphan drug approvals between the U.S. Food and Drug Administration and the European Medicines Agency: A retrospective observational analysis

**DOI:** 10.1371/journal.pmed.1004861

**Published:** 2026-07-06

**Authors:** Jin Ding, Michael M. Hopkins, Paul A. Martin

**Affiliations:** 1 iHuman, School of Sociological Studies, Politics and International Relations, The University of Sheffield, Sheffield, United Kingdom; 2 Department of Management, Business School, University of Exeter, Exeter, United Kingdom; 3 Science Policy Research Unit, Business School, University of Sussex, Brighton, United Kingdom; 4 Centre for Law, Medicine and the Life Sciences, Faculty of Law, University of Cambridge, Cambridge, United Kingdom; Ministry of Health: Gobierno de Chile Ministerio de Salud, CHILE

## Abstract

**Background:**

The United States (US) and European Union (EU) have long-established orphan drug regulations to incentivise the development of medicines for rare diseases. While the numbers of orphan approvals have risen rapidly, there is increasing discordance in regulatory outcomes between the US and EU. This discordance primarily stems from two sets of cases: US Food and Drug Administration (FDA) orphan approvals not authorised by the European Medicines Agency (EMA), and FDA orphan approvals with EMA authorisation but without orphan designation. We examined factors associated with these two sets of cases to understand the growing gap in orphan approvals between the US and EU.

**Methods and findings:**

We collected data on FDA orphan drug approvals between 2011 and 2023 from the FDA Orphan Drug Designations and Approvals Database and their corresponding EMA regulatory status from EMA medicines database. We used descriptive statistical analysis to examine trends and identify discordance in outcomes between agencies. Univariable logistic regression assessed pre-specified factors associated with discordance, including therapeutic area (cancer/non-cancer), company size (large/medium/small), company headquarters location (US/EU/others) and approval period (2011–2016/2017–2023). The main methodological limitations are that the study identified associations but does not establish causality, with unmeasured factors potentially contributing to the observed discordance.

Of 814 FDA orphan approvals, only 29% received corresponding EMA marketing authorisation with orphan designation. A further 38% were authorised by the EMA but without orphan status, while the remaining 33% were not authorised by the EMA. Compared with the 2011–2016 period, cases in the 2017–2023 period were associated with lower odds (OR 0.66 (95% CI [0.48,0.92]; *p* = 0.013)) of EMA marketing authorisation. Compared with cancer approvals, non-cancer approvals were associated with lower odds (OR 0.53 (95% CI [0.38, 0.75]; *p* < 0.001)) of having EMA marketing authorisation, but when authorised, were associated with higher odds (OR 2.36 (95% CI [1.50, 3.70]; *p* < 0.001)) of receiving orphan designation. Compared with large companies, orphan approvals from small and medium-sized companies were associated with lower odds (OR 0.45 (95% CI [0.28, 0.74]; *p* = 0.001) and (OR 0.29 (95% CI [0.20, 0.43]; *p* < 0.001)) of EMA marketing authorisation, but among authorised products they were associated with higher odds (OR 2.95 (95% CI [1.75, 4.99]; *p* < 0.001) and (OR 2.14 (95% CI [1.17, 3.90]; *p* = 0.014)) of orphan-designated marketing authorisation, respectively. EU companies were associated with higher odds (OR 1.69 (95% CI [1.17–2.43]; *p* = 0.005)) of receiving EMA orphan approvals compared with US companies.

**Conclusions:**

Between 2011 and 2023, regulatory outcomes for orphan drug approvals increasingly diverged between the FDA and the EMA, particularly for cancer indications and approvals sponsored by small US sponsors. Among FDA orphan drugs authorised by the EMA, many were not designated as orphan products because of regulatory differences, particularly regarding requirements around significant benefit and biomarker-defined sub-populations in oncology. FDA-approved orphan drugs that lack EU marketing authorisation may be withheld by companies not because of regulatory barriers but due to insufficient commercial incentives to launch in Europe, resulting in fewer treatment options for European rare disease patients. Our findings suggest that orphan incentives are not the primary driver of commercial EU-launch decisions and therefore incremental changes to regulatory incentives seem unlikely to improve the access to orphan drugs for EU patients.

## Author summary

**Why was this study done?:**

- Although both the US Food and Drug Administration (FDA) and the European Medicines Agency (EMA) have approved increasing numbers of orphan drugs over time, there is a growing discordance in outcomes for the same indication between the two regions that these regulators serve.
- It remains poorly understood whether this gap is associated with product-level characteristics or company decisions.

**What did the researchers do and find?:**

- We performed an analysis of discordance in orphan approvals at the level of indication approval between the FDA and EMA, across all therapeutic areas and by commercial sponsor size and headquarters location.
- Only 29% of FDA-approved orphan drug approvals were similarly designated and authorised by the EMA. In contrast, a larger proportion of drugs were either authorised without orphan status (38%) or lacked marketing authorisation by the EMA (33%).
- Where FDA orphan approvals did not have a corresponding EMA marketing authorisation for the relevant indication this correlates with sponsoring company characteristics, with small, US-based companies in particular not seeking market authorisation in Europe, potentially for commercial reasons.

**What do these findings mean?:**

- These findings may inform policymakers seeking to stimulate innovation and improve patient access to important treatments.
- The findings question the likely effectiveness of policies relying on orphan market exclusivity to influence company behaviour.
- A limitation of this study is that factors beyond regulatory approval, such as therapeutic value, pricing and reimbursement policies that were not studied may also be associated with the observed discordance.

## Abstract 3

In a retrospective analysis, Jin Ding and colleagues explore the discrepancy in regulatory authorization of orphan drugs by U.S. and European regulatory agencies.

## Introduction

Orphan drugs have become a major focus for medicines developers and now account for half of all new drugs approved each year by the United States (US) Food and Drug Administration (FDA) [1]. Yet substantial differences have emerged in the availability of these drugs across jurisdictions. This raises important policy questions about patient access to therapy, particularly in light of recent proposals for regulatory change in the European Union (EU) [2]. The US Orphan Drug Act (ODA) was enacted in 1983 to encourage the availability of drugs for rare diseases by incentivising the development of medicines for markets that might otherwise be commercially unattractive. Incentives provided by the ODA include a 7-year period of market exclusivity, tax credits, research grants, and regulatory assistance [3]. Incentives only apply to drugs receiving an orphan drug designation from the FDA, with such designations only available for medicines with patient populations of less than 200,000 in the US. The ODA has been credited with stimulating an increased level of innovation [4,5]. In assessing such activity, the term ‘orphan approvals’ refers to FDA drug approvals or EMA marketing authorisations for orphan-designated indications [4].

The EU adopted its own orphan legislation in 2000, however, the rate of orphan approvals by the European Medicines Agency (EMA) is much lower compared to the FDA [6]. The factors associated with the discordance between the FDA and the EMA in terms of orphan approvals remain poorly understood. This study uses a stepwise analysis to explore the association between pre-specified variables and the apparent trends.

This study places emphasis on examining approved drug indications to investigate the evolving differences in rates of approval between the FDA and the EMA. We take this perspective because international comparative analysis of orphan drug approvals must account for the tendency of drugs to be approved for different numbers of indications at both the FDA and the EMA. The analytical distinction between an orphan drug (a specific active substance with at least one orphan designation) and an orphan drug approval, which refers to the drug approval for orphan-designated indications, provides important granularity. Both the FDA and the EMA allow drugs under development to be granted orphan designation prior to approval for marketing. Previous studies comparing orphan drugs between the FDA and the EMA have often overlooked the important distinction raised by multiple indications. Ignoring this distinction could result in incomplete assessments of the orphan drug landscape.

**Fig 1:**
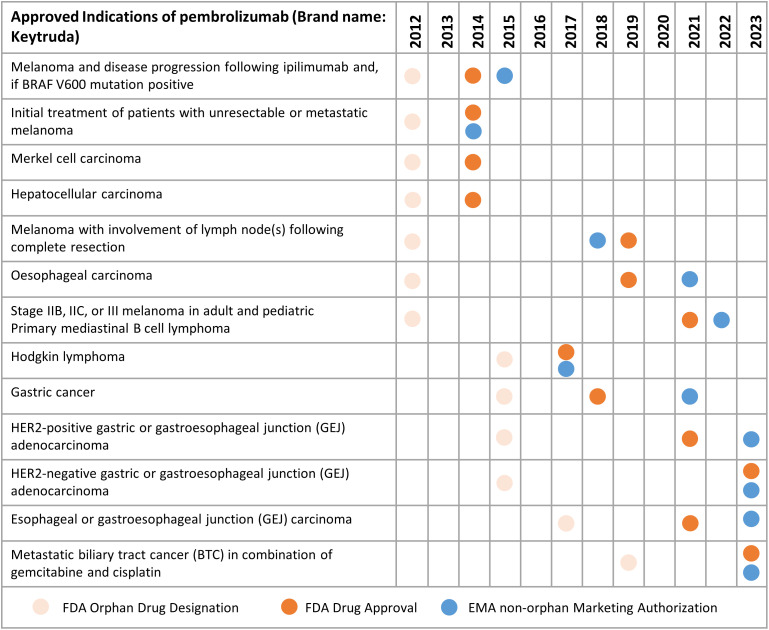
Orphan designations and approvals of pembrolizumab (brand name: Keytruda) by the United States Food and Drug Administration (FDA) and the European Medicines Agency (EMA), 2011–2023. The figure shows that pembrolizumab received FDA approval for 14 orphan-designated indications during this period, whereas the EMA authorised 11 indications (none with orphan designation) over the same timeframe.

The importance of looking at the pattern of approved indications (in contrast to orphan drugs) is illustrated in Fig 1, which shows the multiple approvals of pembrolizumab (Brand name: Keytruda) over time at the FDA and the EMA. Pembrolizumab was approved for nine indications in the period studied. No other drug had more approved indications. Despite its commercial focus on orphan indications, pembrolizumab was ranked as the top pharmaceutical product by global sales in 2023 and 2024. Pembrolizumab provides a natural experiment illustrating how differences arise between rates of orphan approvals at the FDA and the EMA, with the drug being repeatedly granted orphan authorisation in the US while obtaining fewer marketing authorisations from the EMA, and no orphan designation for any indication.

In this paper, we explore an important gap in knowledge by mapping the divergent pattern of orphan designations and approvals between the FDA and the EMA for all therapeutic areas between 2011 and 2023 and seek to explain the observed differences. The paper addresses two research questions: firstly, which factors were associated with non-orphan EMA marketing authorisations; and second, which factors were associated with the absence of EMA marketing authorisation. The implications of our results are discussed in the context of recent policy proposals in the EU to improve patient access to treatments for rare diseases [2].

## Methods

### Study cohort

We provide analysis of a retrospective cohort of orphan approvals granted by the FDA between 1 January 2011 and 31 December 2023, to determine their EMA regulatory status. The study period was selected to provide a recent cohort of FDA orphan drug approvals while allowing sufficient follow-up time to examine their corresponding regulatory status at the EMA. Key information extracted from the publicly accessible dataset of the FDA (Orphan Drug Designations and Approvals) include generic and brand name, indication, sponsor, date of orphan designation, and approval date. The FDA-approved indications which had been withdrawn and approved indications with designations withdrawn or revoked were excluded from the dataset. To determine whether the FDA orphan approvals were designated and authorised by the EMA during the same period, we then searched the Union Register of medicinal products dataset and European Public Assessment Reports (EPARs) to check the related orphan designation and marketing authorisations. The withdrawn EMA market authorisation applications were cross-checked with Committee for Medicinal Products for Human Use (CHMP) reports. We only analyse drugs that received centralised approval by the EMA. We do not assess drugs approved through national, decentralised, or mutual recognition procedures, as these were outside the scope of our research. This focus is justified by the requirement that drugs with an orphan designation must undergo the centralised procedure, and the fact that the majority of new, innovative medicines were evaluated by the EMA through this process [6].

The FDA and the EMA follow distinct regulatory pathways for orphan drugs. In the United States, the FDA’s Office of Orphan Products Development grants orphan designation and marketing approval through separate but closely linked processes. In contrast, the EMA applies a two-step framework: the Committee for Orphan Medicinal Products (COMP) grants orphan designation prior to the marketing authorisation application and re-evaluates eligibility at the time of marketing authorisation. Orphan designation may be withdrawn either during this reassessment or post-authorisation if the criteria are no longer met. This sequential process underpins our two-stage modelling framework, in which Stage 1 estimates the likelihood of any EMA marketing authorisation (orphan and non-orphan) and Stage 2, conditional on EMA marketing authorisation, estimates the likelihood of being orphan designated. The EMA regulatory status is classified into three categories based on whether FDA orphan approvals were correspondingly designated and authorised by the EMA, or not (Table 1).

**Table 1:** Categories of European Medicines Agency (EMA) regulatory status applied to United States Food and Drug Administration (FDA) orphan approvals from 2011 to 2023.

EMA Status	Explanation
Orphan Marketing Authorisation	FDA orphan approvals also authorised by the EMA for an orphan-designated indication by the end of 2023. This includes products with active orphan status during the period from 2011 to 2023 that had expired before 2024.
Non-orphan marketing authorisation	FDA orphan approvals authorised for marketing by the EMA without orphan designation, or with orphan designations withdrawn before marketing authorisation, at the time of marketing authorisation, or after marketing authorisation by the end of 2023.
Not Authorised	FDA orphan approvals that did not have marketing authorisation by the EMA before the end of 2023, for the following reasons: (1) an application for marketing authorisation was not submitted to the EMA, (2) an application was withdrawn by the sponsor during marketing authorisation application, (3) the application was refused, or 4) the marketing authorisation was granted but withdrawn later.

Prior research observed a delay in EMA orphan marketing authorisations, relative to the FDA, with a median time lag for designation of three months and median time lag of six months for marketing authorisation [7]. Our results primarily focussed on the EMA regulatory status of the study cohort up to the end of 2023. We compared FDA and EMA regulatory processes and outcomes within the same time window to permit the most direct, like-for-like comparison feasible from regulatory disclosures.

In order to maintain cross-agency symmetry and minimise misclassification of time-varying regulatory states, we excluded FDA approvals that had been withdrawn or revoked. This exclusion may introduce selection bias by under-representing products with complex approval histories (e.g., safety-related withdrawals or strategic discontinuations), potentially biasing estimates toward more enduring approvals. Nonetheless, these cases represented a very small proportion (<2%) of the dataset.

### Regulatory approvals by company size, headquarters location, therapeutic areas, and period

The study characterises differences between agency regulations and identifies company- and product-level factors associated with EMA outcomes at the indication level. Independent variables were pre-specified based on prior literature examining determinants of regulatory approval and orphan drug development including company size, headquarters (HQ) location, therapeutic area and time period.

The responses of various types of companies to the same choice of market opportunity may play a role as there are major differences in the behaviour, strategies, and capabilities of different-sized companies. Large pharmaceutical companies have long dominated the development and marketing of medicines in particular therapeutic areas, while small and medium-sized enterprises (SMEs) involved in Research and Development (R&D) may have more limited experience and resources to bring products to market. Yet the division of labour in drug development between large and smaller companies is also shifting rapidly, with smaller companies playing a greater role over time [8]. However, smaller companies tend to target areas with lower returns, whereas larger companies focus on higher-return opportunities [9]. Larger companies might be expected to more readily deal with complex regulatory processes due to their greater financial resilience, experience, resources, and established credibility. Large pharmaceutical companies are also able to absorb delays, navigate regulatory complexity, and adapt product development following regulatory feedback [10]. Our analysis categorised the size of companies into three groups based on 2023 annual pharmaceutical sales from Scrip100: Tier 1 (large, *n* = 22; > $10B in annual pharmaceutical sales), Tier 2 (medium, *n* = 27; $1–$10B), and Tier 3 (small, *n* = 156; < $1B) (S1 Table).

Independent of company size, headquarters location may also contribute to the observed gap between the FDA and the EMA approval rates. Both the FDA and the EMA issued more approvals to companies headquartered in their own regions and fewer to firms based elsewhere [11]. We therefore used Citeline’s Pharmaprojects [12] database to determine for each drug the sponsor and its headquarters country in 2023.

The literature also identifies regulatory differences that result in different outcomes for the same drug across jurisdictions [13–16]. Comparative research focussed on specific therapeutic areas, such as oncology, and product types, such as Advanced Therapy Medicinal Products (ATMPs) shows notable differences. The FDA has approved more oncology drugs with orphan designation than the EMA [17], while the EMA authorised more ATMPs with orphan designation than the FDA [13]. The EMA has also been reported as slower than the FDA in granting orphan approvals [18]. In a comparison of cancer drug applications reviewed by the FDA and the EMA, there was frequent discordance between these agencies in approvals and use of expedited programs [19]. Our analysis of the therapeutic area of each approval was based on the World Health Organization’s Anatomical Therapeutic Chemical (ATC) Index 2022. We further grouped the therapeutic areas into cancer (ATC L01–L03) versus non-cancer (L04 and all other ATC groups) [19,20].

We pre-specified approval year indicator (2011–2016 versus 2017–2023) to capture the regulatory and scientific shifts, including in 2016 the launch of the EMA’s PRIME scheme [21] and updates to orphan designation guidance [22] which tightened the interpretation of “significant benefit” and imposed stricter conditions on orphan designation for biomarker-defined subsets. In parallel, US legislation and regulatory initiatives, specifically the 21st Century Act [23] in 2016 and Orphan Drug Modernization Plan [24] in 2017,promoted the growing prominence of biomarker-driven development [25–27]. The FDA’s 2017 tissue/site-agnostic approval of pembrolizumab for unresectable or metastatic MSI-H or mismatch repair-deficient solid tumours, exemplified this shift, representing the first approval based on a genomic biomarker across tumour types [28], while there had been virtually no recent EMA orphan designations successfully based on biomarker subsetting [29].

### Statistical analysis

Descriptive statistical analysis summarised FDA regulatory outcomes by therapeutic area, company size, headquarters location, and approval period. The regulatory outcomes of FDA orphan approvals at the EMA were analysed to examine the influence of these variables on regulatory outcome. This analysis aimed (1) to quantify the contribution of each variable to differences in EMA regulatory status, and (2) to assess whether these variables were associated with the probability of different regulatory outcomes.

Our previously specified two-stage modelling framework mirrors the sequential EMA decision process for FDA orphan approvals. We used a univariable logistic regression model to examine the association between the outcome and each explanatory variable individually. This approach provided a transparent, descriptive assessment of bivariate relationships rather than constructing a causal or predictive model. Because multiple EMA marketing authorisations were attributed to the same company (814 approvals developed by 204 companies), observations are not statistically independent. We therefore used logistic regression models fitted with generalised estimating equations (GEE) to account for within-company clustering. Stage 1 used univariable logistic regression fitted with GEE to examine the association between pre-specified variables and the likelihood of any EMA marketing authorisation. Stage 2, restricted to EMA authorised FDA orphan approvals, assessed the associations between these variables and the likelihood of orphan versus non-orphan authorisation. Both models included pre-specified variables—period (2011–2016 versus 2017–2023), therapeutic area (cancer versus non-cancer), company size (large, medium, small), and company location (EU, US, Other).

This study is reported as per the Strengthening the Reporting of Observational Studies in Epidemiology (STROBE) guideline (S1 Checklist). All analyses were conducted in R (version 4.4.3). Estimates were from univariable GEE logistic regression models accounting for clustering at the company level. We consider two-tailed *p* values <0.05 statistically significant and report 95% confidence intervals (CI) throughout. We present detailed data and regression results in S2 and S3 Tables.

## Results

**Fig 2:**
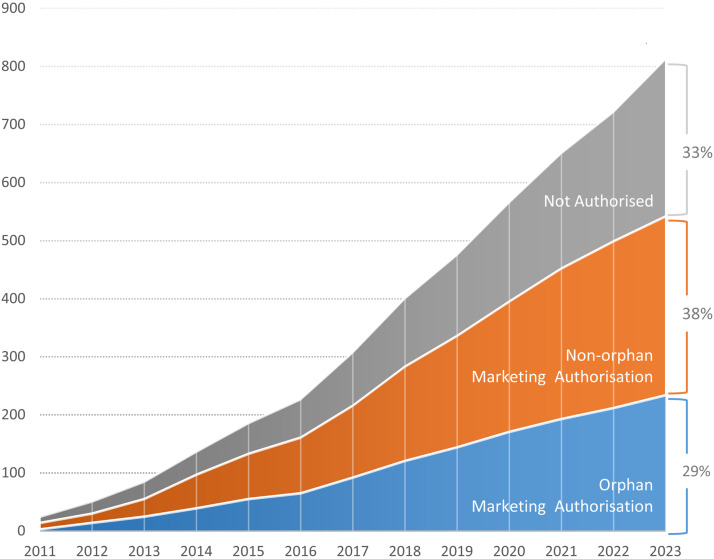
Cumulative number of United States (US) orphan drug approvals by European Medicines Agency (EMA) regulatory outcome, 2011–2023. X-axis: year of approval by the United States Food and Drug Administration (FDA). Y-axis: cumulative number of products in each EMA regulatory category by the end of 2023.

Between 2011 and 2023, the FDA granted 814 orphan approvals. Fig 2 shows the corresponding status for this cohort at the EMA. By the end of 2023, 235 (29%) of these cases were designated and authorised for orphan indications by the EMA. A further 308 (38%) of the FDA orphan approvals were authorised by the EMA without orphan designation, while the remaining 271 (33%) were not authorised by the EMA for the relevant indication. The number of annual FDA orphan approvals increased approximately 3-fold during the studied decade, from 26 in 2011 to 91 in 2023. To assess temporal trends, we examined two pre-specified periods. Between 2011 and 2016 an annual mean of 11 FDA orphan approvals were designated and authorised by the EMA. This increased to an annual mean of 24 between 2017 and 2023. However, cases of FDA orphan approval not being authorised by the EMA rose more quickly from an annual mean of 11 in the first period, to 29 annually in the second period. The net effect of these differences is that while 543 (67%) of FDA orphan approvals were also granted marketing authorisation by the EMA, 57% of these obtained authorisation without orphan designation.

For those FDA orphan approvals not authorised in the EU, we find that only 9 (3%) of these cases were refused by the EMA, while in 11 (4%) cases the marketing authorisation application was withdrawn, 17 (8%) were withdrawn from use after authorisation and 2 (1%) were suspended. Analysis in years subsequent to the 2011–2023 study period found that the lag in EMA market authorisations (relative to FDA) accounted for a further 25 marketing authorisations granted in 2024 and 5 by September in 2025. This leaves 75% (202/271) of the FDA-EMA gap in approvals unaccounted for. The elimination of other explanations (i.e., the refusals, withdrawals, and lagged marketing authorisations above), leads us to conclude that most FDA orphan approvals were not authorised by the EMA because they were never submitted to the agency by sponsoring companies for the relevant indication. In other words, companies did not seek marketing authorisation in Europe, perhaps due to commercial reasons. Below, we explore whether company size and location are associated with these decisions.

### Approval period

During the study period, FDA orphan approvals increased at a faster rate than the corresponding outcomes at the EMA. As shown in S1 Fig, absolute FDA orphan approval numbers in 2017–2023 were 2.6 times more than those in 2011–2016. The discordance with the EMA had been growing, as FDA orphan approvals in the later period (2017–2023) had lower odds ((OR 0.66 (95% CI [0.48, 0.92]; *p* = 0.013)) of receiving EMA marketing authorisation compared with 2011–2016. However, the ratio of orphan marketing authorisations and non-orphan marketing authorisations appears stable across the two periods, with no significant association between the approval period and EMA orphan designations.

### Therapeutic focus

Differences in rates of authorisation or designation by therapeutic area may be a further important variable for the FDA/EMA approval gap. Antineoplastic agents for the treatment of cancer accounted for 43% of all orphan approvals in the US from 2011 to 2023. Neurologic and autoimmune indications accounted for 17% of FDA orphan approvals and other therapeutic areas each contributed less than 15%. It is notable that the growth in the FDA orphan cancer approvals showed a strong increase between 2011 and 2023 (Fig A in S1 Appendix), from mean annual number of 16 in the first period to 37 in the second period.

**Fig 3:**
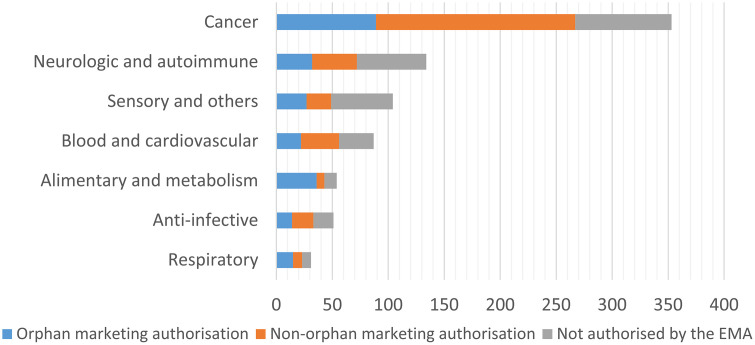
The European Medicines Agency (EMA) regulatory status of United States Food and Drug Administration (FDA) orphan approvals across therapeutic areas.

Analysis of the EMA outcomes by therapeutic area was undertaken (Fig 3). The most prominent finding is that half of the FDA orphan approvals for cancers were granted marketing authorisation by the EMA without orphan designation (50%; 178/353). One quarter (25%; 89/353) of the total were authorised with orphan designation and approximately another quarter (24%; 86/353) had no EMA marketing authorisation. Compared with cancer drugs, non-cancer drugs were associated with lower odds (OR 0.53 (95% CI [0.38, 0.75]; *p* < 0.001)) of having EMA marketing authorisation, but higher odds (OR 2.36 (95% CI [1.50, 3.70]; *p* < 0.001)) of being authorised with orphan designations.

In the non-cancer therapeutic areas, the Sensory and others category exhibited the highest proportion of non-authorised FDA approvals (53%), followed by Neurologic and autoimmune (46%). These two therapeutic areas were associated with lower odds (Sensory and others (OD 0.38 (95% CI [0.24, 0.61]; *P* < 0.001)); Neurologic and autoimmune (OD 0.34 (95% CI [0.21, 0.57]; *P* < 0.001)) of having EMA marketing authorisation compared with approvals for cancer treatments (Table A in S1 Appendix). In contrast, the area of Alimentary and metabolism diseases had the highest proportion (67%) of orphan marketing authorisations granted by the EMA, and it was associated with higher odds (OD 3.25 (95% CI [1.53, 6.90]; *P* = 0.002) of being authorised with orphan designation.

### Size of companies

**Fig 4:**
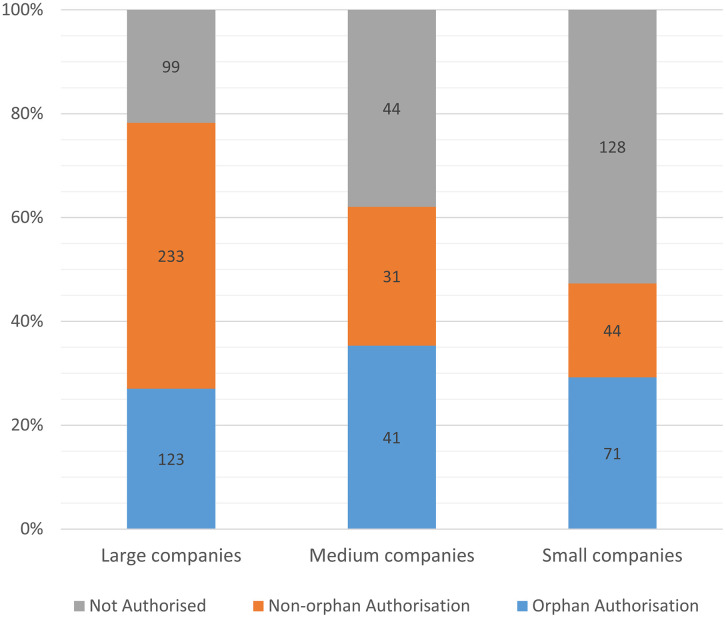
Distribution of European Medicines Agency (EMA) regulatory outcomes by company size, showing the absolute number and proportion of approvals across large, medium, and small companies.

The large (Tier 1) companies dominated orphan drug approvals contributing 56% of FDA orphan approvals. Medium (Tier 2) companies accounted for a further 14%, while the remaining 30% of FDA orphan approvals were held by relatively small (Tier 3) companies. Fig 4 summarises the results.

Compared with large companies, small companies (OR 0.29 (95% CI [0.20, 0.43]; *p* < 0.001)) and medium companies (OR 0.45 (95% CI [0.28, 0.73]; *p* = 0.001)) were associated with lower odds of obtaining EMA marketing authorisation. Conversely, small companies (OR 2.95 (95% CI [1.75, 4.99]; *p* < 0.001)) and medium companies (OR 2.14 (95% CI 1.17–3.90; *p* = 0.014)) were associated with greater odds of obtaining EMA marketing authorisation with orphan designation. Further examination of company size across therapeutic areas showed that large companies accounted for the majority of FDA orphan approvals in cancer (75%; 263/353). However, 57% (149/263) of these FDA orphan cancer approvals sponsored by large companies were authorised by the EMA without receiving orphan designation. In contrast, 43% (28/65) of FDA orphan approvals for cancer indications sponsored by small companies were not authorised by the EMA.

### Headquarters location of companies

**Fig 5:**
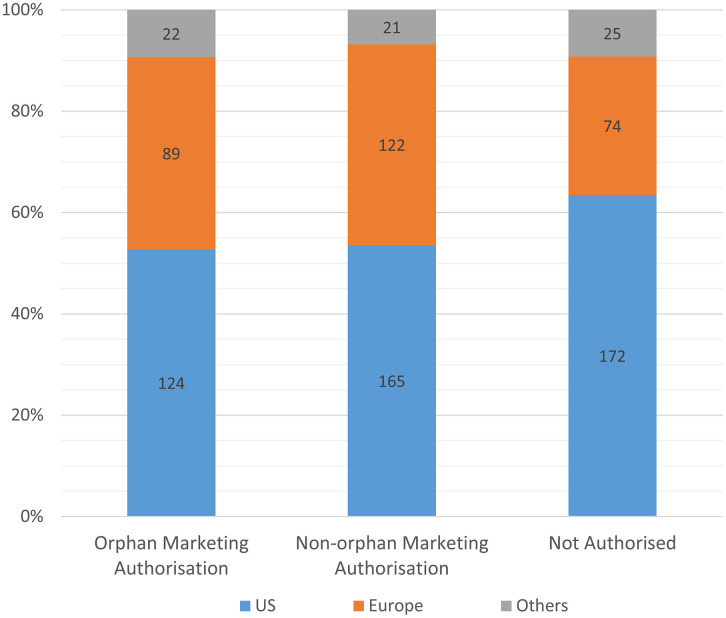
Distribution of European Medicines Agency (EMA) regulatory outcomes by sponsor headquarters (HQ) location.

The international distribution of the pharmaceutical industry was also relevant to the gap in FDA/EMA orphan approvals, and provides further context for the approval trends observed by size of companies. As shown in Fig 5, the vast majority of orphan approvals at the FDA and the EMA were accorded to companies headquartered in the US or Europe. Companies from the US contributed to 57% of FDA orphan approvals, while companies from Europe accounted for 35%. Companies from other countries, including Japan, Israel, Canada, Australia, China, and India, sponsored only 8% of FDA orphan approvals.

When considering the size of companies with orphan approvals, the composition of the US and European companies could be seen to differ in a consequential manner. While the US and Europe had a similar number of large companies (9 versus 8, respectively) and medium companies (9 US and 12 Europe), while of the small companies 109 were based in the US, compared to just 38 based in Europe. We therefore examined the influence of the company’s HQ geographical location on approval rates.

Of the 461 FDA orphan approvals in the cohort sponsored by US companies 37% (172/461) were not authorised by the EMA, while the proportion was 26% for European companies. Compared with products developed by US companies, those from Europe-based companies showed higher odds of receiving EMA marketing authorisation (OR 1.69 (95% CI [1.17, 2.43]; *p* = 0.005)), whereas no statistically significant difference was observed for companies headquartered in other regions. Moreover, a substantial proportion (60%; 103/172) of FDA orphan approvals sponsored by US companies that were not authorised by the EMA were from small US companies. Where European companies had FDA orphan approvals, these were more likely (31%; 74/285) to have a corresponding EMA marketing authorisation than US companies (27%; 124/461); but this difference was not statistically significant.

## Discussion

Comparison of the rates of orphan approval by the FDA and the EMA between 2011 and 2023 revealed two key trends. Firstly, while both regulators have increased annual orphan approvals, there is growing discordance between them in the observed outcomes for specific indications. Overall, only 29% of FDA orphan approvals had corresponding orphan marketing authorisation from the EMA. Secondly, a large and increasing number of FDA orphan approvals were being granted marketing authorisation by the EMA without a corresponding orphan designation (38%) or were not authorised for that indication (33%).

In assessing which factors were associated with non-orphan EMA marketing authorisation, our results show that rare cancer drugs were less likely to receive EMA authorisation with orphan designation than drugs in non-cancer therapeutic areas. As precision oncology has become increasingly dependent on biomarkers and the analysis of mutations, differences in regulatory policy governing biomarker use has assumed an increasingly important explanatory role in observed discordance [30,31]. Vokinger and Kesselheim found that when comparing orphan drug designations for cancer treatments in the US and EU, the FDA approved more cancer drugs with orphan designation for biomarker-targeted subgroups of prevalent cancers than the EMA [8]. A regulatory difference of growing importance seems to be FDA’s much greater acceptance in orphan designation of biomarker-defined rare subsets of more common conditions [32,33]. In contrast, the EMA has more stringent criteria to designate drugs for biomarker-based subsets; one being the ‘plausible link to the broader sub-set condition’ and another is the ‘exclusion of effects outside the subset’ [29,34].

Our results also show that large companies were less likely to obtain marketing authorisations with orphan designation, suggesting that large firms rely less on orphan incentives when entering the EU market. This implies that the 10-year period of market exclusivity already provided may not be the main determinant of company behaviour, especially large companies, when choosing where to launch an orphan product. Firstly, companies may be more dependent on patent protection to avoid competition as only a small proportion of orphan drugs had regulatory exclusivity extending beyond their patent protection [35]. This is because the advantages of orphan incentives may be outweighed by the pursuit of extended non-orphan indications, and the expansion of marketing authorisation across multiple geographic markets [36]. Large companies are particularly well positioned to capitalise on these strategies, given their greater financial resources and experience in managing multi-indication and multi-jurisdictional regulatory portfolios and running clinical trials across the globe [37,38]. Secondly, exclusivity offers legal protection, but not a guarantee of commercial return, which also depends on pricing and reimbursement decisions, Health Technology Assessment (HTA) outcomes, market size, and distribution/access constraints.

In understanding which factors were associated with the absence of EMA marketing authorisation, our findings highlight the importance of therapeutic area, company size and HQ location. Cancer drugs are more likely to be authorised by the EMA than non-cancer drugs. This reflects regulatory prioritisation of oncology, including access to expedited pathways, and stronger commercial incentives linked to high prices [39,40]. Notably, very few FDA approved orphan drugs were rejected by EMA. Commercial behaviour, particularly associated with company size, may be important in explaining observed discordance in marketing authorisations. Our analysis shows that small companies in general, and US small companies in particular, were less likely to submit for marketing authorisation in Europe when they had a corresponding FDA orphan approval. Yet these companies contribute a growing share of new drugs over time [14]. An explanation may be that small companies are at a competitive disadvantage compared to large companies in terms of resources and expertise [41]. Another important reason for companies withholding their products in Europe is the combination of complexity around reimbursement and relatively lower expected returns. Previous research has found that ‘government-imposed price controls’ were one of the main causes of pharmaceutical innovation lag between the US and the EU, with price and reimbursement regulations delaying the adoption of new pharmaceutical products [42,43]. Furthermore, the fragmented nature of the European healthcare system imposes relatively higher transaction costs on market entry, making it commercially unattractive for products targeting very small patient populations. These are substantial challenges that sit beyond the EMA's role.

Our findings raise important policy questions about orphan product regulation in the US and EU. How effective is EU regulatory policy in incentivising the launch of drugs for rare diseases and are European patients missing out on treatments available in the US? The challenge for EMA regulation is to ensure the availability of new medicines for rare diseases using targeted regulatory incentives. Policy in the study period 2011–2023 was successful in ensuring that the majority (67%) of US orphan approvals were launched in Europe. However, differences in availability are not solely due to European regulatory policy and may also arise from FDA approving a greater number of orphan products, many on the basis of less certain evidence [44]. With 33% of FDA orphan approvals not authorised for corresponding indications in Europe, further research is required to quantify the extent to which European patients lack access to orphan medicines with high therapeutic value available elsewhere. Our findings also cast doubt on the likely effectiveness of recent reforms to EU pharmaceutical legislation. These changes seek to improve access to drugs for rare diseases by creating a modular form of regulatory exclusivity based on unmet need and therapeutic impact, replacing the current one-size-fits-all incentive [45]. However, our findings suggest that orphan incentives are not the main determinant of company behaviour in launching new products in the EU and may fail to improve access or address the growing discordance with the US, particularly where commercial decisions are based on demand that is mediated by HTA and reimbursement decisions in the national markets that the EMA serves.

This study has a number of limitations. Firstly, we focus on FDA orphan drug approvals and their regulatory status under the EMA, exploring the factors contributing to the increasing discordance. We did not report on the reverse scenario, due to less observed disparity and much smaller numbers (see S1 Text). Secondly, this study could not systematically examine jurisdiction-specific regulatory criteria such as the EU requirement to demonstrate significant benefit or the FDA’s acceptance of sub-setting or stage-specific conditions. Although our findings suggest that differences in orphan drug criteria may contribute to the observed discordance, the available dataset does not consistently provide structured data on regulatory rationales. Finally, our findings apply specifically to orphan medicines and should not be generalised to treatments for common diseases. Future research should examine discordance in approval rates while taking into account therapeutic value, pricing and reimbursement, preferably while encompassing both orphan and non-orphan drugs.

## Supporting information

S1 Table Large and Medium Companies by Pharma Sales in 2023. (PDF)

S2 Table Characteristics of United States Food and Drug Administration (FDA) orphan approvals, 2011–2023. Values are numbers (percentages).
(PDF)

S3 Table Multivariable logistic regression analysis of the European Medicines Agency (EMA) regulatory status of United States (US) orphan approvals, 2011–2023. Values are odds ratios (95% confidence intervals).
(PDF)

S1 Checklist Strengthening the Reporting of Observational Studies in Epidemiology (STROBE) Checklist. This checklist is adapted from the STROBE Statement, available from the STROBE Initiative (https://www.strobe-statement.org/) and is licensed under a Creative Commons Attribution 4.0 International license (https://creativecommons.org/licenses/by/4.0/).
(PDF)

S1 Fig Orphan drug approvals by the United States Food and Drug Administration (FDA) and Corresponding European Medicines Agency (EMA) regulatory outcomes, 2011–2016 and 2017–2023. (PDF)

S1 Appendix Therapeutic areas of United States Food and Drug Administration (FDA) orphan drug approvals and related European Medicines Agency (EMA) regulatory outcomes, 2011–2023. (PDF)

S1 Text The study on the Food and Drug Administration (FDA) regulatory status of European Medicines Agency (EMA) orphan marketing authorisations from 2011 to 2023. (PDF)

## Abbreviations

ATC: Anatomical Therapeutic Chemical
ATMPs: Advanced Therapy Medicinal Products
CHMP: Committee for Medicinal Products for Human Use
CI: confidence interval
COMP: Committee for Orphan Medicinal Products
EMA: European Medicines Agency
EPARs: European Public Assessment Reports
EU: European Union
FDA: Food and Drug Administration
GEE: generalised estimating equations
HQ: headquarters
HTA: Health Technology Assessment
ODA: Orphan Drug Act
R&D: Research and Development
SMEs: small and medium-sized enterprises
US: United States
