# Supplementary material for: Discordance in orphan drug approvals between the U.S. Food and Drug Administration and the European Medicines Agency: A retrospective observational analysis
Source: PLoS Med. 2026 Jul 6;23(7):e1004861. doi: 10.1371/journal.pmed.1004861 (PMC13375132; doi:10.1371/journal.pmed.1004861)
Supplement: S1 Table — (PDF) [file pmed.1004861.s001.pdf]

S1 Table. Large and Medium Companies by Pharma Sales in 2023.

Company	HQ Location	Pharma Sales Rank	Pharma Sales Value ($M)	Number of FDA Orphan Approvals
**Large**				
Pfizer	United States	1	58,496	38
AbbVie	United States	2	53,842	18
Merck&Co	United States	3	53,583	20
Johnson & Johnson	United States	4	53,267	18
Roche	Switzerland	5	49,688	49
Novartis	Switzerland	6	45,440	52
Bristol Myers Squibb	United States	7	44,398	62
AstraZeneca	United Kingdom	8	43,789	38
Sanofi	France	9	40,985	12
GSK	United Kingdom	10	37,724	10
Eli Lilly	United States	11	34,124	11
Novo Nordisk	Denmark	12	33,722	5
Takeda	Japan	13	29,482	26
Amgen	United States	14	28,190	32
Gilead Sciences	United States	15	27,116	23
Boehringer Ingelheim	Germany	16	22,480	7
Teva	Israel	17	15,844	3
Viatris	United States	18	15,252	2
Bayer	Germany	19	14,994	8
CSL	Australia	20	14,100	9
Astellas	Japan	21	11,108	12
Daiichi Sankyo	Japan	22	10,027	3
**Medium**				
Vertex	United States	23	9,869	23
Otsuka	Japan	24	9,707	2
Merck KGaA	Germany	26	8,711	2
Biogen	United States	28	7,247	3
Regeneron	United States	29	7,078	7
Sun Pharmaceutical	India	33	5,749	1
UCB	Belgium	34	5,681	4
Abbott Laboratories	United States	35	5,039	1
Eisai	Japan	36	4,590	3
Fresenius	Germany	37	4,538	1
Jazz	Ireland	39	3,737	10
Ipsen	France	44	3,413	7
Chiesi	Italy	46	3,273	8
Incyte	United States	48	3,165	7
Kyowa Kirin	Japan	49	3,154	4
Mitsubishi Chemical	Japan	50	3,118	2
Lundbeck	Denmark	55	2,824	3
Ferring Pharmaceuticals	Switzerland	61	2,336	1
United Therapeutics	United States	62	2,328	4
BioMarin Pharmaceutical	United States	65	2,241	6
BeiGene	China	66	2,190	1
Recordati	Italy	69	2,081	4
Mallinckrodt	Ireland	74	1,866	2
Neurocrine Biosciences	United States	75	1,861	1
Swedish Orphan Biovitrum	Sweden	76	1,783	5
Exelixis	United States	78	1,629	3
Pierre Fabre	France	89	1,303	1
