# Supplementary material for: Discordance in orphan drug approvals between the U.S. Food and Drug Administration and the European Medicines Agency: A retrospective observational analysis
Source: PLoS Med. 2026 Jul 6;23(7):e1004861. doi: 10.1371/journal.pmed.1004861 (PMC13375132; doi:10.1371/journal.pmed.1004861)
Supplement: S2 Table — Values are numbers (percentages). (PDF) [file pmed.1004861.s002.pdf]

S2 Table. Characteristics of United States Food and Drug Administration (FDA) orphan approvals, 2011–2023. Values are numbers (percentages).

Characteristics	EMA Orphan Marketing Authorisation	EMA Non-orphan Marketing Authorisation	Not Authorised by the EMA
**Period**			
2011-2016	66(28)	96(31)	66(24)
2017-2023	169(72)	212(69)	205(76)
**Therapeutic area**			
Cancer	89(38)	178(58)	86(32)
Non-cancer	146(62)	130(42)	185(68)
**Company size**			
Large	123(52)	233(76)	99(37)
Medium	41(17)	31(10)	44(16)
Small	71(30)	44(14)	128(47)
**Company HQ Location**			
US	124(53)	165(54)	172(64)
Europe	89(38)	122(40)	74(27)
Other	22(9)	21(6)	25(9)
