# Supplementary material for: Discordance in orphan drug approvals between the U.S. Food and Drug Administration and the European Medicines Agency: A retrospective observational analysis
Source: PLoS Med. 2026 Jul 6;23(7):e1004861. doi: 10.1371/journal.pmed.1004861 (PMC13375132; doi:10.1371/journal.pmed.1004861)
Supplement: S3 Table — Values are odds ratios (95% confidence intervals). (PDF) [file pmed.1004861.s003.pdf]

S3 Table. Multivariable logistic regression analysis of the European Medicines Agency (EMA) regulatory status of United States (US) orphan approvals, 2011–2023. Values are odds ratios (95% confidence intervals).

	Odds ratios of FDA orphan approvals authorised by the EMA	P value	Odds ratios of FDA orphan approvals authorised with orphan designations by the EMA	P value
**Period**				
2011-2016	1(reference)		1 (reference)	
2017-2023	OR 0.66 (95% CI [0.48, 0.92])	0.013	OR 1.17, 95% CI [0.86, 1.60])	0.314
**Therapeutic area**				
Cancer	1(reference)		1(reference)	
Non-cancer	OR 0.53 (95% CI [0.38, 0.75])	<0.001	OR 2.36 (95% CI [1.50, 3.70])	<0.001
**Company size**				
Large	1(reference)		1(reference)	
Medium	OR 0.45 (95% CI [0.28, 0.74])	0.001	OR 2.14 (95% CI [1.17, 3.90])	0.014
Small	OR 0.29 (95% CI [0.20, 0.43])	<0.001	OR 2.95 (95% CI [1.75, 4.99])	<0.001
**Company HQ location**				
US	1(reference)		1(reference)	
Europe	OR 1.69 (95% CI [1.17, 2.43])	0.005	OR 1.31 (95% CI [0.85, 2.02])	0.223
Other	OR 1.22 (95% CI [0.68, 2.19])	0.507	OR 1.37 (95% CI [0.73, 2.56])	0.332
