# Supplementary material for: Discordance in orphan drug approvals between the U.S. Food and Drug Administration and the European Medicines Agency: A retrospective observational analysis
Source: PLoS Med. 2026 Jul 6;23(7):e1004861. doi: 10.1371/journal.pmed.1004861 (PMC13375132; doi:10.1371/journal.pmed.1004861)

**S1 Fig. Orphan drug approvals by the United States Food and Drug Administration (FDA) and Corresponding European Medicines Agency (EMA) regulatory outcomes, 2011–2016 and 2017–2023.**

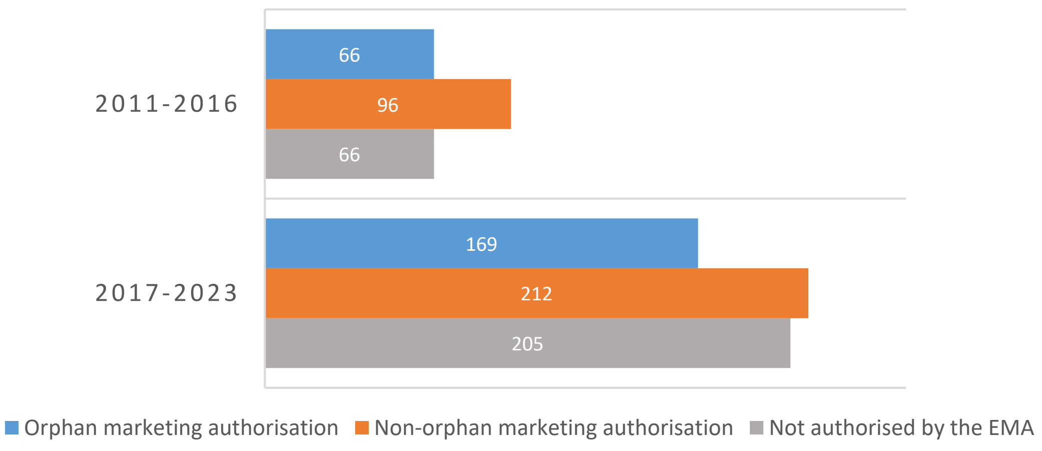

Supplement: S1 Fig — (PDF) [file pmed.1004861.s005.pdf]
