# Supplementary material for: Discordance in orphan drug approvals between the U.S. Food and Drug Administration and the European Medicines Agency: A retrospective observational analysis
Source: PLoS Med. 2026 Jul 6;23(7):e1004861. doi: 10.1371/journal.pmed.1004861 (PMC13375132; doi:10.1371/journal.pmed.1004861)
Supplement: S1 Appendix — (PDF) [file pmed.1004861.s006.pdf]

S1 Appendix. Therapeutic areas of United States Food and Drug Administration (FDA) orphan drug approvals and related European Medicines Agency (EMA) regulatory outcomes, 2011-2023.

Fig A. Distribution of United States Food and Drug Administration (FDA) orphan approvals across therapeutic areas, 2011–2023.

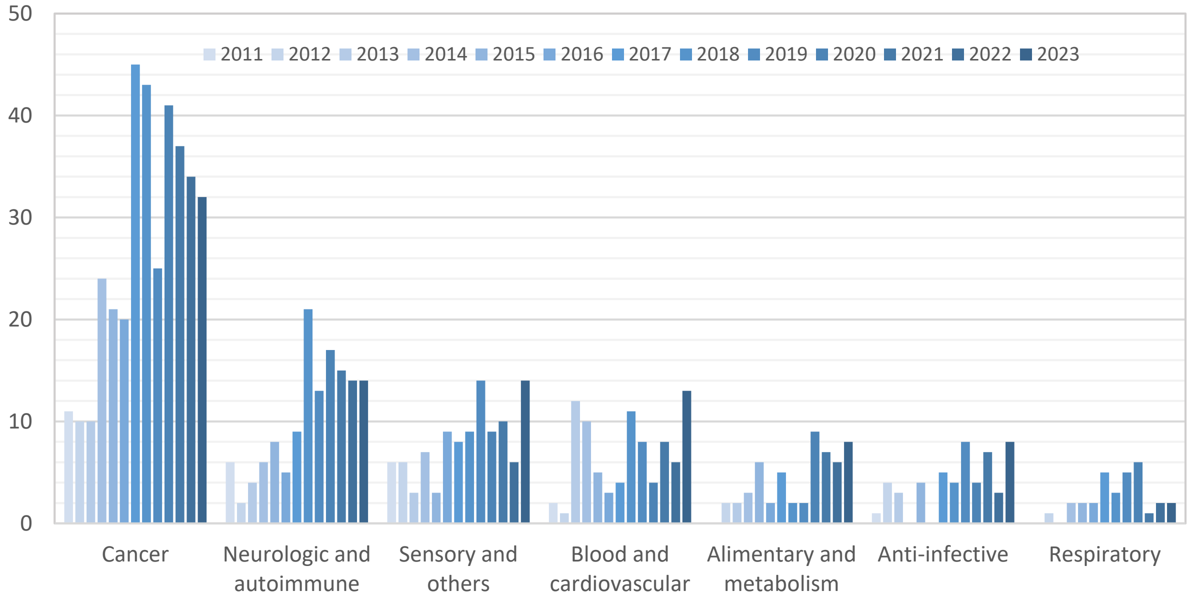

Table A. Univariable GEE logistic regression analysis among therapeutic areas, 2011–2023. Values are odds ratios (95% confidence intervals).

	**Odds of FDA orphan approvals authorised by the EMA**	**P value**	**Odds of FDA orphan approvals authorised with orphan designations by the EMA**	**P value**
**Therapeutic area**				
Cancer	1(reference)		1(reference)	
Neurologic and autoimmune	0.38 (0.24, 0.61)	<0.001	1.30 (0.70, 2.40)	0.401
Sensory and others	0.34 (0.21, 0.57)	<0.001	1.68 (0.88, 3.21)	0.115
Blood and cardiovascular	0.74 (0.43, 1.27)	0.274	0.75 (0.40, 1.40)	0.363
Alimentary and metabolism	1.46 (0.68, 3.12)	0.331	3.25 (1.53, 6.90)	0.002
Anti-infective	0.64 (0.32, 1.25)	0.190	1.36 (0.62, 2.96)	0.444
Respiratory	0.89 (0.32, 2.52)	0.831	1.54 (0.43, 5.51)	0.503
