# Supplementary material for: Discordance in orphan drug approvals between the U.S. Food and Drug Administration and the European Medicines Agency: A retrospective observational analysis
Source: PLoS Med. 2026 Jul 6;23(7):e1004861. doi: 10.1371/journal.pmed.1004861 (PMC13375132; doi:10.1371/journal.pmed.1004861)
Supplement: S1 Text — (PDF) [file pmed.1004861.s007.pdf]

S1 Text. The study on the Food and Drug Administration (FDA) regulatory status of European Medicines Agency (EMA) orphan marketing authorisations from 2011-2023

Of the EMA marketing authorisations with orphan designation from 2011-2023, all had orphan designation from the FDA. Only 12% did not yet have FDA approval at the time of EMA authorisation. Some of the EMA orphan marketing authorisations not FDA approved were subsequently approved by the FDA in 2024–Sep 2025, reducing the contemporaneous gap to 10% (17). By contrast, the share of FDA orphan approvals not authorised by the EMA over the same period is substantially higher 71% (579), and 70% (549) when taking the EMA approval lag into account.
