# Supplementary material for: Discordance in orphan drug approvals between the U.S. Food and Drug Administration and the European Medicines Agency: A retrospective observational analysis
Source: PLoS Med. 2026 Jul 6;23(7):e1004861. doi: 10.1371/journal.pmed.1004861 (PMC13375132; doi:10.1371/journal.pmed.1004861)
Supplement: Attachment — Submitted filename: Response to Reviewers.docx [file pmed.1004861.s010.docx]

Comments from the reviewers:

**Reviewer #1: Thank you for the opportunity to review this study which concerns a relevant research topic in the field.
This is a well written and easy to read paper that acknowledges the known growing discordance between the EU and USA in a number of areas in the pharmaceutical sector. However there are a number of substantial structural and methodological concerns that will need to be addressed before this paper can be considered for publication.**

1. **It is not clear why the authors selected the period of 2011-2020, please provide some context**

We thank the reviewer for pointing this out. We confirm that we conducted the relevant background research before selecting the study period. Our background research, covering 2001–2023, revealed a pronounced widening of the gap between FDA and EMA approvals during the 2011–2023 period, which guided our decision to focus on this decade (Figure 1). The EU orphan drug regulation came into force on the 28^th^ April 2000. So we exclude 2000 and firstly look at the period from 2001 to 2020 (now extended to 2023). As shown in Figure 1, the gap between the EMA and the FDA was minimal in the earlier years, reflecting the later introduction of the EU orphan drug legislation in 2000, compared with the US framework, which has been in place since 1983. The divergence becomes more evident from the second decade (2011-2023). Due to space constraints, we did not include the full background analysis in the main text of our manuscript; however, the selection of this period was made deliberately and based on these observed trends.

|  | EMA | FDA |
| --- | --- | --- |
| 2001 | 3 | 6 |
| 2002 | 5 | 13 |
| 2003 | 5 | 12 |
| 2004 | 6 | 14 |
| 2005 | 4 | 20 |
| 2006 | 13 | 24 |
| 2007 | 16 | 16 |
| 2008 | 7 | 17 |
| 2009 | 12 | 20 |
| 2010 | 4 | 15 |
| 2011 | 5 | 26 |
| 2012 | 7 | 26 |
| 2013 | 6 | 34 |
| 2014 | 11 | 52 |
| 2015 | 12 | 49 |
| 2016 | 12 | 41 |
| 2017 | 15 | 81 |
| 2018 | 21 | 93 |
| 2019 | 5 | 75 |
| 2020 | 21 | 90 |
| 2021 | 19 | 85 |
| 2022 | 31 | 71 |
| 2023 | 12 | 91 |

**Figure 1** The annual orphan approvals/marketing authorisations in FDA and EMA from 2001 to 2023

Following reviewer feedback, our study timeframe was extended from 2020 to 2023 to capture the most recent three years and emerging trends. We further incorporated 2024 and 2025 data to examine potential approval delays between regulatory agencies. Because pharmaceutical companies typically submit marketing applications to the FDA before the EMA, extending the timeframe allowed us to identify drugs that received FDA approval near the end of the research period (eg. 2021, 2022, and 2023) and were subsequently authorised by the EMA in the following years (2024 and 2025). The existence of this lag means that we cannot make fair comparisons of outcomes fully up to the current year due to a portion of recent FDA approvals still being under review at EMA. We followed the EMA orphan designation and marketing authorisation until September 2025 to reflect the lag and incorporated the delay into the sensitivity analysis.

2. **A major issue with the paper is that based on the methodology, it is difficult to conclude on what is orphan specific and what is not related to orphans. Therefore comparison to non-orphan status in order to determine the true factors that are specific to orphans is needed. Please comment on the feasibility of conducting this analysis**

We appreciate the reviewer’s suggestion. While we agree that comparing orphan and non-orphan products can help isolate what is truly “orphan-specific,” orphan designation constitutes a distinct regulatory pathway with its own aims, incentives, and evidentiary requirements. Our study was intentionally scoped to characterise within-orphan trends across agencies; the datasets we assembled do not contain a like-for-like, harmonised non-orphan cohort over the same years and jurisdictions. Introducing such a comparator would require a fundamentally different study design, expanded data collection, and different analytic methods—changes that would substantially alter the paper’s focus and scale. For the reasons we list below, such an analysis would be complex and difficult to undertake.

Our classification framework is built at the indication level, which—to our knowledge—is the first to systematically align FDA and EMA outcomes per indication and then sort FDA orphan approvals into three mutually exclusive EMA categories: (i) designated and authorised (orphan marketing authorisation), (ii) authorised without designation (non-orphan marketing authorisation), and (iii) not authorised. This lens is essential because an FDA orphan approval cohort can include EMA approvals that are non-orphan for the very same indication. In other words, the same product–indication may be “orphan” at FDA but “non-orphan” at EMA. A simple orphan vs non-orphan split across agencies therefore blurs interpretation (based on our findings, a third of the orphan marketing approvals in FDA are non-orphan in EMA), conflating regulatory classification differences with clinical or regulatory factors. Our indication-based three-way classification is the clearest way to display divergence between agencies.

Orphans now represent over half of all FDA new drug approvals, making an orphan-only analysis a substantially important task in its own right. The proposed extension would exceed the scope and complexity of prior studies in similar journals (see Table 1), which generally focus on orphan approvals only or within orphan subsets at either the FDA or the EMA, but rarely both. A smaller number compares orphan vs non-orphan, but only within a single agency (either FDA or EMA). We therefore conclude that this is not feasible for our design, because it would necessitate assembling multi-agency (FDA and EMA), multi-jurisdiction (orphan and non-orphan) datasets beyond our prespecified cohort; harmonising discordant constructs (some drugs are orphan approvals in the FDA but non-orphan marketing authorisations in the EMA) into a common data model.

**Table 1** Data-based research with focus on orphan drugs and orphan-non-orphan drugs

| Research Focus | Year | Research Period | Data source | Research Scope | Research Focus | Journal |
| --- | --- | --- | --- | --- | --- | --- |
| *Orphan only* | | | | | | |
| [FDA orphan drug designations for lysosomal storage disorders – a cross-sectional analysis \| PLOS One](https://journals.plos.org/plosone/article?id=10.1371%2Fjournal.pone.0230898) | 2020 | 1983-2019 | FDA | Orphan subset-lysosomal storage disorders | Assess the orphan drug designations and approvals | PLOS One |
| [Thirty Years of Orphan Drug Legislation and the Development of Drugs to Treat Rare Seizure Conditions: A Cross Sectional Analysis \| PLOS One](https://journals.plos.org/plosone/article?id=10.1371%2Fjournal.pone.0161660) | 2016 | -2015 | FDA, EMA | Orphan subset-Epilepsy | Investigate the orphan designations and approvals by FDA and EMA | PLOS One |
| [Follow-On Indications for Orphan Drugs Related to the Inflation Reduction Act - PMC](https://pmc.ncbi.nlm.nih.gov/articles/PMC10427936/) | 2023 | 2003-2022 | FDA | Orphan drug – follow on indications | Assesses follow-on indications of orphan drugs | JAMA Network |
| [FDA Approval of Orphan Drug Indications for Pediatric Patients, 2011-2023 - PMC](https://pmc.ncbi.nlm.nih.gov/articles/PMC11791693/) | 2024 | 2011-2023 | FDA | Orphan drug – paediatric indication | Assesses orphan drugs with pediatric indication | JAMA Pediatrics |
| [FDA Approval of Orphan Drug Indications for Pediatric Patients, 2011-2023 - PMC](https://pmc.ncbi.nlm.nih.gov/articles/PMC11791693/) | 2016 | 2002-2012 | EMA | Orphan drugs | Analyses the orphan drug pipeline in the EU | Nature drug discovery |
| [Biomarker-Defined Subsets of Common Diseases: Policy and Economic Implications of Orphan Drug Act Coverage \| PLOS Medicine](https://journals.plos.org/plosmedicine/article?id=10.1371%2Fjournal.pmed.1002190&utm_source=chatgpt.com) | 2017 | 2000-2015 | FDA | Orphan drug - biomarker designed common diseases | Investigates the subset of orphan drugs targeting on the biomarker-defined subset of common diseases | PLOS Medicine |
| [Noncompletion and nonpublication of trials studying rare diseases: A cross-sectional analysis \| PLOS Medicine](https://journals.plos.org/plosmedicine/article?id=10.1371%2Fjournal.pmed.1002966&utm_source=chatgpt.com) | 2019 | 2010-2012 | Cllinicaltrials.gov | Rare disease | Analyses clinical trials for rare diseases | PLOS Medicine |
| [Application of orphan drug designation to cancer treatments (2008-2017): a comprehensive and comparative analysis of the USA and EU - PubMed](https://pubmed.ncbi.nlm.nih.gov/31601584/) | *2019* | *2008-2017* | *FDA & EMA* | *Orphan drug - cancer* | *Compares orphan drug designations* | *BMJ Open* |
| [Orphan medicinal products in Europe and United States to cover needs of patients with rare diseases: an increased common effort is to be foreseen \| Orphanet Journal of Rare Diseases \| Full Text](https://ojrd.biomedcentral.com/articles/10.1186/s13023-017-0617-1) | 2017 | -2015 | FDA/EMA | Orphan drugs | Compares the FDA and EMA orphan drug designations and approvals | Orphanet Journal of Rare Diseases |
| [Using four decades of FDA orphan drug designations to describe trends in rare disease drug development: substantial growth seen in development of drugs for rare oncologic, neurologic, and pediatric-onset diseases \| Orphanet Journal of Rare Diseases \| Full Text](https://ojrd.biomedcentral.com/articles/10.1186/s13023-021-01901-6) | 2021 | 1983-2019 | FDA | Orphan drugs | Analyzes orphan drug designation trends | Orphanet Journal of Rare Diseases |
| *Orphan and Non-orphan* | | | | | | |
| [Five-Year Sales for Newly Marketed Prescription Drugs With and Without Initial Orphan Drug Act Designation \| Law and Medicine \| JAMA \| JAMA Network](https://jamanetwork.com/journals/jama/fullarticle/2804613?utm_source=chatgpt.com) | *2023* | *2008-2016(2021)* | *FDA* | *Non-orphan/orphan - price - US* | *Compares orphan and non-orphan drug price in US* | *JAMA* |
| [Number of Patients Studied Prior to Approval of New Medicines: A Database Analysis \| PLOS Medicine](https://journals.plos.org/plosmedicine/article?id=10.1371%2Fjournal.pmed.1001407&utm_source=chatgpt.com) | 2013 | 2000-2010 | EMA | Orphan / non-orphan - clinical trial -EU | Compares patient numbers for orphan and non-orphan drugs | PLOS Medicine |
| [Characteristics of Clinical Trials to Support Approval of Orphan vs Nonorphan Drugs for Cancer \| Oncology \| JAMA \| JAMA Network](https://jamanetwork.com/journals/jama/fullarticle/900705?utm_source=chatgpt.com) | *2011* | *2004-2010* | *FDA* | *Cancer: Non-orphan/orphan – clinical trial, review, indication* | *Drugs for orphan and non-orphan focusing on FDA without comparing FDA and EMA* | *JAMA* |
| [Five-Year Sales for Newly Marketed Prescription Drugs With and Without Initial Orphan Drug Act Designation \| Law and Medicine \| JAMA \| JAMA Network](https://jamanetwork.com/journals/jama/fullarticle/2804613?utm_source=chatgpt.com) | *2023* | *2008-2016(2021)* | *FDA* | *Non-orphan/orphan - price - US* | *Compares orphan and non-orphan drug price in US* | *JAMA* |
| [FDA approval, clinical trial evidence, efficacy, epidemiology, and price for non-orphan and ultra-rare, rare, and common orphan cancer drug indications: cross sectional analysis \| The BMJ](https://www.bmj.com/content/381/bmj-2022-073242?utm_source=chatgpt.com) | *2023* | *2000-2022* | *FDA* | *Ultra area, rare and common indications -US* | *Compares FDA approved drugs ultra-rare, rare and common indications regarding approval, price and clinical trial* | *The BMJ* |

3. **The structure of the results and the discussion - conclusion should more easily and clearly pull out factors that are specific to orphans and factors that are applicable more generally to all or most pharmaceutical products e.g.  size of company**

We appreciate the reviewer’s careful reading and valuable suggestion.

The findings of our study apply specifically to orphan products and should not be extended to medicines for common diseases. In the revised manuscript, we have clarified this scope in the sections 4.3 limitations of this study. The revisions explicitly state that the results reflect the regulatory and market dynamics unique to orphan drugs.

Although variables such as company size and therapeutic area were included to provide contextual understanding, our analysis and interpretation remain focused on orphan designations and approvals. We have ensured that this focus is now clearly communicated to prevent overgeneralisation of the conclusions. We clarified this in the limitations.

*‘Thirdly, Our findings apply specifically to orphan medicines and should not be generalized to treatments for common diseases. Although we included contextual variables (e.g., company size, therapeutic area) to aid interpretation, our analyses and inferences remain centred on orphan development and regulatory outcomes.’*

4. **A major gap for a paper with a focus on the FDA and EMA, is that the paper does not address sufficiently the differences in the regulatory orphan criteria and orphan process. As an example, significant benefit is not mentioned and this is considered to be a major hurdle for the EU designation versus the USA - please discuss all the specific factors for orphan designation in detail as per your statement in line 74.**

Thanks for this helpful comment. We added that point into the discussion. However, the EMA orphan designation criteria have not been changed during the period studied. We analysed the ratio between the three categories –orphan marketing authorisation, non-orphan marketing authorisation, not authorised- over the study period - see Figure 2 - and found that there was no major change in the proportion of FDA orphan approvals designated in the three EMA categories (the decrease in orange (Non-orphan marketing authorisation) in 2023 is mainly due to the delayed MA by the EMA). This strongly suggests that there was no major change in either the behaviour of sponsors or in regulatory policy during this time.

*‘A key difference is the EU’s significant benefit requirement: if “satisfactory methods” already exist, EMA must see a clinically relevant advantage or a major contribution to patient care at designation and again at marketing authorisation whereas FDA does not require this [30,31].’*

**Figure 2** The proportion of EMA regulatory status and annual FDA orphan approvals 2011-2023

5. **Please consider the impact of the EU not allowing orphan and non-orphan status on the same marketing authorisation**

Yes, we had taken this into account in our study design. In our methodology, the analysis is based on EMA marketing authorisations (MAs). The classification was conducted to determine whether the FDA orphan-approved indications were subsequently authorised or orphan-designated by the EMA.

Each FDA approval and EMA marketing authorisation refers to the use of an active substance to a specific therapeutic indication. FDA approval and EMA marketing authorisation are functionally comparable, as both grant legal market entry for a defined indication, although they are issued by different regulatory agencies under distinct legal frameworks. For analytical purposes, the two regulatory outcomes are treated as equivalent authorisations at the indication level.

Moreover, in our research, orphan and non-orphan indications are separated at the MA level, rather than at the drug level. This approach was adopted to avoid potential inaccuracies when comparing drugs with multiple indications, particularly those including both orphan and non-orphan uses.

To our knowledge, this is the first comparative study examining FDA and EMA outcomes at the level of approved indications rather than products. By focusing on the marketing authorisation level, we can accurately determine whether each authorised indication holds an orphan designation, thereby avoiding potential misclassification and ensuring a more precise comparison between the two regulatory agencies.

6. **The methodology would benefit from a much clearer narrative and description  - e.g. line 151 states designated or authorised yet the results focus only on authorised? the section on statistical analysis is far too brief to understand what analysis was conducted and how**

Thank you for highlighting this. We have redrafted the text you have mentioned. In response to the criticism of the statistical analysis, we have substantially rewritten the Methods section to provide a clearer and more coherent narrative of the study design and analytical approach. We have expanded the methods section to describe in detail the data sources, variable definitions, and analytical procedures (including descriptive statistics, logistic regression models and sensitivity analysis), so that readers can fully understand how the analyses were conducted.

*‘2.3 Statistical analysis*

*Descriptive statistics summarise FDA and EMA regulatory outcomes by therapeutic area, sponsor size, headquarters location, and approval period. To investigate the growing discordance between approval outcomes between the FDA and EMA, we implemented a pre-specified two-stage modelling framework mirroring the sequential EMA decision process for FDA-approved orphan drugs. Stage 1 uses multivariable logistic regression to examine the association between product- and company-level characteristics and the likelihood of any EMA marketing authorisation. Stage 2, restricted to EMA authorised FDA orphan approvals, assesses the associations between these characteristics and the likelihood of orphan versus non-orphan authorisation. Both models included pre-specified covariates—therapeutic area (cancer vs non-cancer), company size (large, medium, small), company location (EU, US, Other), and period (2011–2016 vs 2017–2023). This study is reported as per the Strengthening the Reporting of Observational Studies in Epidemiology (STROBE) guideline (S2 Checklist).*

*All analyses are conducted in R (version 4.4.3). Two-tailed p values <0.05 were considered statistically significant, and 95% confidence intervals (CI) were reported throughout. When reporting results, percentages reflect relative differences in odds compared with the reference group. Detailed data and regression results are presented in S3 Table and S4 Table. Pairwise associations among categorical predictors are small to modest, with Cramér’s V ranging from 0.109 to 0.328 in model of stage 1 and from 0.094 to 0.287 in model of stage 2. Collinearity diagnostics show VIFs of 1.0–1.2 (tolerances >0.80) for model 1 and 1.0–1.01 (tolerances >0.67) for model 2, indicating minimal multicollinearity across variables. To assess the robustness of findings, we conducted sensitivity analyses. The primary analyses used an administrative cut-off date of 31 December 2023, corresponding to the FDA cohort period (2011–2023). To account for potential lags in EMA regulatory decisions, follow-up was extended through September 2025, and all analyses were repeated using this updated regulatory status. Model performance was further examined through a sensitivity analysis of the multivariable logistic regression model, assessing discrimination—Area Under the Receiver Operating Characteristic Curve (AUC)—and classification performance (sensitivity and specificity). Detailed sensitivity analysis is presented in S5 Appendix.’*

7. **Lines 201-202 - the CHMP minutes and agendas provide substantial information. Please review this source of data regarding the statement that the EMA does not disclose data on the number of submissions it receives**

Thank you for the comment. We had reviewed the CHMP reports in our study, which indeed provide substantial information on marketing authorisations, including withdrawals, refusals, and positive opinions. For example, we had counted the withdrawn application from the CHMP reports. In the new draft, we added this into methods about the data collection from CMHP and we have updated the number of refused, withdrawn during the MA application and withdrawn after MA into the results for the period from 2011 to 2023.

*‘The withdrawn EMA market authorisation applications are cross-checked with Committee for Medicinal Products for Human Use (CHMP) reports.’*

*‘For the FDA orphan approvals not authorised in the EU, our analysis shows that only 9 (3%) of these cases were due to the refusal by the EMA, while 11 (4%) were due to the withdrawal of the marketing authorisation application and 17 (8%) were withdrawn from use and 2 (1%) were suspended.’*

However, it is important to note that these reports only capture applications that have entered the CHMP review process and reached a stage of public disclosure. There remains a possibility that companies withdraw their applications before validation or being not validated, in which case the application is never recorded in CHMP agendas or minutes. To improve the clarity, we deleted that sentence as a not-validated application cannot be counted as a proper application and the number of them cannot be quantified.

8. **The fact you reviewed only centrally authorised drugs is not a study limitation - as you indicate orphan designated products are mandated CP scope in the EU  - this can be included in your methods if needs be.**

We thank the reviewer for this valuable comment. We agree that focusing on centrally authorised products is not a study limitation in this context, as orphan-designated medicines are mandated to follow the centralised procedure in the EU. We have deleted this from the limitation and clarified this point in the methods section to make our scope explicit.

*‘We only analyse drugs that received centralised approval by the EMA. We do not assess drugs approved through national, decentralised, or mutual recognition procedures, as these were outside the scope of our research. This focus is justified by the requirement that drugs with an orphan designation must undergo the centralised procedure, and the fact that the majority of new, innovative medicines are evaluated by the EMA through this process [18].’*

9. **It would be helpful to have a key (S3 and S4) and figure legend text for the S material**

Thank you for the comments. We have deleted S3 as it was less relevant.

We have added keys to S4 (now S6).

*S6 Figure. Distribution of United States Food and Drug Administration (FDA) orphan approvals across therapeutic areas, 2011–2023.*

**Reviewer #2: This study investigates the regulatory discrepancies between the FDA and EMA for orphan drug approvals. The analysis identifies factors contributing to the divergence and highlights implications for rare disease patient access in the US and EU. While the study presents valuable insights, several methodological and interpretative aspects warrant further discussion and clarification.**

**Major comments:**

1. **Methods, the authors included orphan approvals granted by the FDA between 1/1/2011 and 12/31/2020. Given that it is now 2025, it would be beneficial to include more recent data. Analyzing the period beyond 2020 could provide valuable insights into whether the divergence between the FDA and EMA has continued to grow or if their decisions have begun to align more closely. Since this data is publicly available, the authors should clarify why the most recent years were excluded and discuss how the inclusion of more recent data might affect the study's findings and conclusions.**

We thank the reviewer for this constructive comment. In response, we have extended our dataset beyond 2020 to capture the most recent trends. Specifically, we have added FDA orphan drug approvals for the years 2021, 2022, and 2023 to our detailed analysis. In addition, we examined marketing authorisation data up to September 2025 to understand the outcomes from EMA for drugs awarded FDA approvals in the final years of our study period. This is because most drug companies launch their products in the US market first and enter the EMA market later. As a consequence, a meaningful analysis of the most recent two years is not possible due to this lag. We found 25 FDA orphan approvals granted before the end of 2023 were subsequently authorised by EMA in 2024 and 5 by Sep 2025. We believe that this extended timeframe, now covering the period up to the end of 2023, strengthens our analysis by providing a more up-to-date comparison between FDA and EMA outcomes.

To test whether the growing gap persists over the whole period, we also added the time period into the regression analysis and the results show the divergence has persisted throughout the whole research period.

2. **Methods, the authors targeted a cohort of orphan approvals granted by the FDA to examine factors influencing the growing discordance in orphan approvals between the US FDA and EMA. However, this approach may introduce sample selection bias, as it excludes orphan approvals granted by the EMA but not by the FDA. These drugs also contribute to the observed discordance and could provide additional insights into regulatory differences and approval dynamics. Including or at least discussing the implications of this reverse scenario would strengthen the comprehensiveness of the analysis.**

We appreciate the reviewer’s observation regarding the potential contribution of EMA-only orphan approvals to the overall discordance. We have, in fact, conducted an analysis examining the regulatory status of EMA marketing authorisations within the FDA framework. However, we chose not to incorporate this into the current manuscript for several reasons.

First, the “reverse” gap—EMA-authorised orphan medicines lacking FDA designation/approval—is relatively small. Of the EMA marketing authorisations with orphan designation in our study period, all had orphan designation from the FDA; only 12% (21) did not yet have FDA approval at the time of EMA authorisation. 4 of those 21 were subsequently approved by the FDA in 2024–Sep 2025, reducing the contemporaneous gap to 17 of 177 (10%). By contrast, the share of FDA orphan approvals not authorised with orphan designations by the EMA over the same period is substantially higher- 71% (579/814) and 70% (549/814) considering the delay followed up to September 2025. By comparing the missing orphan approvals from each side, the reverse gap ‘EMA to FDA’ is only about 4% (21/579) and 3% (17/549) considering the delay of the ‘FDA to EMA’ gap. This has been added in to the limitations of this study.

*‘We did not examine the reverse scenario, due to less observed disparity. Of the EMA marketing authorisations with orphan designation in our study period, all had orphan designation from the FDA, which is the same as the previous research [18]. Only 12% did not yet have FDA approval at the time of EMA authorisation. Some of the EMA orphan marketing authorisations not FDA approved were subsequently approved by the FDA in 2024–Sep 2025, reducing the contemporaneous gap to 10% (17). By contrast, the share of FDA orphan approvals not authorised by the EMA over the same period is substantially higher 71% (579), and 70% (549) when taking the EMA approval lag into account.’*

Second, adding this additional dimension would substantially increase analytical complexity and risk obscuring our primary aim—identifying factors that drive the much larger FDA-to-EMA discordance. It would also alter the covariate structure: market entry into the US and the EU is influenced by different regulatory, commercial, and clinical drivers. Pooling these pathways could confound interpretation (e.g., mix distinct selection processes) and increase the risk of model misspecification. For these reasons, we propose keeping the analysis focused.

Third, the regulatory and technological contexts differ substantially for EMA-originating orphan medicines seeking US approval. For example, during the study period, certain advanced therapy medicinal products (ATMPs) were first approved—and, in some cases, only approved—by the EMA, eg. Holoclar, Strimvelis, Ebvallo… Addressing these cases would require a separate analytical framework to account for differences in regulatory pathways, technological maturity, and market environments, which lies beyond the scope of the current paper.

In summary, there are a small number of EMA indications not approved by FDA (the reverse gap). Thus constitutes only 3%-4% of the FDA-EMA gap and because of the analytical complexity in undertaking an analysis of the reverse gap was not included in this study.

3. **Methods, the study excludes FDA orphan approvals withdrawn or revoked before 2025. While this exclusion is reasonable, further discussion on potential biases this might introduce (e.g., exclusion of drugs with complex approval histories) is warranted.**

Our design excludes FDA approvals withdrawn or revoked to maintain cross-agency symmetry and prevent misclassification of time-varying regulatory states. This choice may introduce selection bias by under-representing products with complex approval histories (e.g., safety-related withdrawals or strategic discontinuations), potentially biasing results toward more durable approvals. However, the number of such cases in our dataset was very small (<2%).

A paragraph has been added to explain the exclusion in methods section:

*‘In order to maintain cross-agency symmetry and minimise misclassification of time-varying regulatory states, we excluded FDA approvals that had been withdrawn or revoked. This exclusion may introduce selection bias by under-representing products with complex approval histories (e.g., safety-related withdrawals or strategic discontinuations), potentially biasing estimates toward more durable approvals. Nonetheless, these cases represented very small proportion (<2%) of the dataset.’*

4. **Methods, the study included company size, location of headquarters, and therapeutic area as explanatory variables. However, it's unclear how these factors were chosen. Were these factors identified based on prior literature, data availability, or exploratory analysis? The selection process should be explicitly justified to ensure these variables are meaningful predictors. It is unclear whether these factors were chosen based on theoretical frameworks, prior research, or data-driven exploration. Additionally, the model does not address potential unmeasured confounders, such as pricing strategies, length of time for different approval types between FDA and EMA, or the clinical evidence supporting each drug, which could bias the results. While odds ratios provide insight into associations, the authors should consider reporting metrics like pseudo-R² or AIC/BIC to evaluate the model's fit and quantify how much variance the included factors explain. For example, if the model only explains a small proportion of the variance in EMA decisions, this suggests that many factors influencing approvals remain unmeasured. Regression models assume that all relevant variables are included or that unmeasured factors do not systematically bias results. If critical predictors like pricing strategies or clinical evidence for each drug are missing, the conclusions drawn from the regression may be incomplete or misleading. Therefore, acknowledging the potential role of omitted variables and exploring their likely impact on findings through sensitivity analyses would strengthen the validity of the conclusions drawn from the regression analysis. If the authors cannot justify why they used the logistic regression here, I would recommend just use descriptive analysis.**

Thank you for the comments. As there are several detailed questions and points raised, we have addressed them point by point below.

I. **Covariates selection:** In our study, the selection of covariates—therapeutic area, company size, and company location—was pre-specified based on prior literature and conceptual relevance rather than exploratory data fitting.

*‘Firstly, regulatory differences can result in different outcomes for the same drug across jurisdictions [9–12]. In a comparison of cancer drug applications reviewed by the FDA and EMA, there was frequent discordance between these agencies in approvals and use of expedited programs [13]. Secondly, the responses of various types of companies to the same choice of market opportunity may play a role as there are major differences in the behaviour, strategies, and capabilities of different sized companies in the pharmaceutical industry. Large pharmaceutical companies have tended to dominate the development and marketing of medicines in particular therapeutic areas, while small and medium-sized enterprises (SMEs) involved in Research and Development (R&D) may have more limited experience and resources to bring products to market. Yet the division of labour in drug development between large and smaller companies is also shifting rapidly, with smaller companies playing a greater role over time [14]. However, Smaller companies tend to target areas with lower returns, whereas larger companies focus on higher-return opportunities [15]. Larger companies might be expected to more readily deal with complex regulatory processes due to their greater financial resilience, experience, resources, and established credibility. Large pharmaceutical companies are able to absorb delays, navigate regulatory complexity, and adapt product development following regulatory feedback [16]. Independently of company size, headquarters location may also contribute to the observed gap between the FDA and EMA approval rates. Both the FDA and EMA issued more approvals to companies headquartered in their own regions and fewer to firms based elsewhere [17].’*

II. **Confounders**: We appreciate the concern regarding omitted variables (e.g., pricing strategies and drug-level clinical evidence) that may influence EMA authorization. These data are not available for the present study and could be the focus of future work. We have added into the implication part.

*‘This paper also contributes to debates about the pricing of medicines in the US and the implications of this for policy and regulation. Some analysts claim that the much higher price of orphan drugs in the US compared to Europe is justified as US healthcare payers are more willing to pay for expensive medicines [44,45] and have a larger budget for pharmaceutical expenditure [36]. As a consequence, it is suggested that US consumers are indirectly subsidising patients in other countries who benefit from new products at much lower prices than in the US [46]. This argument is contentious, with others arguing that high US prices are mainly used to increase company profits [47,48] and a large number of the drugs on the US market that have been subject to HTA demonstrate little benefit and low cost effectiveness [49]. This paper’s findings suggest that the relatively high prices set in the US, and the unwillingness of European healthcare systems to pay similar prices for medicines, is likely to be an important factor deterring some sponsors from seeking marketing approval for orphan products in the EU - rather than differences in EMA regulation or case-based decision making.’*

III. **Why logistic regression**: The outcome is binary (EMA authorisation yes/no or EMA orphan designation yes/no). Logistic regression is standard for estimating adjusted associations across categories (therapeutic area, company size, HQ location, period). We report adjusted odds ratios with 95% CIs and make no causal claims. The methods have been used in previous research investigating the association between expedited review programs and therapeutic value.

- [Association between FDA and EMA expedited approval programs and therapeutic value of new medicines: retrospective cohort study | The BMJ](https://www.bmj.com/content/371/bmj.m3434)
- [Trends in utilization of FDA expedited drug development and approval programs, 1987-2014: cohort study | The BMJ](https://www.bmj.com/content/351/bmj.h4633)

IV. **To assess the robustness of our findings**

We added a sensitivity analysis to both the Methods and Results sections, with full details provided in the Supplementary Appendix.

*‘3.6 Sensitivity Analysis*

*We assessed robustness by repeating the adjusted analysis with follow-up extended from the primary administrative end date (31 December 2023, aligned to the 2011–2023 FDA cohort) to September 2025 to account for EMA decision lags; results from stage 1 model and stage 2 model were substantively similar. The stage 1 - EMA marketing authorisation model showed fair discrimination (AUC 0.685, 95% CI 0.646–0.724) and balanced performance at the optimal threshold (sensitivity 0.657, 95% CI 0.616–0.697; specificity 0.649, 95% CI 0.589–0.706). The stage 2 – EMA orphan designation marketing authorisation model demonstrated fair but lower discrimination (AUC 0.662, 95% CI 0.616–0.709) and a more conservative operating profile, with higher specificity and lower sensitivity at the optimal threshold (specificity 0.795, 95% CI 0.746–0.839; sensitivity 0.464, 95% CI 0.399–0.530). Details of sensitivity analyses are provided in Supplementary S5 Appendix.’*

V. **Pseudo-R² or AIC/BIC**

The aim of the logistic regression is to estimate associations rather than causal effects.

In logistic regression analyses, particularly in regulatory and epidemiological research published in leading medical journals (e.g., PLOS Medicine, BMJ, JAMA, The Lancet), it is not standard practice to report these indices. Most studies in these journals focus on presenting adjusted odds ratios (aORs) with 95% confidence intervals and p-values, which directly convey the strength and direction of associations of regulatory and clinical interest. Examples can be found in Table 2.

**Table 2.** Logistic regression studies not reporting Pseudo-R², AIC, or BIC

| Association | Regression | Pseudo-R² or AIC/BIC | Reference |
| --- | --- | --- | --- |
| Association between therapeutic value and expedited review program | Logistic regression | No | Hwang TJ, Ross JS, Vokinger KN, Kesselheim AS. Association between FDA and EMA expedited approval programs and therapeutic value of new medicines: retrospective cohort study. BMJ. 2020 Oct 7;371:m3434. doi: 10.1136/bmj.m3434. PMID: 33028575; PMCID: PMC7537471. |
| Association between physicians’ participation in non-interventional post-marketing studies (NIPMS) and their prescribing behaviour | Logistic regression | No | Koch C, Schleeff J, Techen F, Wollschläger D, Schott G, Kölbel R, Lieb K. Impact of physicians' participation in non-interventional post-marketing studies on their prescription habits: A retrospective 2-armed cohort study in Germany. PLoS Med. 2020 Jun 26;17(6):e1003151. doi: 10.1371/journal.pmed.1003151. PMID: 32589633; PMCID: PMC7319278. |
| Number of modifications to the Paediatric Investigation Plan (PIP) and the likelihood of delays in paediatric trial Logistic regression completion. | Logistic regression | No | Hwang TJ, Tomasi PA, Bourgeois FT. Delays in completion and results reporting of clinical trials under the Paediatric Regulation in the European Union: A cohort study. PLoS medicine. 2018 Mar 1;15(3):e1002520. |
| Association between the calendar year of FDA approval and the odds that a newly approved drug was tied to ≥1 expedited program | Logistic regression | No | Kesselheim AS, Wang B, Franklin JM, Darrow JJ. Trends in utilization of FDA expedited drug development and approval programs, 1987-2014: cohort study. Bmj. 2015 Sep 23;351. |
| Association between factors and symptoms with Post-COVID syndrome in non-hospitalised patients with COVID-19 | Logistic regression | No | Augustin M, Schommers P, Stecher M, Dewald F, Gieselmann L, Gruell H, Horn C, Vanshylla K, Di Cristanziano V, Osebold L, Roventa M. Post-COVID syndrome in non-hospitalised patients with COVID-19: a longitudinal prospective cohort study. The Lancet Regional Health–Europe. 2021 Jul 1;6. |
| Association between factors and the withdrawal of anticancer indications with accelerated approval | Logistic regression | No | Tibau A, Cliff ER, Romano A, Borrell M, Molto C, Kesselheim AS. Predictors of withdrawal of anticancer drug indications granted accelerated approval: a retrospective cohort study. eClinicalMedicine. 2025 Jun 1;84. |

While AIC, BIC, and pseudo-R² can provide additional model diagnostics, they are primarily useful for model selection and comparative purposes, rather than for interpretation of associations in a single, prespecified model. Given the study’s objective—to estimate the adjusted associations between pre-specified independent variables (therapeutic area, company size, HQ location, period) and outcome (EMA marketing authorisation / orphan designation) —we followed prevailing reporting conventions in high-impact medical journals and present the effect estimates (ORs, 95% CIs, and p-values) as the primary results.

VI. **Descriptive transparency**

In response to the suggestion to prioritise description, we now provide descriptive tables (S3) reporting unadjusted rates and unadjusted effect sizes by therapeutic area, company size, location and period, so the core patterns are visible.

**5. Implications, the authors argue that European patients may be missing out on treatments available in the US due to the divergence in regulatory outcomes. While the findings suggest limited access to certain drugs in Europe, the extent to which European patients are significantly disadvantaged is not empirically analyzed. For instance, the authors do not quantify the clinical impact or assess the real-world effectiveness of the non-submitted or non-authorized drugs in routine care. Additionally, there is no direct evaluation of how this divergence contributes to unmet patient needs. The authors could also discuss the possibility of over-approval by the US FDA, which may lead to discrepancies in access that are not solely due to limitations in the EU system.**

Thanks for the valuable comments. We have added this point to the discussion.

*‘However, 33% of FDA orphan approvals were not authorised for corresponding indications in Europe. Further research is required to quantify the extent to which European patients lack access to orphan medicines with high therapeutic value that are available in the US. While our findings indicate that some FDA-approved treatments are not available in the EU, this does not, by itself, demonstrate systematic disadvantage for European patients. Differences in availability are not solely due to European market constraints and may also arise from FDA approving a greater number of orphan products, many on the basis of less certain evidence [40].’*

**Minor comments**

1. **Abstract, the authors could improve clarity by separating the "Methods" and "Findings" into distinct sections within the abstract. The methods section should provide additional details, such as the specific databases used, the factors included in the regression model, and whether any subgroup or sensitivity analyses were conducted. Moreover, since logistic regression is mentioned in the methods, the main findings should report key regression results, including odds ratios, 95% confidence intervals, and p-values, to support the interpretation of the results.**

In the revised abstract we have added:

- The data sources are clearly stated in the main text, while the study period (2011–2023) and follow-up through September 2025 are now specified.
- Sensitivity analysis were conducted and results were robust across sensitivity analyses.
- The odds ratios, 95% confidence intervals, and p-values were added to support the interpretation.

**2. Results, in S2 Appendix, can the authors explain the p-values for the rows corresponding to therapeutic area, company size, and company HQ location, for example Therapeutic area p=0.002 and p<0.001? Additionally, it would be helpful to include another table or add columns to the existing table to display the unadjusted percentages of FDA orphan approvals authorized by the EMA and FDA orphan approvals authorized with orphan designations by the EMA. This would allow readers to compare unadjusted and adjusted results more transparently.**

We agree that providing unadjusted descriptive results would aid interpretability. In the revised manuscript, we added an additional table (S3 Appendix) that reports the raw, unadjusted percentages of FDA orphan approvals that were (a) authorized by the EMA with orphan designations and (b) authorized without orphan designations and (c) not authorised by the EMA.

The S2 (now S4) has been updated with the new data.
