# Supplementary material for: Discordance in orphan drug approvals between the U.S. Food and Drug Administration and the European Medicines Agency: A retrospective observational analysis
Source: PLoS Med. 2026 Jul 6;23(7):e1004861. doi: 10.1371/journal.pmed.1004861 (PMC13375132; doi:10.1371/journal.pmed.1004861)
Supplement: Attachment — Submitted filename: Reponse to comments_v2.2.docx [file pmed.1004861.s012.docx]

**Reviewer #1**: Major concerns

1. I would like to thank the authors for the considerable work on improving the description of the methodology sections. This is much improved and clearer. However, I have major concerns about the over-interpretation of the research based on the data derived from this methodology. Examples include claims made about therapeutic area when in reality is only whether a medicines falls under a cancer or non-cancer category - this is insufficiently granular to claim therapeutic assessment considering the breadth of rare diseases. There are also conclusions and statements - for example 'most are not designated as orphan products due to regulatory differences regarding significant benefit and sub-setting of diseases based on biomarkers, particularly in the field of oncology' that are unsubstantiated. Although it is agreed significant benefit and subsetting are main drivers for the difference, this does not come from the current research or linked clearly to the published literature. The authors are advised to ensure that any claims made based on their data are clearly defined, where other conclusions or claims are made these should be sufficiently supported by the published literature and well justified.

We thank the reviewer for raising this important point regarding interpretation. We address this issue from two perspectives.

1. Therapeutic area:

In the Results section, Fig 3 presents the distribution of EMA regulatory status across therapeutic areas. In the appendix, S6A Figure shows FDA orphan drug approvals across therapeutic areas from 2011 to 2023, and newly added S6B Table represents the logistic regression results across therapeutic areas. We have added revisions in the main text to present the results in a more granular manner.

**Please check lines [295-301] and S6 Appendix.**

2. Subsetting:

Thank you for this comment. We agree that conclusions drawn directly from our data should be clearly distinguished from interpretive discussion. We have therefore completely revised the Discussion, making this much shorter and ensuring that statements regarding disease sub-setting and biomarker-defined populations are explicitly framed as contextual interpretation rather than findings of the present analysis, and supported by appropriate references.

**Please check lines [365-374].**

Other issues include:

2. Orphan approvals terminology within the text remains difficult to follow - this needs to be clearer for the reader. Table 1 is helpful but could better define to EMA description and FDA status

Thank you for this comment. We recognise that the terminology surrounding orphan approvals from FDA and/or EMA can be difficult to follow, and so we had made efforts throughout the manuscript to define these terms clearly, improve the flow of the text, and reduce potential ambiguities. However, the current terminology and structure ties to the study’s design, in which the FDA orphan approval cohort (2011-2023) serves as the analytical starting point and corresponding EMA status is subsequently assessed. We believe that reversing the structure would be more likely to introduce ambiguity for the reader, rather than improve clarity. It is worth noting that we conducted a reverse analysis investigating the FDA regulatory status of EMA marketing authorisations, which follows the approach suggested by the reviewer. In this sense, the terminology we used is determined by the research design.

We consider the current approach the clearest way to present the analysis in line with the research methods and to maintain internal consistency, particularly for a global audience. For this reason, we have retained the existing structure, which we believe best reflects the study logic.

3. Pembrolizumab example should discuss whether not having orphan and non-orphan indications on the same MA is a barrier in the EU - there will be a commercial decision here

Thank you for this comment. This is an important point for further discussion of the observed discordance in non-orphan marketing authorisations, particularly in relation to products with multiple oncology indications. A discussion addressing this point has been added to the revised manuscript.

**Please check lines [375-387].**

4. New errors are introduced - e.g. This shift is exemplified by the FDA's 2017 tissue-agnostic approval of pembrolizumab for solid tumours with NTRK-fusion proteins - this was not pembrolizumab - please carefully check your text for errors

Thank you for spotting this error. We have corrected this in the revised manuscript.

**Please check lines [214-217].**

5. Limitations - Based on the research goal to understand the discordance between US and EU, a major limitation of this study is not addressing from a research perspective the differences in regulatory ask on significant benefit in the EU, and other regulatory criteria e.g. allowance of subsetting or stage of condition from US perspective which is acceptable for FDA status.

In line with the study’s objective, we have examined US–EU discordance in orphan designation and orphan drug approval outcomes. However, due to data availability constraints, jurisdiction-specific regulatory requirements, such as the EU requirement for significant benefit and the FDA’s approach to orphan subsetting, were not explicitly assessed. This limitation has now been acknowledged in the main text.

**Please check lines [428-430]**

6. Duplication of a reference is noted - 29 and 33

The reference has been corrected, and the subsequent sections have been reordered accordingly.

**Reviewer #2**: Thank you for the thoughtful revisions. They have strengthened the manuscript, while I have just one remaining methodological point.

I agree that many regulatory or policy analyses do not routinely report pseudo-R² or other global fit measures. But some recent work has included such metrics simply to illustrate how much of the variation is explained by the available covariates. For example, Hong et al. (Hong, D., Kesselheim, A.S., Sarpatwari, A. and Rome, B.N., 2024. Biosimilar Uptake In The US: Patient And Prescriber Factors: Article examines biosimilar uptake in the US. Health Affairs, 43(8), pp.1159-1164.) reported the McFadden pseudo-R² to help contextualize the limitations of the model, and they also discussed the contribution of unmeasured factors.

I understand that your analysis does not involve model selection or causal inference. My suggestion is purely about transparency: providing a brief pseudo-R² value in the Supplementary Appendix or notes below the table would help readers understand how much of the EMA decision variation the prespecified variables account for, given that several regulatory/pricing/political framework determinants cannot be directly measured.

Overall, the revisions are strong, and addressing this small point would further clarify the completeness and interpretability of the adjusted associations.

We agree with the third reviewer’s comments on the modelling. While pseudo R² and AIC/BIC are useful for assessing model discrimination and model fit, respectively, they do not directly quantify the strength of association. Odds ratios are therefore the most appropriate effect measure for this analysis. Therefore, we choose to not include pseudo R2 and AIC/BIC in the manuscript.

**Reviewer #3**: Thanks for the opportunity to read your manuscript. My role is statistical reviewer, so I have focused on the design, data, and analysis that are presented. I have put general comments first, followed by questions relevant to a specific section of the manuscript (with a page/line reference).

I agree with the authors about the use of odds ratios over other measures (e.g. pseudo R2 and AIC/BIC). Pseudo R2 is more useful as measure of discrimination of a particular model, rather than an estimate of how a covariate is associated with an outcome. Measures of model fit (AIC/BIC) also are useful to select between models with different sets of covariates but can't directly quantify the strength of association. The odds ratio is an appropriate effect estimate for this data.

We agree with the reviewer. While pseudo R² and AIC/BIC are useful for assessing model discrimination and model fit, respectively, they do not directly quantify the strength of association. Odds ratios are the most appropriate effect measure for this analysis. Therefore, we choose to not include pseudo R2 and AIC/BIC in the manuscript.

There is some material in the results that belongs in the methods, i.e. the link between an analysis and a particular research question (e.g. L303).

Thank you for identifying this misplaced material. We have revised the manuscript to more clearly articulate the link between the analysis and the relevant research question, and have moved this content from the Results section to the Introduction section.

**Please check lines [152-155].**

The data for the study is likely to be non-independent, i.e. clustered at the level of the company. Can you confirm that there is no clustering, or else use a method that appropriately accounts for the clustering (e.g. GEE)?

P7, L221. I wasn't clear about the need for a multivariable logistic regression model. If the analysis is causal, and includes covariates that are potential confounders or mediators, then the proposed causal model needs to be explained to ensure that effects from the model are interpreted correction (i.e. 'table 2 fallacy' https://doi.org/10.1093/aje/kws412). Measures of discrimination (AUC, sens/spec) are presented, but the research question does not seem to be to build a prediction model for the different authorisation/approval outcomes. For a descriptive or causal analysis, measures of discrimination are not particularly useful as they simple quantify the overall level of predictions (concordance) rather than assessing the quality of the model. If the aim is predictive, an assessment of calibration should also be included. If the aim is more descriptive, then multivariable models may not be necessary and a series of univariate regression models for each outcome and covariate would be more appropriate.

We thank the reviewer for these helpful comments.

We agree that observations are likely to be non-independent due to multiple approvals/designations contributed by the same company (814 approvals and 204 companies). We have therefore revised the analytic approach to explicitly account for clustering at the company level. Specifically, we now use logistic regression estimated via Generalized Estimating Equations (GEE) with company as the clustering unit and robust (sandwich) standard errors, providing valid inference under within-company correlation.

We also agree that our study is descriptive and not intended to support causal interpretation, nor is it intended to develop a prediction model. To avoid the “Table 2 fallacy” and misinterpretation of adjusted coefficients, we have clarified the descriptive objective in the Methods and have focused on univariable associations between each variable and each outcome.

In line with the revised aim and the reviewer’s point, we have removed AUC and sensitivity/specificity measures from the manuscript, as these metrics are not informative for a descriptive association analysis. We have updated the Methods section accordingly and revised the Results and S4 Table to reflect the GEE-based univariate logistic regression analyses.

**Please check lines [228-234], [242, 243] and S4 Table.**

P10, L314. It would be clearer to just report the odds ratio rather than the % higher or lower the odds are.

Thanks for the helpful comments. All the odd ratio reports have been revised in the draft.

**Please check lines [278-280], [299-301] [303, 309], [321-326] [347, 350] and abstract.**

Figure S6. This is interesting data - but it is difficult to understand a 3d bar plot. This could be better displayed a time-series plot, with each therapeutic area as a series, or as a panel plot of the time-series with an individual section for each therapeutic area.

Thank you for this suggestion. We have revised S6 Figure by replacing the 3D bar plot with a time-series figure, which improves clarity and interpretability across therapeutic areas.

**Please check lines [612-614].**

**General editorial requests:**
(Note: not all will apply to your paper, but please check each item carefully)

A **✓** indicates that the item has been completed.

**✓**     We ask every co-author listed on the manuscript to fill in a contributing author statement, making sure to declare all competing interests. If any of the co-authors have not filled in the statement, we will remind them to do so when the paper is revised. If all statements are not completed in a timely fashion this could hold up the re-review process. If new competing interests are declared later in the revision process, this may also hold up the submission. Should there be a problem getting one of your co-authors to fill in a statement we will be in contact. Please do not add or remove authors without first discussing this with the handling editor. You can see our competing interests policy here: <http://journals.plos.org/plosmedicine/s/competing-interests>.

**✓**      Please upload any figures associated with your paper as individual TIF or EPS files with 300dpi resolution at resubmission; please read our figure guidelines for more information on our requirements: <http://journals.plos.org/plosmedicine/s/figures>. While revising your submission, we strongly recommend that you use PLOS's NAAS tool (<https://ngplosjournals.pagemajik.ai/artanalysis>) to test your figure files. NAAS can convert your figure files to the TIFF file type and meet basic requirements (such as print size, resolution), or provide you with a report on issues that do not meet our requirements and that NAAS cannot fix.

After uploading your figures to PLOS's NAAS tool - <https://ngplosjournals.pagemajik.ai/artanalysis>, NAAS will process the files provided and display the results in the "Uploaded Files" section of the page as the processing is complete.
If the uploaded figures meet our requirements (or NAAS is able to fix the files to meet our requirements), the figure will be marked as "fixed" above. If NAAS is unable to fix the files, a red "failed" label will appear above.
When NAAS has confirmed that the figure files meet our requirements, please download the file via the download option, and include these NAAS processed figure files when submitting your revised manuscript.


**✓**     Please ensure that the paper adheres to the PLOS Data Availability Policy (see <http://journals.plos.org/plosmedicine/s/data-availability>), which requires that all data underlying the study's findings be provided in a repository or as Supporting Information. For data residing with a third party, authors are required to provide instructions with contact information (web or email address) for obtaining the data. Please note that a study author cannot be the contact person for the data. PLOS journals do not allow statements supported by "data not shown" or "unpublished results." For such statements, authors must provide supporting data or cite public sources that include it.

**✓**      We expect all researchers with submissions to PLOS in which author-generated code underpins the findings in the manuscript to make all author-generated code available without restrictions upon publication of the work. In cases where code is central to the manuscript, we may require the code to be made available as a condition of publication. Authors are responsible for ensuring that the code is reusable and well documented. Please make any custom code available, either as part of your data deposition or as a supplementary file. Please add a sentence to your data availability statement regarding any code used in the study, e.g. "The code used in the analysis is available from Github [URL] and archived in Zenodo [DOI link]" Please review our guidelines at <https://journals.plos.org/plosmedicine/s/materials-software-and-code-sharing> and ensure that your code is shared in a way that follows best practice and facilitates reproducibility and reuse. Because Github depositions can
be readily changed or deleted, we encourage you to make a permanent DOI'd copy (e.g. in Zenodo) and provide the URL.

The data and code used in the analysis are available from the University of Sheffield repository with DOI assigned. The DOI will be provided upon acceptance of the paper.

**✓**     Please ensure that the study is reported according to the STOBE guideline and include the completed checklist as Supporting Information. When completing the checklist, please use section and paragraph numbers, rather than page numbers. Please add the following statement, or similar, to the Methods: "This study is reported as per [XXXX] guideline (S1 Checklist)." **Please check lines [241-242] and S2 Checklist.**

FORMATTING - GENERAL

**✓**  Please use the active voice throughout.
**✓**  Please be consistent in use of verb tenses.
**✓**  Please be concise in presentation of the Results.
**✓**  The Discussion is >2500 words. Please reduce to 1000 words. We have tried our best to reduce from 2500 to 1029 without losing the core arguments and key contributions.
**✓**  Please retain the discussion of the potential health impacts of differences in drug approvals, and whether data are available on this issue.

**✓**     Abstract: Please structure your abstract using the PLOS Medicine headings (Background, Methods and Findings, Conclusions). Please combine the Methods and Findings sections into one section. **Please check lines [18-48].
✓**	In the last sentence of the Abstract Methods and Findings section, please describe the main limitation(s) of the study's methodology. **Please check lines [47-48].
✓**	Please limit the Conclusions section of the Abstract to 4 sentences pertaining to the findings and their implications. This should not reiterate background information. **Please check lines [49-58].**

**✓**       At this stage, we ask that you include a short, non-technical Author Summary of your research to make findings accessible to a wide audience that includes both scientists and non-scientists. The Author Summary should immediately follow the Abstract in your revised manuscript. This text is subject to editorial change and should be distinct from the scientific abstract. Ideally each sub-heading should contain 2-3 single sentence, concise bullet points containing the most salient points from your study. In the final bullet point of 'What Do These Findings Mean?', please include the main limitations of the study in non-technical language. Please see our author guidelines for more information: <https://journals.plos.org/plosmedicine/s/revising-your-manuscript#loc-author-summary>. **Please check lines [59, 83].**

**✓**      Please express the main results with 95% CIs as well as p values. When reporting p values please report as p<0.001 and where higher as the exact p value p=0.002, for example. Throughout, suggest reporting statistical information as follows to improve clarity for the reader "22% (95% CI [13%,28%]; p</=)". Please be sure to define all numerical values at first use.

**✓**       Please include page numbers and line numbers in the manuscript file. Use continuous line numbers (do not restart the numbering on each page).

**✓**      Please cite the reference numbers in square brackets. Citations should precede punctuation.

**✓**	Please revise your title to comply with PLOS Medicine's style. Your title must be nondeclarative and not a question. It should begin with main concept if possible. "Effect of" should be used only if causality can be inferred, i.e., for an RCT. Please place the study design ("A randomized controlled trial," "A retrospective study," "A modelling study," etc.) in the subtitle (ie, after a colon).                                                                                                                                                                                                                                                                                                                                                                                                         
**✓** Please ensure that the Introduction ends with a clear description of the study question or hypothesis.                                                                                                                                                                                                       
**✓** Please ensure that all abbreviations are defined at first use throughout the text.                                                                                                                                                                                                   
**✓** Please confirm that all numbers presented in the abstract are present and identical to numbers presented in the main manuscript text.
**✓** Please remove all subheadings from the Discussion.                                                                                                                                                                                           
**✓** Please add URLs for funders. **Please check lines 461.**

FIGURES AND TABLES
**✓**       Please provide titles and legends for all figures and tables (including those in Supporting Information files).

**✓**      Please define all abbreviations used in each figure/table (including those in Supporting Information files).

**✓**      Please consider avoiding the use of red and green in order to make your figure more accessible to those with color blindness.

SUPPLEMENTARY MATERIAL
**✓**       Please note that supplementary material will be posted as supplied by the authors. Therefore, please amend it according to the relevant comments outlined here.

**✓**      Please cite your Supporting Information as outlined here: <https://journals.plos.org/plosmedicine/s/supporting-information>

REFERENCES
**✓**       PLOS uses the numbered citation (citation-sequence) method and first six authors, et al.

**✓**      Please ensure that journal name abbreviations match those found in the National Center for Biotechnology Information (NCBI) databases (<http://www.ncbi.nlm.nih.gov/nlmcatalog/journals>), and are appropriately formatted and capitalised.

**✓**       Where website addresses are cited, please include the complete URL and specify the date of access (e.g. [accessed: 12/06/2023]).

**✓**      Please also see <https://journals.plos.org/plosmedicine/s/submission-guidelines#loc-references> for further details on reference formatting.


OBSERVATIONAL STUDIES

**✓**       Abstract: Please include the study design, population and setting, number of participants, years during which the study took place (enrollment and follow up), length of follow up, and main outcome measures.

**✓**       Please ensure that the study is reported according to the STROBE (or appropriate STOBE extension) guideline (available from: <https://www.equator-network.org/reporting-guidelines/strobe>) and include the completed STROBE (or STROBE extension) checklist as Supporting Information. Please add the following statement, or similar, to the Methods: "This study is reported as per the Strengthening the Reporting of Observational Studies in Epidemiology (STROBE) guideline (S1 Checklist)." When completing the checklist, please use section and paragraph numbers, rather than page numbers.

**✓**      For all observational studies, in the manuscript text, please indicate: (1) the specific hypotheses you intended to test, (2) the analytical methods by which you planned to test them, (3) the analyses you actually performed, and (4) when reported analyses differ from those that were planned, transparent explanations for differences that affect the reliability of the study's results. If a reported analysis was performed based on an interesting but unanticipated pattern in the data, please be clear that the analysis was data driven.

**✓**      Please state in the Methods section whether the study had a prospective protocol or analysis plan. If a prospective analysis plan (from your funding proposal, IRB or other ethics committee submission, study protocol, or other planning document written before analyzing the data) was used in designing the study, please include the relevant document(s) with your revised manuscript as a Supporting Information file to be published alongside your study and cite it in the Methods section. A legend for this file should be included at the end of your manuscript. If no such document exists, please make sure that the Methods section transparently describes when analyses were planned, and when/why any data-driven changes to analyses took place. Changes in the analysis, including those made in response to peer review comments, should be identified as such in the Methods section of the paper, with rationale.
