# Supplementary material for: Discordance in orphan drug approvals between the U.S. Food and Drug Administration and the European Medicines Agency: A retrospective observational analysis
Source: PLoS Med. 2026 Jul 6;23(7):e1004861. doi: 10.1371/journal.pmed.1004861 (PMC13375132; doi:10.1371/journal.pmed.1004861)
Supplement: Attachment — Submitted filename: Martin_2026.docx [file pmed.1004861.s011.docx]

**Discordance of Orphan Drug Approvals by the U.S. Food and Drug Administration and the European Medicines Agency: A Retrospective Analysis**

Authors:

Jin Ding (0000-0003-3138-1064)^1 2^, Michael M. Hopkins ([0000-0002-8401-6573](https://orcid.org/0000-0002-8401-6573))^3 4^, Paul Martin (0000-0002-5166-9658)^1*^

Address:

^1^ iHuman, School of Sociological Studies, Politics and International Relations, The University of Sheffield, Sheffield, United Kingdom

^2^ Department of Management, University of Exeter, Exeter, United Kingdom

^3^ Science Policy Research Unit, Business School, University of Sussex, Brighton, United Kingdom

^4^ Centre for Law, Medicine and the Life Sciences, Faculty of Law, University of Cambridge, United Kingdom

**^*^** Corresponding author

Email address: [paul.martin@sheffield.ac.uk](mailto:paul.martin@sheffield.ac.uk) (PM)

# Abstract

**Background**

The United States (US) and European Union (EU) have long-established orphan drug regulations to incentivize the development of medicines for rare diseases. Whilst the numbers of orphan drug approvals have risen rapidly, there is increasing discordance in regulatory outcomes between the US and EU. This discordance is primarily comprised of two groups: FDA orphan approvals not authorised by the EMA and those authorised by the EMA without orphan designation. We examined factors associated with these two forms of regulatory discordance.

**Methods and Findings**

We collected data on US Food and Drug Administration (FDA) orphan drug approvals between 2011-2023 and their corresponding European Medicines Agency (EMA) regulatory status. We used descriptive statistical analysis to examine trends and discordance in outcomes between agencies. Univariable logistic regression assessed pre-specified factors associated with discordance, including therapeutic area (cancer/non-cancer), company size (large/medium/small), company headquarters location (US/EU/others) and approval period (2011-2016/2017-2023).

Of 814 FDA orphan approvals, only 29% received corresponding EMA marketing authorisation with orphan designation. A further 38% were authorised by EMA without orphan status, and the remaining 33% were not authorised by EMA. Compared with the earlier, 2011-2016 period, the later, 2017-2023 period was associated with lower odds (OR 0.66, 95% CI 0.48-0.92; p=0.013) of marketing authorization by the EMA. For FDA orphan drug approvals that were not authorized by the EMA, sponsors opted not to pursue EMA review largely for commercial reasons (75% of cases). Compared with cancer drugs, non-cancer drugs were associated with lower odds (OR 0.53, 95% CI 0.38-0.75; p<0.001) of having EMA marketing authorisation, but when authorised, were associated with higher odds (OR 2.36, 95% CI 1.50-3.70; p<0.001) of receiving orphan designation. Compared with large companies, orphan approvals from small and medium-sized companies had lower odds ((OR 0.45, 0.28–0.74; p=0.001); (OR 0.29, 0.20–0.43; p<0.001)) of EMA marketing authorisation, but among authorised products they had higher odds (OR 2.95, 95% CI 1.75–4.99; p<0.001); (OR 2.14, 95% CI 1.17–3.90; p=0.014)) of orphan-designated marketing authorisation, respectively. EU companies were associated with higher odds (OR 1.69, 95% CI 1.17–2.43; p=0.005) of receiving EMA orphan approvals compared with US companies. Findings were limited to comparisons between the FDA and EMA on orphan drugs and may not be generalised to non-orphan drugs.

**Conclusions**

Between 2011-2023, regulatory outcomes for orphan drug approvals increasingly diverged between the FDA and EMA. Most FDA-approved orphan drugs that lack EU marketing authorisation appear to be withheld by sponsors not because of regulatory barriers but due to insufficient commercial incentives to launch in Europe, resulting in fewer treatment options for European rare disease patients. Among FDA orphan drugs authorised by the EMA, many were not designated as orphan products because of regulatory differences, particularly regarding requirements around significant benefit and biomarker-defined sub-populations in oncology. Our findings suggest that orphan incentives are not the primary driver of commercial EU-launch decisions and that recent and proposed EU regulatory reform of these incentives may not achieve their goal of improving access to therapy for rare diseases.

**Author summary**

### **Why was this study done?**

- Although both the US Food and Drug Administration (FDA) and the European Medicines Agency (EMA) have approved increasing numbers of orphan drugs over time, there is a growing discordance between outcomes for the same indication between the two regions that these regulators serve.
- It remains poorly understood whether this gap is associated with product-level characteristics or company decisions.

### **What did the researchers do and find?**

- We performed a comprehensive analysis of discordance at the level of indication approval between the FDA and EMA across all therapeutic areas and through the lens of commercial sponsors.
- Only 29% of FDA-approved orphan drug approvals were similarly designated and authorised by the EMA. In contrast, a larger proportion of drugs, were either authorised without orphan status (38%) or lacked marketing authorization by EMA (33%).
- The high number of cases where FDA orphan approvals did not have a corresponding EMA marketing authorisation for the relevant indication are due to company sponsors, particularly small, US-based companies, not seeking market authorisation in Europe for commercial reasons.

### **What do these findings mean?**

- These findings can inform policy makers seeking to stimulate innovation and improve patient access to important treatments.
- The findings question the likely effectiveness of policies based on orphan market exclusivity to influence company sponsor behaviour. Instead, other issues beyond the regulator’s influence such as more stringent health technology assessment, pricing and reimbursement policies in individual EU nations may play an important role in shaping companies’ decisions.

# 1 Introduction

Orphan drugs have become a major focus for drug developers and now account for half of all new drugs approved each year by the United States (US) Food and Drug Administration (FDA) [1]. Yet substantial differences have emerged in the availability of these drugs across jurisdictions. This raises important policy questions about patient access to therapy, particularly in light of recent proposals for regulatory change in the European Union (EU) [2]. The US Orphan Drug Act (ODA) was enacted in 1983 to encourage the availability of drugs for rare diseases by incentivising the development of medicines for markets that might otherwise be commercially unattractive. Incentives provided by the ODA include a seven-year period of market exclusivity, tax credits, research grants, and regulatory assistance [3]. Incentives only apply to drugs receiving an orphan drug designation from the FDA, with such designations only available for medicines with patient populations of less than 200,000 in the US. The ODA has been credited with stimulating an increased level of innovation [4,5]. In assessing such activity, the term 'orphan approvals’ refers to FDA drug approvals or EMA marketing authorisations for orphan designated indications [4].

The EU adopted its own orphan legislation in 2000, however, the rate of orphan approvals by the European Medicines Agency (EMA) is much lower compared to the FDA [6]. The EMA has also been reported as slower than the FDA in granting orphan approvals [7]. Comparative research focused on specific therapeutic areas, such as oncology, and product types, such as Advanced Therapy Medicinal products (ATMPs) shows notable differences. The FDA has approved more oncology drugs with orphan designation than the EMA [8], while the EMA authorised more ATMPs with orphan designation than the FDA [9]. The factors associated with the discordance between the FDA and EMA in terms of orphan approvals remain poorly understood. Here we use a step-wise analysis to explore the association between pre-specified variables and the apparent trends.

Firstly, regulatory differences can result in different outcomes for the same drug across jurisdictions [9–12]. In a comparison of cancer drug applications reviewed by the FDA and EMA, there was frequent discordance between these agencies in approvals and use of expedited programs [13]. Secondly, the responses of various types of companies to the same choice of market opportunity may play a role as there are major differences in the behaviour, strategies, and capabilities of different sized companies in the pharmaceutical industry. Large pharmaceutical companies have tended to dominate the development and marketing of medicines in particular therapeutic areas, while small and medium-sized enterprises (SMEs) involved in Research and Development (R&D) may have more limited experience and resources to bring products to market. Yet the division of labour in drug development between large and smaller companies is also shifting rapidly, with smaller companies playing a greater role over time [14]. However, smaller companies tend to target areas with lower returns, whereas larger companies focus on higher-return opportunities [15]. Larger companies might be expected to more readily deal with complex regulatory processes due to their greater financial resilience, experience, resources, and established credibility. Large pharmaceutical companies are able to absorb delays, navigate regulatory complexity, and adapt product development following regulatory feedback [16]. Independently of company size, headquarters location may also contribute to the observed gap between the FDA and EMA approval rates. Both the FDA and EMA issued more approvals to companies headquartered in their own regions and fewer to firms based elsewhere [17].

International comparative analysis of orphan drug approvals must account for the tendency of drugs to be approved for different numbers of indications at the FDA and EMA. For this reason, we draw an important distinction between an orphan drug (a specific molecule with at least one orphan designation) and an orphan drug approval, which refers to the drug approval for orphan designated indications. Both the FDA and EMA allow drugs under development to be granted orphan designation prior to approval for marketing (we use the terms ‘marketing authorisation’ and ‘approval’ synonymously). Previous studies comparing orphan drugs between the FDA and EMA have often overlooked the important distinction raised by multiple indications. Ignoring this distinction could result in incomplete assessments of the orphan drug landscape. Our study places emphasis on examining approved drug indications to investigate the evolving differences in rates of approval between the two agencies. The importance of looking at the pattern of approved indications (in contrast to orphan drugs) is illustrated in Fig 1, which shows the multiple approvals of pembrolizumab (Brand name: Keytruda) over time at the FDA and EMA. Pembrolizumab was approved for nine indications in the period studied. No other drug had more approved indications. Despite its commercial focus on orphan indications, pembrolizumab was ranked as the top pharmaceutical product by global sales in 2023 and 2024. Pembrolizumab provides a natural experiment illustrating how differences arise between rates of orphan approvals at the FDA and EMA, with the drug being repeatedly granted orphan authorisation in the US while obtaining fewer marketing authorisations from the EMA, and no orphan designation for any indication.

**Fig 1. Orphan designations and approvals of pembrolizumab (brand name: Keytruda) by the United States Food and Drug Administration (FDA) and the European Medicines Agency (EMA), 2011–2023. The figure shows that pembrolizumab received FDA approval for 14 orphan-designated indications during this period, whereas the EMA authorised 11 indications (none with orphan designation) over the same timeframe.**

In this paper we explore an important gap in knowledge by mapping the divergent pattern of orphan designations and approvals between the FDA and EMA for all therapeutic areas between 2011-2023; We also explore how a number of variables (therapeutic focus, size of company sponsor, headquarters location, and approval year) may be associated with the observed differences. The paper addresses two research questions: firstly, which factors were associated with non-orphan EMA marketing authorisations; and second, which factors were associated with the absence of EMA marketing authorization. The implications of our results are discussed in the context of recent policy proposals in the EU to improve patient access to treatments for rare diseases [2].

# 2 Methods

## 2.1 Study cohort

We provide analysis of a retrospective cohort of orphan approvals granted by the FDA between 1 January 2011 to 31 December 2023, to determine their EMA regulatory status. Key information extracted from the publicly accessible dataset of the FDA ([Orphan Drug Designations and Approvals](https://www.accessdata.fda.gov/scripts/opdlisting/oopd/)) include generic and brand name, indication, sponsor, date of orphan designation, and approval date. The FDA-approved indications which had been withdrawn and approved indications with designations withdrawn or revoked were excluded from the dataset. To determine whether the FDA orphan approvals were designated and authorised by the EMA during the same period, we then search the [Union Register of medicinal products](https://ec.europa.eu/health/documents/community-register/html/) dataset and European Public Assessment Reports (EPARs) to check the related orphan designation and marketing authorisations. The withdrawn EMA market authorisation applications were cross-checked with Committee for Medicinal Products for Human Use (CHMP) reports. We only analyse drugs that received centralised approval by the EMA. We do not assess drugs approved through national, decentralised, or mutual recognition procedures, as these were outside the scope of our research. This focus is justified by the requirement that drugs with an orphan designation must undergo the centralised procedure, and the fact that the majority of new, innovative medicines were evaluated by the EMA through this process [18].

The FDA and EMA follow distinct regulatory pathways for orphan drugs. In the United States, the FDA’s Office of Orphan Products Development grants orphan designation and marketing approval through separate but closely linked processes. In contrast, the EMA applies a two-step framework: the Committee for Orphan Medicinal Products (COMP) grants orphan designation prior to the marketing authorisation application and re-evaluates eligibility at the time of marketing authorisation. Orphan designation may be withdrawn either during this reassessment or post-authorisation if the criteria were no longer met. This sequential process underpins our two-stage modelling framework, in which Stage 1 estimates the likelihood of any EMA marketing authorisation (orphan and non-orphan) and Stage 2, conditional on EMA marketing authorisation, estimates the likelihood of being orphan designated. Based on that, the EMA regulatory status is classified into three categories based on whether they were designated and authorised by the EMA, or not (Table 1).

**Table 1. Categories of European Medicines Agency (EMA) regulatory status applied to United States Food and Drug Administration (FDA) orphan approvals from 2011 to 2023.**

| EMA Status | Explanation |
| --- | --- |
| Orphan Marketing Authorisation | FDA orphan approvals that have also been authorised by the EMA for an orphan designated indication by the end of 2023. This includes products with active orphan status during the period from 2011 to 2023 that had expired before 2024. |
| Non-orphan marketing authorisation | FDA orphan approvals that have been authorised for marketing by the EMA without orphan designation, or with orphan designations withdrawn before marketing authorisation, at the time of marketing authorisation, or after marketing authorisation by the end of 2023. |
| Not Authorised | FDA orphan approvals that did not have marketing authorisation by the EMA before the end of 2023, for the following reasons: 1) an application for marketing authorisation was not submitted to EMA, 2) an application was withdrawn by the sponsor during marketing authorisation application, 3) the application was refused, or 4) the marketing authorisation was granted but withdrawn later. |

Prior research observed a delay in EMA orphan marketing authorisations, relative to the FDA, with a median time lag for designation of three months and median time lag of six months for marketing authorisation [6]. Our results primarily focused on the EMA regulatory status of the study cohort up to the end of 2023. We compared FDA and EMA regulatory processes and outcomes within the same time window to permit the most direct, like-for-like comparison feasible from regulatory disclosures.

In order to maintain cross-agency symmetry and minimise misclassification of time-varying regulatory states, we excluded FDA approvals that had been withdrawn or revoked. This exclusion may introduce selection bias by under-representing products with complex approval histories (e.g., safety-related withdrawals or strategic discontinuations), potentially biasing estimates toward more durable approvals. Nonetheless, these cases represented a very small proportion (<2%) of the dataset.

## 2.2 Therapeutic areas, company size, HQ location and approval period

The study aimed to characterise cross-agency alignment and identify company- and product-level factors associated with EMA outcomes. The analysis was conducted at the indication level. Covariates were pre-specified based on prior literature examining determinants of regulatory approval and orphan drug development, including therapeutic area, sponsor size, headquarters location, and period.

Our analysis of the therapeutic area of each approval was based on the World Health Organization’s Anatomical Therapeutic Chemical (ATC) Index 2022. We further grouped the therapeutic areas into cancer (ATC L01–L03) versus non-cancer (L04 and all other ATC groups) [19]. We categorised the size of sponsors into three groups based on 2023 annual pharmaceutical sales from [Scrip100](https://invivo.pharmaintelligence.informa.com/outlook/scrip-100): Tier 1 (large, n=22; >$10B in annual pharmaceutical sales), Tier 2 (medium, n=27; $1–$10B), and Tier 3 (small, n=156; <$1B) (S1 Table). We used Citeline’s Pharmaprojects [20] database to determine for each drug the sponsor and its headquarters (HQ) country in 2023. We pre-specified approval year indicator (2011–2016 vs 2017–2023) to capture the regulatory and scientific shifts, including in 2016 the launch of the EMA’s PRIME scheme [21] and updates to orphan designation guidance [22] which tightened the interpretation of “significant benefit” and imposed stricter conditions on orphan designation for biomarker-defined subsets. In parallel, US legislation and regulatory initiatives - the 21st Century Act [23] in 2016 and Orphan Drug Modernization Plan [24] in 2017 - promoted the growing prominence of biomarker-driven development [25–27]. The FDA’s 2017 tissue/site-agnostic approval of pembrolizumab for unresectable or metastatic MSI-H or mismatch repair-deficient solid tumours, exemplified this shift, representing the first approval based on a genomic biomarker across tumour types [28], while there had been virtually no recent EMA orphan designations successfully based on biomarker subsetting [29].

## 2.3 Statistical analysis

Descriptive statistical analysis summarised FDA regulatory outcomes by therapeutic area, sponsor size, headquarters location, and approval period. The regulatory outcomes of FDA orphan approvals at the EMA were analysed to examine the influence of these variables on regulatory outcome. This analysis aimed (1) to quantify the contribution of each variable to differences in EMA regulatory status, and (2) to assess whether these variables were associated with the probability of different regulatory outcomes.

To investigate the growing discordance in approval outcomes between the FDA and EMA, we implemented a pre-specified two-stage modelling framework mirroring the sequential EMA decision process for FDA orphan approvals. We used a univariable logistic regression model to examine the association between the outcome and each explanatory variable individually. This approach provided a transparent, descriptive assessment of bivariate relationships rather than constructing a causal or predictive model. Because multiple EMA marketing authorisations were attributed to the same company (814 approvals developed by 204 companies), observations are not statistically independent. We therefore used logistic regression models fitted with generalized estimating equations (GEE) to account for within-company clustering. Stage 1 used univariable logistic regression fitted with GEE to examine the association between pre-specified variables and the likelihood of any EMA marketing authorisation. Stage 2, restricted to EMA authorised FDA orphan approvals, assessed the associations between these variables and the likelihood of orphan versus non-orphan authorisation. Both models included pre-specified variables - period (2011–2016 vs 2017–2023), therapeutic area (cancer vs non-cancer), company size (large, medium, small), and company location (EU, US, Other).

This study was reported as per the Strengthening the Reporting of Observational Studies in Epidemiology (STROBE) guideline (S2 Checklist). All analyses are conducted in R (version 4.4.3). Estimates were from univariable GEE logistic regression models accounting for clustering at the company level. We considered two-tailed p values <0.05 statistically significant and reported 95% confidence intervals (CI) throughout. We presented detailed data and regression results in S3 Table and S4 Table.

# 3 Results

Between 2011 and 2023 the FDA granted 814 orphan approvals. Fig 2 shows the corresponding status for this cohort at the EMA. By the end of 2023, 235 (29%) of these cases were designated and authorised for orphan indications by the EMA. A further 308 (38%) of the FDA orphan approvals were authorised by the EMA without orphan designation, while the remaining 271 (33%) were not authorised by the EMA for the relevant indication. The number of annual FDA orphan approvals increased approximately three-fold during the studied decade, from 26 in 2011 to 91 in 2023. In the first period an annual mean of 11 FDA orphan approvals wase designated and authorised by the EMA, with this increasing to an annual mean of 24 in the second period. However, cases of FDA orphan approval not being authorised by EMA rose more quickly from an annual mean of 11 between 2011-2016, to 29 annually between 2017-2023. The net effect of these differences is that whilst 543 (67%) of FDA orphan approvals were also granted marketing authorisation by the EMA, a large proportion of these obtained authorisation without orphan designation.

**Fig 2. Cumulative number of United States (US) orphan drug approvals by European Medicines Agency (EMA) regulatory outcome, 2011–2023. X-axis: year of approval by the United States Food and Drug Administration (FDA). Y-axis: cumulative number of products in each EMA regulatory category by the end of 2023.**

For the FDA orphan approvals not authorised in the EU, we find that only 9 (3%) of these cases were due to the refusal by the EMA, while 11 (4%) were due to the withdrawal of the marketing authorisation application, 17 (8%) were withdrawn from use and 2 (1%) were suspended. Analysis of years subsequent to the study period found that the lag in EMA market authorisations (relative to FDA) accounted for a further 25 marketing authorisations granted in 2024 and 5 by September in 2025. This leaves 75% (202/271) of the FDA-EMA gap in approvals unaccounted for. The elimination of other explanations (i.e. the refusals, withdrawals, and lagged marketing authorisations above), leads us to conclude that most FDA orphan approvals were not authorised by EMA because they were never submitted to the agency by sponsoring companies for the relevant indication. In other words, companies may not seek marketing authorisation for many drug indications in Europe, possibly for commercial. Below we explore whether company size and location are associated with these decisions.

**3.1 Approval period**

During the study period, FDA orphan approvals increased, and the EMA outcomes reflected in this expansion. As shown in S5 Figure, absolute FDA orphan approval numbers in 2017–2023 were roughly two- to three-fold more than those in 2011–2016. The discordance with EMA had been growing, as FDA orphan approvals in the later period (2017–2023) had lower odds (OR 0.66, 95% CI 0.48-0.92; p=0.013) of receiving EMA marketing authorisation compared with 2011–2016. However, the ratio of orphan marketing authorisations and non-orphan marketing authorisations appears stable across the two periods, with no significant association between the approval period and the EMA orphan designations.

**3.2 Therapeutic focus**

Differences in rates of authorization or designation by therapeutic area may be a further important variable for the FDA/EMA approval gap. Antineoplastic agents for the treatment of cancer accounted for 43% of all orphan approvals in the US from 2011 to 2023. Neurologic and autoimmune indications accounted for 17% of orphan approvals and other therapeutic areas each contributed less than 15% of FDA orphan approvals. It is notable that the growth in the FDA orphan cancer approvals showed a strong increase between 2011 and 2023 (Figure A in S6 Appendix), from mean annual number of 16 in the first period to 37 in the second period.

**Fig 3. The European Medicines Agency (EMA) regulatory status of United States Food and Drug Administration (FDA) orphan approvals across therapeutic areas.**

Analysis of the EMA outcomes by therapeutic area was undertaken (Fig 3). The most prominent finding is that half of the FDA orphan approvals for cancers were granted marketing authorisation by EMA without orphan designation (50%; 178/353). About one quarter (25%; 89/353) of the total were authorized with orphan designation and another quarter (24%; 86/353) had no EMA marketing authorisation. Compared with cancer drugs, non-cancer drugs were associated with lower odds (OR 0.53, 95% CI 0.38-0.75; p<0.001) of having EMA marketing authorisation, but higher odds (OR 2.36, 95% CI 1.50-3.70; p<0.001) of being authorised with orphan designations.

In the non-cancer therapeutic areas, the Sensory and others category exhibited the highest proportion of non-authorized FDA approvals (53%), followed by Neurologic and autoimmune (46%). These two therapeutic areas were associated with lower odds (Sensory and others (OD 0.38, 95% CI 0.24-0.61; P<0.001); Neurologic and autoimmune (OD 0.34, 95% CI 0.21-0.57; P<0.001)) of having EMA marketing authorization compared with approvals for cancer treatments (Table B in S6 Appendix). In contrast, the area of Alimentary and metabolism diseases had the highest proportion (67%) of orphan marketing authorisations granted by EMA, and it was associated with higher odds (OD 3.25, 95% CI 1.53-6.90; P=0.002) of being authorised with orphan designation.

### **3.4 Size of the sponsor companies**

The large (Tier 1) companies dominated the rare disease market, contributing 56% of FDA orphan approvals. Medium (Tier 2) companies accounted for a further 14%, while the remaining 30% of FDA orphan approvals were held by relatively small (Tier 3) companies. Figure 4 summarises the results.

**Fig 4. Distribution of European Medicines Agency (EMA) regulatory outcomes by sponsor company size, showing the absolute number and proportion of approvals across large, medium, and small companies.**

Among large sponsors, EMA marketing authorisations without orphan designation were nearly twice as numerous as those orphan-designated (233:123). In contrast, medium sized companies were the most likely to gain EMA orphan marketing authorisation (41/116, 35%) while small companies were most likely to not have EMA marketing authorisation (128/243, 53%)**.** Compared with large sponsors, small sponsors (OR 0.45, 0.28–0.74; p=0.001) and medium sponsors (OR 0.29, 0.20–0.43; p<0.001) were associated with lower odds of obtaining EMA marketing authorisation. Conversely, small sponsors (OR 2.95, 95% CI 1.75–4.99; p<0.001) and medium sponsors (OR 2.14, 95% CI 1.17–3.90; p=0.014) were associated with greater odds of obtaining EMA marketing authorisation with orphan designation. Further examination of company size across therapeutic areas showed that large companies accounted for the majority of FDA orphan approvals in cancer (75%; 263/353). However, more than half of these FDA orphan cancer approvals sponsored by large companies (57%; 149/263) were authorised by the EMA without receiving orphan designation. In contrast, nearly half of FDA orphan approvals for cancer indications sponsored by small companies (43%; 28/65) were not authorised by the EMA.

**3.5 HQ location of sponsor companies**

**Fig 5. Distribution of European Medicines Agency (EMA) regulatory outcomes by sponsor headquarters (HQ) location.**

The international distribution of the pharmaceutical industry was also relevant to the gap in FDA/EMA orphan approvals, and provides further context for the approval trends observed by size of sponsor. As shown in Fig 5, the vast majority of orphan approvals at the FDA and EMA were accorded to companies headquartered in the US or Europe. Sponsors from the US contributed to 57% of FDA orphan approvals, while sponsors from Europe accounted for 35%. Companies from other countries, including Japan, Israel, Canada, Australia, China, and India, sponsored only 8% of FDA orphan approvals.

When considering the size of these firms, the composition of the US and European companies could be seen to differ in a consequential manner. Whilst the US and Europe had a similar number of large companies (9 vs 8 respectively) and medium companies (9 US and 12 Europe), most small companies (109) were based in the US, compared to just 38 based in Europe. We therefore examined the influence of the sponsor’s HQ geographical location on approval rates.

Of the 461 FDA orphan approvals in the cohort sponsored by US companies 37% (172/461) were not authorised by the EMA, while the proportion was 26% for European companies. Compared with products developed by US companies, those from Europe-based companies showed higher odds of receiving EMA marketing authorisation (OR 1.69, 95% CI 1.17–2.43; p=0.005), whereas no statistically significant difference was observed for companies headquartered in other regions. Moreover, a substantial proportion (60%; 103/172) of FDA orphan approvals sponsored by US companies that were not authorised by the EMA were from small US companies. Where European sponsors had FDA orphan approvals, these were more likely (31%; 74/285) to have a corresponding EMA marketing authorisation than US companies (27%; 124/461); but this difference was not statistically significant.

# 4 Discussion

Comparison of the rates of orphan approval by the FDA and EMA between 2011-2023 revealed two key trends. Firstly, while both regulators have increased annual orphan approvals, there is growing discordance between them in the observed outcomes for specific indications. Overall, only 29% of FDA orphan approvals had corresponding orphan marketing authorisation from the EMA. Secondly, a large and increasing number of FDA orphan approvals were being granted marketing authorisation by EMA without a corresponding orphan designation (38%) or were not authorised for that indication (33%).

In assessing which factors were associated with non-orphan EMA marketing authorization, our results show that rare cancer drugs were less likely to receive EMA authorisation with orphan designation than drugs in non-cancer therapeutic areas. As precision oncology has become increasingly dependent on biomarkers and the analysis of mutations, differences in regulatory policy governing biomarker use has assumed an increasingly important explanatory role in observed discordance [30, 31]. Vokinger & Kesselheim found that when comparing orphan drug designations for cancer treatments in the US and EU, the FDA approved more cancer drugs with orphan designation for biomarker targeted subgroups of prevalent cancers than the EMA [8]. A regulatory difference of growing importance seems to be FDA’s much greater acceptance in orphan designation of biomarker-defined rare subsets of more common conditions [32, 33]. In contrast, the EMA has more stringent criteria to designate drugs for biomarker-based subsets; one being the ‘plausible link to the broader sub-set condition’ and another is the ‘exclusion of effects outside the subset’ [29, 34].

Our results also show that large companies were less likely to obtain marketing authorisations with orphan designation, suggesting that large firms rely less on orphan incentives when entering the EU market. This implies that the 10-year period of market exclusivity already provided may not be the main determinant of company behavior, especially large companies, when choosing where to launch an orphan product. Firstly, companies may be more dependent on patent protection to avoid competition as only a small proportion of orphan drugs had regulatory exclusivity extending beyond their patent protection [35]. This is because the advantages of orphan incentives may be outweighed by the pursuit of extended non-orphan indications, and the expansion of marketing authorisation across multiple geographic markets [36]. Large companies are particularly well positioned to capitalise on these strategies, given their greater financial resources, global clinical trial capacity, and experience in managing multi-indication and multi-jurisdictional regulatory portfolios [37, 38]. Secondly, exclusivity offers legal protection, not a guarantee of commercial return, which also depends on pricing and reimbursement decisions, HTA outcomes, market size, and distribution/access constraints.

In understanding which factors were associated with the absence of EMA marketing authorization, our findings highlight the importance of therapeutic area, company size and HQ location. Cancer drugs are more likely to be authorised by the EMA than non-cancer drugs. This reflects regulatory prioritisation of oncology, including access to expedited pathways, and stronger commercial incentives linked to high prices [39, 40]. Commercial behaviour, particularly associated with company size, appears to be important in explaining observed discordance in marketing authorizations. Our analysis shows that small companies in general, and US small companies in particular, were less likely to submit for marketing authorisation in Europe when they had a corresponding FDA orphan approval. Yet these companies contribute a growing share of new drugs over time [14]. An explanation may be that small companies are at a competitive disadvantage compared to large companies in terms of resources and expertise [41]. Another important reason for companies withholding their products in Europe is the combination of complexity around reimbursement and relatively lower expected returns. Previous research has found that ‘government-imposed price controls’ were one of the main causes of pharmaceutical innovation lag between the US and the EU, with price and reimbursement regulations delaying the adoption of new pharmaceutical products [42, 43]. Furthermore, the fragmented nature of the European healthcare system imposes relatively higher transaction costs on market entry, making it commercially unattractive for products targeting very small patient populations.

Our findings raise important policy questions about orphan product regulation in the US and EU. How effective is EU regulatory policy in incentivising the launch of drugs for rare diseases and are European patients missing out on treatments available in the US? The challenge for EMA regulation is to ensure the availability of new medicines for rare diseases using targeted regulatory incentives. Policy in the study period 2011-23 was successful in ensuring that the majority (67%) of US orphan approvals were launched in Europe. However, differences in availability are not solely due to European regulatory policy and may also arise from FDA approving a greater number of orphan products, many on the basis of less certain evidence [44]. With 33% of FDA orphan approvals not authorised for corresponding indications in Europe, further research is required to quantify the extent to which European patients lack access to orphan medicines with high therapeutic value available elsewhere. Our findings also cast doubt on the likely effectiveness of recently published reforms of EU pharmaceutical legislation. Proposed changes seek to improve access to drugs for rare diseases by creating a modular form of regulatory exclusivity based on unmet need and therapeutic impact, replacing the current one-size-fits-all incentive [45]. However, our findings suggest that orphan incentives are not the main determinant of company behaviour in launching new products in the EU and may fail to improve access or address the growing discordance with the US, particularly where commercial decisions are based on demand that is mediated by HTA and reimbursement decisions in the national markets that the EMA serves.

The study has a number of limitations. Firstly, we focus on FDA orphan drug approvals and their regulatory status under the EMA, exploring the factors contributing to the increasing discordance. We did not report on the reverse scenario, due to less observed disparity and much smaller numbers (S7 Text). Secondly, due to data availability, this study does not explicitly examine jurisdiction-specific regulatory criteria such as the EU requirement to demonstrate significant benefit or the FDA’s acceptance of sub-setting or stage-specific conditions. Finally, our findings apply specifically to orphan medicines and should not be generalized to treatments for common diseases.

# Supporting information

S1 Table. Large and Medium Companies by Pharma Sales in 2023.

(DOCX)

S2 Appendix. Strengthening the Reporting of Observational Studies in Epidemiology (STROBE) Checklist.

(PDF)

S3 Table. Characteristics of United States Food and Drug Administration (FDA) orphan approvals, 2011–2023. Values are numbers (percentages).

(PDF)

S4 Table. Multivariable logistic regression analysis of the European Medicines Agency (EMA) regulatory status of United States (US) orphan approvals, 2011–2023. Values are odds ratios (95% confidence intervals).

(PDF)

S5 Figure. Orphan drug approvals by the United States Food and Drug Administration (FDA) and Corresponding European Medicines Agency (EMA) regulatory outcomes, 2011–2016 and 2017–2023.

(PDF)

S6 Appendix. Therapeutic areas of United States Food and Drug Administration (FDA) orphan drug approvals and related European Medicines Agency (EMA) regulatory outcomes, 2011-2023.

(PDF)

S7 Text. The study on the Food and Drug Administration (FDA) regulatory status of European Medicines Agency (EMA) orphan marketing authorisations from 2011-2023

(PDF)

# Acknowledgements

We thank Sarah Wallman (The Danish Center for Social Science Research), Jarno Hoekman (University of Utrecht), Wouter Boon (University of Utrecht), and John Abraham (Brighton and Sussex Medical School), and Kathy Liddell (University of Cambridge) for providing their invaluable insights on previous drafts of this article. The views expressed and any remaining errors are those of the authors. We gratefully acknowledge the Wellcome Trust for funding this research [219875/Z/19/Z].

**Financial disclosure**

This study was supported by the Wellcome Trust (219875/Z/19/Z to PM; <https://wellcome.org>), and MH was also supported, in part, by a Novo Nordisk Foundation Grant for a scientifically independent International Collaborative Bioscience Innovation & Law Programme (Inter-CeBIL programme - grant no. NNF23SA0087056); no support from any organisation for the submitted work other than salaries from their employing institutions and the funding listed in the section above; no financial relationships with any organisations that might have an interest in the submitted work in the previous three years; and no other relationships or activities that could appear to have influenced the submitted work.

# References

1	Mullard A. 2020 FDA drug approvals. *Nat Rev Drug Discov* 2021;**20**:85–90.

2	European Parliament. Union procedures for the authorisation and supervision of medicinal products for human use and rules governing the European Medicines Agency. 2024. https://www.europarl.europa.eu/doceo/document/TA-9-2024-0221_EN.pdf

3	FDA. Rare Diseases at FDA. 2024.https://www.fda.gov/patients/rare-diseases-fda [accessed: 11/16/2025].

4	Miller KL, Lanthier M. Investigating the landscape of US orphan product approvals. *Orphanet J Rare Dis* 2018;**13**:1–8.

5	Braun MM, Farag-El-Massah S, Xu K, Coté TR. Emergence of orphan drugs in the United States: a quantitative assessment of the first 25 years. Nat *Rev Drug Discov* 2010;**9**:519–22.

6	Giannuzzi V, Conte R, Landi A,  Coté TR. Orphan medicinal products in Europe and United States to cover needs of patients with rare diseases: an increased common effort is to be foreseen. *Orphanet J Rare Dis* 2017;**12**:1–11.

7	Murakami M, Narukawa M. Matched analysis on orphan drug designations and approvals: cross regional analysis in the United States, the European Union, and Japan. *Drug Discov Today* 2016;**21**:544–9.

8	Vokinger KN, Kesselheim AS. Application of orphan drug designation to cancer treatments (2008–2017): a comprehensive and comparative analysis of the USA and EU. *BMJ Open* 2019;**9**:e028634.

9	Iglesias-Lopez C, Obach M, Vallano A, Agustí A*.* Comparison of regulatory pathways for the approval of advanced therapies in the European Union and the United States. *Cytotherapy* 2021;**23**:261–74.

10	O’Connor DJ. Orphan drug designation – Europe, the USA and Japan. *Expert Opin Orphan Drugs* 2013;**1**:255–9.

11	Shields GE, Neves ACA, Bajpai SK. Orphan drug designation: a comparison of Policies, processes and results from the US and the EU. *Value Heal* 2015;**18**:A305.

12	Hall AK, Carlson MR. The current status of orphan drug development in Europe and the US. *Intractable rare Dis Res* 2014;**3**:1–7. doi:10.5582/irdr.3.1

13	Salcher-Konrad M, Naci H, Davis C. Approval of Cancer Drugs With Uncertain Therapeutic Value: A Comparison of Regulatory Decisions in Europe and the United States. *Milbank Q* 2020;**98**:1219–56.

14	Pammolli F, Righetto L, Abrignani S, Pani L, Pelicci PG, Rabosio E*.* The endless frontier? The recent increase of R&D productivity in pharmaceuticals. *J Transl Med* 2020;**18**:1–14.

15	DiMasi JA. Pharmaceutical R&D performance by firm size: Approval success rates and economic returns. *Am J Ther* 2014;**21**:26–34.

16	Hoekman J, Boon W. Changing standards for drug approval: A longitudinal analysis of conditional marketing authorisation in the European Union. *Soc Sci Med* 2019;**222**:76–83.

17	Lau F, Seifert R. Comparison of drug approvals of the FDA and EMA between 2013 and 2023. *Naunyn Schmiedebergs Arch Pharmacol* 2025;:1–21.

18	EMA. The European regulatory system for medicine: Bringing new safe and effective medicines to patients across the European Union. 2023. https://www.ema.europa.eu/en/documents/leaflet/european-regulatory-system-medicines_ en.pdf [accessed: 11/16/2025].

19	Hwang TJ, Ross JS, Vokinger KN, Kesselheim AS.. Association between FDA and EMA expedited approval programs and therapeutic value of new medicines: retrospective cohort study. *BMJ* 2020;**371**.

20	Informa Pharma Intellenge. Pharmaprojects. 2025.https://citeline.informa.com/ [accessed: 11/16/2025].

21	EMA. PRIME: 5 years experience. 2021.https://www.ema.europa.eu/en/documents/report/ prime-analysis-first-5-years-experience_en.pdf [accessed: 11/16/2025].

22	European Commission. Commission notice on the application of Articles 3, 5 and 7 of Regulation (EC) No 141/2000 on orphan medicinal products (2016/C 424/03). 2016. https://eur-lex.europa.eu/legal-content/EN/TXT/PDF/?uri=OJ:JOC_2016_424_R_0003&from= EN [accessed: 11/16/2025].

23	United States Congress. 21st Century Cures Act. Published Online First: 2016.https://www.congress.gov/114/plaws/publ255/PLAW-114publ255.pdf [accessed: 11/16/2025].

24	FDA. Orphan Drug Modernization Plan. 2017.https://www.fda.gov/industry/designating-orphan-product-drugs-and-biological-products/orphan-drug-modernization-plan [accessed: 11/16/2025].

25	Subbiah V. Tissue-agnostic cancer therapies: promise, reality, and the path forward. *Nat Commun* 2025;**16**:4972.

26	AlDoughaim M, AlSuhebany N, AlZahrani M, AlQahtani T, AlGhamdi S, Badreldin H, Al Alshaykh H*.* Cancer biomarkers and precision oncology: A review of recent trends and innovations. *Clin Med Insights Oncol* 2024;**18**:11795549241298540.

27	Suehnholz SP, Nissan MH, Zhang H, Kundra R, Nandakumar S, Lu C, *et al.* Quantifying the expanding landscape of clinical actionability for patients with cancer. *Cancer Discov* 2024;**14**:49–65.

28	Hunter NL, Rao GR, Sherman RE. Flexibility in the FDA approach to orphan drug development. *Nat Rev Drug Discov* 2017;**16**:737–8.

29	O’Connor DJ, Sheean ME, Hofer MP, Tsigkos S, Mariz S, Fregonese L, *et al.* Defining orphan conditions in the context of the European orphan regulation: challenges and evolution. *Nat Rev Drug Discov* 2019;**18**:479–80.

30	Lawler M, Keeling P, Kholmanskikh O, Minnaard W, Moehlig-Zuttermeister H, Normanno N, *et al*. Empowering effective biomarker-driven precision oncology: A call to action. *Eur J Cancer* 2024;**209**:114225.

31	Kesselheim AS, Treasure CL, Joffe S. Biomarker-defined subsets of common diseases: policy and economic implications of Orphan Drug Act coverage. *PLoS Med* 2017;**14**:e1002190.

32	FDA. Frequently Asked Questions (FAQ) About Designating an Orphan Product. 2023. https://www.fda.gov/industry/designating-orphan-product-drugs-and-biological-products/frequently-asked-questions-faq-about-designating-orphan-product [accessed: 1/28/2026].

33	EMA. Orphan designation: Overview. https://www.ema.europa.eu/en/human-regulatory-overview/orphan-designation-overview [accessed: 1/28/2026].

34	Tsigkos S, Llinares J, Mariz S, Aarum S, Fregonese L, Dembowska-Baginska B, *et al.* Use of biomarkers in the context of orphan medicines designation in the European Union. *Orphanet J Rare Dis* 2014;**9**:13. doi:10.1186/1750-1172-9-13

35	Covington. European Parliament adopts its Position on EU Pharma Law Review: 8 Key Takeaways for Industry. 2024. https://www.cov.com/en/news-and-insights/insights/2024/04/european-parliament-adopts-its-position-on-eu-pharma-law-review-8-key-takeaways-for-industry#layout=card [accessed: 11/16/2025].

36	Caetano R, Cordeiro M, Villardi P, Almeida Rodrigues PH, Garcia SOC. Dynamics of patents, orphan drug designation, licensing, and revenues from drugs for rare diseases: The market expansion of eculizumab. PloS one. 2021;**16**(3):e0247853.

37	Ku MS. Recent trends in specialty pharma business model. J Food Drug Anal 2015;23(4):595-608.

38	Schuhmacher A, Gassmann O, Hinder M. Changing R&D models in research-based pharmaceutical companies. *J Transl Med* 2016;**14**(1):105.

39	Brinkhuis F, Goettsch WG, Mantel-Teeuwisse AK, Bloem LT. Added benefit and revenues of oncology drugs approved by the European Medicines Agency between 1995 and 2020: retrospective cohort study. *BMJ* 2024;384.

40	Martinalbo J, Bowen D, Camarero J, Chapelin M, Démolis P, Foggi P, *et al*. Early market access of cancer drugs in the EU. *Ann Oncol* 2016;**27**(1):96-105.

41	Amaouche N, Salomé HC, Collignon O, Santos MR, Ziogas C. Marketing authorisation applications submitted to the European Medicines Agency by small and medium-sized enterprises: an analysis of major objections and their impact on outcomes. *Drug Discov Today* 2018;**23**:1801–5.

42	Mitchell P. Price controls seen as key to Europe’s drug innovation lag. *Nat Rev Drug Discov* 2007;**6**:257–9.

43	Danzon PM, Epstein AJ. Effects of regulation on drug launch and pricing in interdependent markets. In: *The Economics of Medical Technology*. Emerald Group Publishing Limited 2012. 35–71.

44	Lau F, Seifert R. Comparison of drug approvals of the FDA and EMA between 2013 and 2023. *Naunyn Schmiedebergs Arch Pharmacol* 2025;**3**:1-21.

45	European Commission. Reform of the EU pharmaceutical legislation. 2025. https://health.ec.europa.eu/medicinal-products/legal-framework-governing-medicinal-products-human-use-eu/reform-eu-pharmaceutical-legislation_en [accessed: 1/26/2026].

## S1 Table. Large and Medium Companies by Pharma Sales in 2023.

| **Company** | **HQ Location** | **Pharma Sales Rank** | | **Pharma Sales Value^1^ ($M)** | **Number of FDA Orphan Approvals** |
| --- | --- | --- | --- | --- | --- |
| **Tier 1** |  | |  |  |  |
| Pfizer | United States | | 1 | 58,496 | 38 |
| AbbVie | United States | | 2 | 53,842 | 18 |
| Merck&Co | United States | | 3 | 53,583 | 20 |
| Johnson & Johnson | United States | | 4 | 53,267 | 18 |
| Roche | Switzerland | | 5 | 49,688 | 49 |
| Novartis | Switzerland | | 6 | 45,440 | 52 |
| Bristol Myers Squibb | United States | | 7 | 44,398 | 62 |
| AstraZeneca | United Kingdom | | 8 | 43,789 | 38 |
| Sanofi | France | | 9 | 40,985 | 12 |
| GSK | United Kingdom | | 10 | 37,724 | 10 |
| Eli Lilly | United States | | 11 | 34,124 | 11 |
| Novo Nordisk | Denmark | | 12 | 33,722 | 5 |
| Takeda | Japan | | 13 | 29,482 | 26 |
| Amgen | United States | | 14 | 28,190 | 32 |
| Gilead Sciences | United States | | 15 | 27,116 | 23 |
| Boehringer Ingelheim | Germany | | 16 | 22,480 | 7 |
| Teva | Israel | | 17 | 15,844 | 3 |
| Viatris | United States | | 18 | 15,252 | 2 |
| Bayer | Germany | | 19 | 14,994 | 8 |
| CSL | Australia | | 20 | 14,100 | 9 |
| Astellas | Japan | | 21 | 11,108 | 12 |
| Daiichi Sankyo | Japan | | 22 | 10,027 | 3 |
| **Tier 2** |  | |  |  |  |
| Vertex | United States | | 23 | 9,869 | 23 |
| Otsuka | Japan | | 24 | 9,707 | 2 |
| Merck KGaA | Germany | | 26 | 8,711 | 2 |
| Biogen | United States | | 28 | 7,247 | 3 |
| Regeneron | United States | | 29 | 7,078 | 7 |
| Sun Pharmaceutical | India | | 33 | 5,749 | 1 |
| UCB | Belgium | | 34 | 5,681 | 4 |
| Abbott Laboratories | United States | | 35 | 5,039 | 1 |
| Eisai | Japan | | 36 | 4,590 | 3 |
| Fresenius | Germany | | 37 | 4,538 | 1 |
| Jazz | Ireland | | 39 | 3,737 | 10 |
| Ipsen | France | | 44 | 3,413 | 7 |
| Chiesi | Italy | | 46 | 3,273 | 8 |
| Incyte | United States | | 48 | 3,165 | 7 |
| Kyowa Kirin | Japan | | 49 | 3,154 | 4 |
| Mitsubishi Chemical | Japan | | 50 | 3,118 | 2 |
| Lundbeck | Denmark | | 55 | 2,824 | 3 |
| Ferring Pharmaceuticals | Switzerland | | 61 | 2,336 | 1 |
| United Therapeutics | United States | | 62 | 2,328 | 4 |
| BioMarin Pharmaceutical | United States | | 65 | 2,241 | 6 |
| BeiGene | China | | 66 | 2,190 | 1 |
| Recordati | Italy | | 69 | 2,081 | 4 |
| Mallinckrodt | Ireland | | 74 | 1,866 | 2 |
| Neurocrine Biosciences | United States | | 75 | 1,861 | 1 |
| Swedish Orphan Biovitrum | Sweden | | 76 | 1,783 | 5 |
| Exelixis | United States | | 78 | 1,629 | 3 |
| Pierre Fabre | France | | 89 | 1,303 | 1 |

**S2 Appendix. Strengthening the Reporting of Observational Studies in Epidemiology (STROBE) Checklist.**

|  | Item No | Recommendation | Section No |  |  |
| --- | --- | --- | --- | --- | --- |
| **Title and abstract** | 1 | (*a*) Indicate the study’s design with a commonly used term in the title or the abstract | Title & Abstract |  |  |
|  |  | (*b*) Provide in the abstract an informative and balanced summary of what was done and what was found | Abstract |  |  |
| Introduction | | | |  |  |
| Background/rationale | 2 | Explain the scientific background and rationale for the investigation being reported | 1 |  |  |
| Objectives | 3 | State specific objectives, including any prespecified hypotheses | 1 |  |  |
| Methods | | | |  |  |
| Study design | 4 | Present key elements of study design early in the paper | 2 |  |  |
| Setting | 5 | Describe the setting, locations, and relevant dates, including periods of recruitment, exposure, follow-up, and data collection | 2 |  |  |
| Participants | 6 | (*a*) Give the eligibility criteria, and the sources and methods of selection of participants. Describe methods of follow-up | 2 |  |  |
|  |  | (*b*) For matched studies, give matching criteria and number of exposed and unexposed | 2 |  |  |
| Variables | 7 | Clearly define all outcomes, exposures, predictors, potential confounders, and effect modifiers. Give diagnostic criteria, if applicable | 2 |  |  |
| Data sources/ measurement | 8* | For each variable of interest, give sources of data and details of methods of assessment (measurement). Describe comparability of assessment methods if there is more than one group | 2 |  |  |
| Bias | 9 | Describe any efforts to address potential sources of bias | 2 |  |  |
| Study size | 10 | Explain how the study size was arrived at | 2 |  |  |
| Quantitative variables | 11 | Explain how quantitative variables were handled in the analyses. If applicable, describe which groupings were chosen and why | 2 |  |  |
| Statistical methods | 12 | (*a*) Describe all statistical methods, including those used to control for confounding | 2 |  |  |
|  |  | (*b*) Describe any methods used to examine subgroups and interactions | 2 |  |  |
|  |  | (*c*) Explain how missing data were addressed | n/a |  |  |
|  |  | (*d*) If applicable, explain how loss to follow-up was addressed | n/a |  |  |
|  |  | (*e*) Describe any sensitivity analyses | n/a |  |  |
| Results | | |  |  |  |
| Participants | 13* | (a) Report numbers of individuals at each stage of study—eg numbers potentially eligible, examined for eligibility, confirmed eligible, included in the study, completing follow-up, and analysed | 3 |  |  |
|  |  | (b) Give reasons for non-participation at each stage | n/a |  |  |
|  |  | (c) Consider use of a flow diagram | n/a |  |  |
| Descriptive data | 14* | (a) Give characteristics of study participants (eg demographic, clinical, social) and information on exposures and potential confounders | 3 |  |  |
|  |  | (b) Indicate number of participants with missing data for each variable of interest | n/a |  |  |
|  |  | (c) Summarise follow-up time (eg, average and total amount) | 3 |  |  |
| Outcome data | 15* | Report numbers of outcome events or summary measures over time | 3 |  |  |
| Main results | 16 | (*a*) Give unadjusted estimates and, if applicable, confounder-adjusted estimates and their precision (eg, 95% confidence interval). Make clear which confounders were adjusted for and why they were included | 3 | |  |
|  |  | (*b*) Report category boundaries when continuous variables were categorized | n/a | |  |
|  |  | (*c*) If relevant, consider translating estimates of relative risk into absolute risk for a meaningful time period | n/a | |  |
| Other analyses | 17 | Report other analyses done—eg analyses of subgroups and interactions, and sensitivity analyses | 3 | |  |
| Discussion | | | | | |
| Key results | 18 | Summarise key results with reference to study objectives | 4 | |  |
| Limitations | 19 | Discuss limitations of the study, taking into account sources of potential bias or imprecision. Discuss both direction and magnitude of any potential bias | 4 | |  |
| Interpretation | 20 | Give a cautious overall interpretation of results considering objectives, limitations, multiplicity of analyses, results from similar studies, and other relevant evidence | 4 | |  |
| Generalisability | 21 | Discuss the generalisability (external validity) of the study results | 4 | |  |
| Other information | | | | | |
| Funding | 22 | Give the source of funding and the role of the funders for the present study and, if applicable, for the original study on which the present article is based | Financial Disclosure | |  |

*Give information separately for exposed and unexposed groups.

**Note:** An Explanation and Elaboration article discusses each checklist item and gives methodological background and published examples of transparent reporting. The STROBE checklist is best used in conjunction with this article (freely available on the Web sites of PLoS Medicine at http://www.plosmedicine.org/, Annals of Internal Medicine at http://www.annals.org/, and Epidemiology at http://www.epidem.com/). Information on the STROBE Initiative is available at http://www.strobe-statement.org.

**S3 Table. Characteristics of United States Food and Drug Administration (FDA) orphan approvals, 2011–2023. Values are numbers (percentages).**

| **Characteristics** | **EMA Orphan Marketing Authorisation** | **EMA Non-orphan Marketing Authorisation** | **Not Authorised by the EMA** |
| --- | --- | --- | --- |
| **Period** |  |  |  |
| 2011-2016 | 66(28) | 96(31) | 66(24) |
| 2017-2023 | 169(72) | 212(69) | 205(76) |
| **Therapeutic area** |  |  |  |
| Cancer | 89(38) | 178(58) | 86(32) |
| Non-cancer | 146(62) | 130(42) | 185(68) |
| **Company size** |  |  |  |
| Tier-1 | 123(52) | 233(76) | 99(37) |
| Tier-2 | 41(17) | 31(10) | 44(16) |
| Tier-3 | 71(30) | 44(14) | 128(47) |
| **Company HQ Location** |  |  |  |
| US | 124(53) | 165(54) | 172(64) |
| Europe | 89(38) | 122(40) | 74(27) |
| Other | 22(9) | 21(6) | 25(9) |
|  |  |  |  |

## S4 Table. Univariable GEE logistic regression analysis of the European Medicines Agency (EMA) regulatory status of United States (US) orphan approvals, 2011–2023. Values are odds ratios (95% confidence intervals).

|  | **Odds of FDA orphan approvals authorised**  **by the EMA** | **P value** | **Odds of FDA orphan approvals authorised with**  **orphan designations**  **by the EMA** | **P value** |
| --- | --- | --- | --- | --- |
| **Period** |  |  |  |  |
| 2011-2016 | 1(reference) |  | 1 (reference) |  |
| 2017-2023 | 0.66 (0.48–0.92) | 0.013 | 1.17 (0.86, 1.60) | 0.314 |
| **Therapeutic area** |  |  |  |  |
| Cancer | 1(reference) |  | 1(reference) |  |
| Non-cancer | 0.53 (0.38, 0.75) | <0.001 | 2.36 (1.50, 3.70) | <0.001 |
| **Company size** |  |  |  |  |
| Tier-1 | 1(reference) |  | 1(reference) |  |
| Tier-2 | 0.45 (0.28–0.74) | 0.001 | 2.14 (1.17, 3.90) | 0.014 |
| Tier-3 | 0.29 (0.20–0.43) | <0.001 | 2.95 (1.75, 4.99) | <0.001 |
| **Company HQ location** |  |  |  |  |
| US | 1(reference) |  | 1(reference) |  |
| Europe | 1.69 (1.17–2.43) | 0.005 | 1.31 (0.85, 2.02) | 0.223 |
| Other | 1.22 (0.68–2.19) | 0.507 | 1.37 (0.73, 2.56) | 0.332 |

## S5 Figure. Orphan drug approvals by the United States Food and Drug Administration (FDA) and Corresponding European Medicines Agency (EMA) regulatory outcomes, 2011–2016 and 2017–2023.

|  | Not authorised by the EMA | Non-orphan marketing authorisation | Orphan marketing authorisation |
| --- | --- | --- | --- |
| 2017-2023 | 205 | 212 | 169 |
| 2011-2016 | 66 | 96 | 66 |

## S6 Appendix. Therapeutic areas of United States Food and Drug Administration (FDA) orphan drug approvals and related European Medicines Agency (EMA) regulatory outcomes, 2011-2023.

## S6A Figure. Distribution of United States Food and Drug Administration (FDA) orphan approvals across therapeutic areas, 2011–2023.

|  | 2011 | 2012 | 2013 | 2014 | 2015 | 2016 | 2017 | 2018 | 2019 | 2020 | 2021 | 2022 | 2023 |
| --- | --- | --- | --- | --- | --- | --- | --- | --- | --- | --- | --- | --- | --- |
| Cancer | 11 | 10 | 10 | 24 | 21 | 20 | 45 | 43 | 25 | 41 | 37 | 34 | 32 |
| Neurologic and autoimmune | 6 | 2 | 4 | 6 | 8 | 5 | 9 | 21 | 13 | 17 | 15 | 14 | 14 |
| Sensory and others | 6 | 6 | 3 | 7 | 3 | 9 | 8 | 9 | 14 | 9 | 10 | 6 | 14 |
| Blood and cardiovascular | 2 | 1 | 12 | 10 | 5 | 3 | 4 | 11 | 8 | 4 | 8 | 6 | 13 |
| Alimentary and metabolism |  | 2 | 2 | 3 | 6 | 2 | 5 | 2 | 2 | 9 | 7 | 6 | 8 |
| Anti-infective | 1 | 4 | 3 |  | 4 |  | 5 | 4 | 8 | 4 | 7 | 3 | 8 |
| Respiratory |  | 1 |  | 2 | 2 | 2 | 5 | 3 | 5 | 6 | 1 | 2 | 2 |

**S6B Table. Univariable GEE logistic regression analysis among therapeutic areas, 2011–2023. Values are odds ratios (95% confidence intervals).**

|  | **Odds of FDA orphan approvals authorised**  **by the EMA** | **P value** | **Odds of FDA orphan approvals authorised with**  **orphan designations**  **by the EMA** | **P value** |
| --- | --- | --- | --- | --- |
| **Therapeutic area** |  |  |  |  |
| Cancer | 1(reference) |  | 1(reference) |  |
| Neurologic and autoimmune | 0.38 (0.24, 0.61) | <0.001 | 1.30 (0.70, 2.40) | 0.401 |
| Sensory and others | 0.34 (0.21, 0.57) | <0.001 | 1.68 (0.88, 3.21) | 0.115 |
| Blood and cardiovascular | 0.74 (0.43, 1.27) | 0.274 | 0.75 (0.40, 1.40) | 0.363 |
| Alimentary and metabolism | 1.46 (0.68, 3.12) | 0.331 | 3.25 (1.53, 6.90) | 0.002 |
| Anti-infective | 0.64 (0.32, 1.25) | 0.190 | 1.36 (0.62, 2.96) | 0.444 |
| Respiratory | 0.89 (0.32, 2.52) | 0.831 | 1.54 (0.43, 5.51) | 0.503 |

**S7 Text. The study on the Food and Drug Administration (FDA) regulatory status of European Medicines Agency (EMA) orphan marketing authorisations from 2011-2023**

Of the EMA marketing authorisations with orphan designation from 2011-2023, all had orphan designation from the FDA. Only 12% did not yet have FDA approval at the time of EMA authorisation. Some of the EMA orphan marketing authorisations not FDA approved were subsequently approved by the FDA in 2024–Sep 2025, reducing the contemporaneous gap to 10% (17). By contrast, the share of FDA orphan approvals not authorised by the EMA over the same period is substantially higher 71% (579), and 70% (549) when taking the EMA approval lag into account.
