# Supplementary material for: Discordance in orphan drug approvals between the U.S. Food and Drug Administration and the European Medicines Agency: A retrospective observational analysis
Source: PLoS Med. 2026 Jul 6;23(7):e1004861. doi: 10.1371/journal.pmed.1004861 (PMC13375132; doi:10.1371/journal.pmed.1004861)
Supplement: Attachment — Submitted filename: Responses to editors_6.2.2026.docx [file pmed.1004861.s013.docx]

**Responses to editors and reviewers**

We thank the editor and reviewers for their constructive comments and valuable feedback, which have helped improve the manuscript. The reviewers did not request any further changes in this round of review. We have addressed all comments from the editors and responded to each point individually.

------------------------------------------------------------

**Requests from Editors:**
*In the final bullet point of  the Author Summary, in ‘What Do These Findings Mean?’ Please include the main limitations of the study in non-technical language.
Please see our author guidelines for more information: <https://journals.plos.org/plosmedicine/s/revising-your-manuscript#loc-author-summary>.

Thanks, the limitation has been added.

* Please confirm that your title complies with PLOS Medicine's style. Your title must be nondeclarative and not a question. It should begin with main concept if possible. "Effect of" should be used only if causality can be inferred, i.e., for an RCT. Please place the study design ("A randomized controlled trial," "A retrospective study," "A modelling study," etc.) in the subtitle (ie, after a colon). Please see the suggested revised title.

Thanks, the title has been changed based on the editor's suggestions.

* Please confirm that your abstract complies with our requirements, including format (three sections: Background, Methods and Findings, and Conclusions) and providing all the information relevant to this study type <https://journals.plos.org/plosmedicine/s/submission-guidelines#loc-abstract>

Thanks, confirmed.

* Please ensure that the Introduction ends with a clear description of the study question or hypothesis.

Thanks, all checked.

* Please ensure that all abbreviations are defined at first use throughout the text (e.g. HTA).

Thanks, all checked.

* Please confirm that all numbers presented in the abstract are present and identical to numbers presented in the main manuscript text.
Thanks, all checked.

GENERAL

* Please disclose any use of AI tools and technologies to the study or article content in the Methods, including the name(s) of any tool(s) used and a description of their use. Please see our guidelines for more information: <https://journals.plos.org/plosmedicine/s/ethical-publishing-practice#loc-artificial-intelligence-tools-and-technologies>.

No AI tools has been used for this research.

* Please review your text for claims of novelty or primacy (e.g. 'for the first time') and remove this language. In addition, please check that any use of statistical terms (such as trend or significant) are supported by the data, and if not please remove them.

Thanks, the novelty and primary language has been removed.

* Statistical reporting: Please revise throughout the manuscript, including tables and figures.
- Please report statistical information as follows to improve clarity for the reader "22% (95% CI [13,28]; p</=)".
- Please separate upper and lower bounds with commas instead of hyphens as the latter can be confused with reporting of negative values.
- Please repeat statistical definitions (HR, CI etc.) for each set of parentheses.

All checked and corrected.

* In the abstract, please include the important dependent variables that are adjusted for in the analyses.

We have revised the abstract to specify the variables examined in univariable analysis. Separate univariable logistic regression models were conducted for each dependent variable; therefore, no covariates were adjusted for.

* In the author summary, in the final bullet point of 'What Do These Findings Mean?', please include the main limitations of the study in non-technical language.
* Please remove all causal language from the study and use associative language.

The main limitation has been added and causal language has been removed.

FUNDING STATEMENT
* The funding statement should include: specific grant numbers, initials of authors who received each award, URLs to sponsors’ websites. Also, please state whether any sponsors or funders (other than the named authors) played any role in study design, data collection and analysis, the decision to publish, or preparation of the manuscript. If they had no role in the research, include this sentence: “The funders had no role in study design, data collection and analysis, decision to publish, or preparation of the manuscript.” A URL for NovoNordisk is missing.

The statement has been changed following the suggestions.

COMPETING INTERESTS STATEMENT
* All authors must declare their relevant competing interests per the PLOS policy, which can be seen here: <https://journals.plos.org/plosmedicine/s/competing-interests> For authors with ties to industry, please indicate whether any of the interests has a financial stake in the results of the current study.
*Please amend your competing interests statement. Funding information should go to the relevant section and include URLs.

The competing interest statement has been added.

DATA AVAILABILITY
* PLOS defines the “minimal data set” to consist of the data set used to reach the conclusions drawn in the manuscript with related metadata and methods, and any additional data required to replicate the reported study findings in their entirety. Authors do not need to submit their entire data set, or the raw data collected during an investigation. Please submit the following data:
The values behind the means, standard deviations and other measures reported;
The values used to build graphs;
The points extracted from images for analysis.

The data availability statement has been added.

* Thank you for agreeing to make your data available. At this time, please provide the link to the data repository and accession numbers required for access.
* If new code was used to generate results, please deposit the code in a public repository and include the statement on code availability in the data availability statement. Please see guidance here:<https://journals.plos.org/plosmedicine/s/code-sharing-guidance#loc-data-availability-statement-examples>
Data availability statement has been added.

Tables and Figures
* When a p value is given, please specify the statistical test used to determine it in the legend.
* Please specify the variables controlled for in all relevant Tables
OBSERVATIONAL, COHORT, CROSS-SECTIONAL, AND CASE CONTROL STUDIES
* Please ensure that the study is reported according to the STROBE guideline, and include the completed STROBE checklist as Supporting Information. Please add the following statement, or similar, to the Methods: "This study is reported as per the Strengthening the Reporting of Observational Studies in Epidemiology (STROBE) guideline (S1 Checklist)."
The STROBE guideline can be found here: <http://www.equator-network.org/reporting-guidelines/strobe/>
When completing the checklist, please use section and paragraph numbers, rather than page numbers.
All checked.


**Comments from Reviewers:**

Reviewer #2: The authors have already addressed my comments.

Reviewer #3: Thanks for the revised manuscript and responses to my original review.
The revised manuscript and responses to my questions have resolved my original questions.

We appreciate that Reviewers 2 and 3 were positive about the revised manuscript.

**Technical requests:**

1) Please amend your detailed Financial Disclosure statement. This is published with the article, therefore should be completed in full sentences and contain the exact wording you wish to be published.
i). State what role the funders took in the study. If the funders had no role in your study, please state: “The funders had no role in study design, data collection and analysis, decision to publish, or preparation of the manuscript.”
ii). If any authors received a salary from any of your funders, please state which authors and which funders.
The statement has been changed following the suggestions.

2) We notice that your [supplementary materials] are included in the manuscript file. Please remove them and upload them with the file type 'Supporting Information'. Please ensure that each Supporting Information file has a legend listed in the manuscript after the references list.
The supplementary materials have been removed from the manuscript.

3) We note that your Supplementary materials are duplicated on your submission as they included in the manuscript file and are also uploaded separately as Supporting Information' file. Please remove them and upload them with the file type 'Supporting Information' and ensure that each Supporting Information file are included only Once
The supplementary materials have been removed from the manuscript.

4) In your Data Availability Statement, please ensure you have provided all necessary access information in complete sentences. Access information should include the name of any data repositories used as well as digital object identifiers (DOIs), accession numbers or codes, or other persistent identifiers for all relevant data. If all data has been provided within your submission files, please use the statement "All relevant data is available within the manuscript and Supporting Information files." The Data Availability Statement will appear on the published version of your manuscript.
The data availability statement has been added with DOIs.
