# Supplementary material for: Discordance in orphan drug approvals between the U.S. Food and Drug Administration and the European Medicines Agency: A retrospective observational analysis
Source: PLoS Med. 2026 Jul 6;23(7):e1004861. doi: 10.1371/journal.pmed.1004861 (PMC13375132; doi:10.1371/journal.pmed.1004861)
Supplement: Peer review history

# Editor report: Decision Letter 0

**DOI:** 10.1371/journal.pmed.1004861.r001

**Authors:** Alison Farrell

16 Dec 2024

Dear Dr Martin,

Thank you for submitting your manuscript entitled "Factors influencing the growing discordance in orphan approvals between the US Food and Drug Administration and European Medicines Agency: retrospective cohort study 2011-2020" for consideration by PLOS Medicine.

Your manuscript has now been evaluated by the PLOS Medicine editorial staff as well as by an academic editor with relevant expertise and I am writing to let you know that we would like to send your submission out for external peer review.

However, before we can send your manuscript to reviewers, we need you to complete your submission by providing the metadata that is required for full assessment. To this end, please login to Editorial Manager where you will find the paper in the 'Submissions Needing Revisions' folder on your homepage. Please click 'Revise Submission' from the Action Links and complete all additional questions in the submission questionnaire.

Please re-submit your manuscript within two working days, i.e. by Dec 18 2024 11:59PM.

Login to Editorial Manager here: https://www.editorialmanager.com/pmedicine

Once your full submission is complete, your paper will undergo a series of checks in preparation for peer review. Once your manuscript has passed all checks it will be sent out for review.

Feel free to email us at plosmedicine@plos.org if you have any queries relating to your submission.

Kind regards,

Alison Farrell, Ph.D.

Senior Editor

PLOS Medicine

# Peer review: Decision Letter 1

**DOI:** 10.1371/journal.pmed.1004861.r002

**Authors:** Alison Farrell, Alison Farrell

1 Aug 2025

Dear Dr Martin,

Many thanks for submitting your manuscript "Factors influencing the growing discordance in orphan approvals between the US Food and Drug Administration and European Medicines Agency: retrospective cohort study 2011-2020" (PMEDICINE-D-24-04261R1) to PLOS Medicine. I apologize for the extended delay in conveying to you our decision, as we hoped to receive additional expert input that I regret has not been forthcoming. The paper has been reviewed by subject experts; their comments are included below and can also be accessed here:

As you will see, the reviewers the reviewers find the topic important but recommend substantive revisions to strengthen the work. In particular the reviewers request that the analysis is updated to 2025, that you consider a comparison to non-orphan drugs in order to ascertain whether or not the underlying factors are specific to orphan drugs, provide additional discussion of the differences in the European and US regulatory criteria and paths, and address issues of potential bias and confounding. After discussing the paper with the editorial team, I would like to invite you to revise the paper in response to the reviewers' comments. We plan to send the revised paper to some or all of the original reviewers, and we cannot provide any guarantees at this stage regarding publication. Please note that we ask you to strengthen the reporting of the methodology and statistics, and we will seek the input of a statistical reviewer of a revised manuscript.

In addition to these revisions, you may need to complete some formatting changes, which you will receive in a follow up email. A member of our team will be in touch with a set of requests shortly. If you do not receive a separate email within a few days, please assume that checks have been completed, and no additional changes are required.

When you upload your revision, please include a point-by-point response that addresses all of the reviewer and editorial points, indicating the changes made in the manuscript and either an excerpt of the revised text or the location (eg: page and line number) where each change can be found. Please also be sure to check the general editorial comments at the end of this letter and include these in your point-by-point response. When you resubmit your paper, please include a clean version of the paper as the main article file and a version with changes tracked as a marked-up manuscript. It may also be helpful to check the guidelines for revised papers at http://journals.plos.org/plosmedicine/s/revising-your-manuscript for any that apply to your paper.

We ask that you submit your revision by Aug 22 2025 11:59PM. However, if this deadline is not feasible, please contact me by email, and we can discuss a suitable alternative.

Don't hesitate to contact me directly with any questions (afarrell@plos.org).

Best regards,

Alison

Alison Farrell, Ph.D.

Senior Editor

PLOS Medicine

afarrell@plos.org

-----------------------------------------------------------

Comments from the reviewers:

Reviewer #1: Thank you for the opportunity to review this study which concerns a relevant research topic in the field.

This is a well written and easy to read paper that acknowledges the known growing discordance between the EU and USA in a number of areas in the pharmaceutical sector. However there are a number of substantial structural and methodological concerns that will need to be addressed before this paper can be considered for publication.

1.It is not clear why the authors selected the period of 2011-2020, please provide some context

2.A major issue with the paper is that based on the methodology, it is difficult to conclude on what is orphan specific and what is not related to orphans. Therefore comparison to non-orphan status in order to determine the true factors that are specific to orphans is needed. Please comment on the feasibility of conducting this analysis

3.The structure of the results and the discussion - conclusion should more easily and clearly pull out factors that are specific to orphans and factors that are applicable more generally to all or most pharmaceutical products e.g. size of company

4.A major gap for a paper with a focus on the FDA and EMA, is that the paper does not address sufficiently the differences in the regulatory orphan criteria and orphan process. As an example, significant benefit is not mentioned and this is considered to be a major hurdle for the EU designation versus the USA - please discuss all the specific factors for orphan designation in detail as per your statement in line 74.

5.Please consider the impact of the EU not allowing orphan and non-orphan status on the same marketing authorisation

6.The methodology would benefit from a much clearer narrative and description - e.g. line 151 states designated or authorised yet the results focus only on authorised? the section on statistical analysis is far too brief to understand what analysis was conducted and how

7.Lines 201-202 - the CHMP minutes and agendas provide substantial information. Please review this source of data regarding the statement that the EMA does not disclose data on the number of submissions it receives

8.The fact you reviewed only centrally authorised drugs is not a study limitation - as you indicate orphan designated products are mandated CP scope in the EU - this can be included in your methods if needs be.

9.It would be helpful to have a key (S3 and S4) and figure legend text for the S material

Reviewer #2: This study investigates the regulatory discrepancies between the FDA and EMA for orphan drug approvals. The analysis identifies factors contributing to the divergence and highlights implications for rare disease patient access in the US and EU. While the study presents valuable insights, several methodological and interpretative aspects warrant further discussion and clarification.

Major comments:

1. Methods, the authors included orphan approvals granted by the FDA between 1/1/2011 and 12/31/2020. Given that it is now 2025, it would be beneficial to include more recent data. Analyzing the period beyond 2020 could provide valuable insights into whether the divergence between the FDA and EMA has continued to grow or if their decisions have begun to align more closely. Since this data is publicly available, the authors should clarify why the most recent years were excluded and discuss how the inclusion of more recent data might affect the study's findings and conclusions.

2. Methods, the authors targeted a cohort of orphan approvals granted by the FDA to examine factors influencing the growing discordance in orphan approvals between the US FDA and EMA. However, this approach may introduce sample selection bias, as it excludes orphan approvals granted by the EMA but not by the FDA. These drugs also contribute to the observed discordance and could provide additional insights into regulatory differences and approval dynamics. Including or at least discussing the implications of this reverse scenario would strengthen the comprehensiveness of the analysis.

3. Methods, the study excludes FDA orphan approvals withdrawn or revoked before 2021. While this exclusion is reasonable, further discussion on potential biases this might introduce (e.g., exclusion of drugs with complex approval histories) is warranted.

4. Methods, the study included company size, location of headquarters, and therapeutic area as explanatory variables. However, it's unclear how these factors were chosen. Were these factors identified based on prior literature, data availability, or exploratory analysis? The selection process should be explicitly justified to ensure these variables are meaningful predictors. It is unclear whether these factors were chosen based on theoretical frameworks, prior research, or data-driven exploration. Additionally, the model does not address potential unmeasured confounders, such as pricing strategies, length of time for different approval types between FDA and EMA, or the clinical evidence supporting each drug, which could bias the results. While odds ratios provide insight into associations, the authors should consider reporting metrics like pseudo-R² or AIC/BIC to evaluate the model's fit and quantify how much variance the included factors explain. For example, if the model only explains a small proportion of the variance in EMA decisions, this suggests that many factors influencing approvals remain unmeasured. Regression models assume that all relevant variables are included or that unmeasured factors do not systematically bias results. If critical predictors like pricing strategies or clinical evidence for each drug are missing, the conclusions drawn from the regression may be incomplete or misleading. Therefore, acknowledging the potential role of omitted variables and exploring their likely impact on findings through sensitivity analyses would strengthen the validity of the conclusions drawn from the regression analysis. If the authors cannot justify why they used the logistic regression here, I would recommend just use descriptive analysis.

5. Implications, the authors argue that European patients may be missing out on treatments available in the US due to the divergence in regulatory outcomes. While the findings suggest limited access to certain drugs in Europe, the extent to which European patients are significantly disadvantaged is not empirically analyzed. For instance, the authors do not quantify the clinical impact or assess the real-world effectiveness of the non-submitted or non-authorized drugs in routine care. Additionally, there is no direct evaluation of how this divergence contributes to unmet patient needs. The authors could also discuss the possibility of over-approval by the US FDA, which may lead to discrepancies in access that are not solely due to limitations in the EU system.

Minor comments:

1. Abstract, the authors could improve clarity by separating the "Methods" and "Findings" into distinct sections within the abstract. The methods section should provide additional details, such as the specific databases used, the factors included in the regression model, and whether any subgroup or sensitivity analyses were conducted. Moreover, since logistic regression is mentioned in the methods, the main findings should report key regression results, including odds ratios, 95% confidence intervals, and p-values, to support the interpretation of the results.

2. Results, in S2 Appendix, can the authors explain the p-values for the rows corresponding to therapeutic area, company size, and company HQ location, for example Therapeutic area p=0.002 and p<0.001? Additionally, it would be helpful to include another table or add columns to the existing table to display the unadjusted percentages of FDA orphan approvals authorized by the EMA and FDA orphan approvals authorized with orphan designations by the EMA. This would allow readers to compare unadjusted and adjusted results more transparently.

Any attachments provided with reviews can be seen via the following link:

--------------------------------------------------------- ---

General editorial requests:

(Note: not all will apply to your paper, but please check each item carefully)

*We ask every co-author listed on the manuscript to fill in a contributing author statement, making sure to declare all competing interests. If any of the co-authors have not filled in the statement, we will remind them to do so when the paper is revised. If all statements are not completed in a timely fashion this could hold up the re-review process. If new competing interests are declared later in the revision process, this may also hold up the submission. Should there be a problem getting one of your co-authors to fill in a statement we will be in contact. Please do not add or remove authors without first discussing this with the handling editor. You can see our competing interests policy here: http://journals.plos.org/plosmedicine/s/competing-interests.

*Please upload any figures associated with your paper as individual TIF or EPS files with 300dpi resolution at resubmission; please read our figure guidelines for more information on our requirements: http://journals.plos.org/plosmedicine/s/figures. While revising your submission, please upload your figure files to the PACE digital diagnostic tool, https://pacev2.apexcovantage.com/. PACE helps ensure that figures meet PLOS requirements. To use PACE, you must first register as a user. Then, login and navigate to the UPLOAD tab, where you will find detailed instructions on how to use the tool. If you encounter any issues or have any questions when using PACE, please email us at PLOSMedicine@plos.org.

*Please ensure that the paper adheres to the PLOS Data Availability Policy (see http://journals.plos.org/plosmedicine/s/data-availability), which requires that all data underlying the study's findings be provided in a repository or as Supporting Information. For data residing with a third party, authors are required to provide instructions with contact information (web or email address) for obtaining the data. Please note that a study author cannot be the contact person for the data. PLOS journals do not allow statements supported by "data not shown" or "unpublished results." For such statements, authors must provide supporting data or cite public sources that include it.

*We expect all researchers with submissions to PLOS in which author-generated code underpins the findings in the manuscript to make all author-generated code available without restrictions upon publication of the work. In cases where code is central to the manuscript, we may require the code to be made available as a condition of publication. Authors are responsible for ensuring that the code is reusable and well documented. Please make any custom code available, either as part of your data deposition or as a supplementary file. Please add a sentence to your data availability statement regarding any code used in the study, e.g. "The code used in the analysis is available from Github [URL] and archived in Zenodo [DOI link]" Please review our guidelines at https://journals.plos.org/plosmedicine/s/materials-software-and-code-sharing and ensure that your code is shared in a way that follows best practice and facilitates reproducibility and reuse. Because Github depositions can be readily changed or deleted, we encourage you to make a permanent DOI'd copy (e.g. in Zenodo) and provide the URL.

*[EDITOR: CHECK FINANCIAL DISCLOSURES, COI, DAS, AND ETHICS STATEMENTS AND INCLUDE ANY NECESSARY REQUESTS]

*Please ensure that the study is reported according to the [XXXX] guideline and include the completed [XXXX] checklist as Supporting Information. When completing the checklist, please use section and paragraph numbers, rather than page numbers. Please add the following statement, or similar, to the Methods: "This study is reported as per [XXXX] guideline (S1 Checklist)."

FORMATTING - GENERAL

*Abstract: Please structure your abstract using the PLOS Medicine headings (Background, Methods and Findings, Conclusions). Please combine the Methods and Findings sections into one section.

*At this stage, we ask that you include a short, non-technical Author Summary of your research to make findings accessible to a wide audience that includes both scientists and non-scientists. The Author Summary should immediately follow the Abstract in your revised manuscript. This text is subject to editorial change and should be distinct from the scientific abstract. Ideally each sub-heading should contain 2-3 single sentence, concise bullet points containing the most salient points from your study. In the final bullet point of 'What Do These Findings Mean?', please include the main limitations of the study in non-technical language. Please see our author guidelines for more information: https://journals.plos.org/plosmedicine/s/revising-your-manuscript#loc-author-summary.

*Please express the main results with 95% CIs as well as p values. When reporting p values please report as p<0.001 and where higher as the exact p value p=0.002, for example. Throughout, suggest reporting statistical information as follows to improve clarity for the reader "22% (95% CI [13%,28%]; p</=)". Please be sure to define all numerical values at first use.

*Please include page numbers and line numbers in the manuscript file. Use continuous line numbers (do not restart the numbering on each page).

*Please cite the reference numbers in square brackets. Citations should precede punctuation.

FIGURES AND TABLES

*Please provide titles and legends for all figures and tables (including those in Supporting Information files).

*Please define all abbreviations used in each figure/table (including those in Supporting Information files).

*Please consider avoiding the use of red and green in order to make your figure more accessible to those with color blindness.

SUPPLEMENTARY MATERIAL

*Please note that supplementary material will be posted as supplied by the authors. Therefore, please amend it according to the relevant comments outlined here.

*Please cite your Supporting Information as outlined here: https://journals.plos.org/plosmedicine/s/supporting-information

REFERENCES

*PLOS uses the numbered citation (citation-sequence) method and first six authors, et al.

*Please ensure that journal name abbreviations match those found in the National Center for Biotechnology Information (NCBI) databases (http://www.ncbi.nlm.nih.gov/nlmcatalog/journals), and are appropriately formatted and capitalised.

*Where website addresses are cited, please include the complete URL and specify the date of access (e.g. [accessed: 12/06/2023]).

*Please also see https://journals.plos.org/plosmedicine/s/submission-guidelines#loc-references for further details on reference formatting.

[STUDY TYPE-SPECIFIC REQUESTS - DELETE SECTIONS AS NECESSARY]

RCTs [REFER TO RCT CHECKLIST AND MEETING NOTES FOR DETAILS TO ADD]

*PLOS Medicine requires that all trials be prospectively registered in one of registries recognized by WHO. Please ensure that study registration details are included in the Methods section.

*Please structure the Methods section using the following sub-headings: Study design and participants, Randomization and masking, Procedures, Outcomes, Statistical analysis.

*The following outcomes measures [ADD DETAILS AS NEEDED OR DELETE BULLET POINT] appear to differ between the submitted manuscript and the protocol [and/or trial registry]. Please clarify and explain all discrepancies between the paper and protocol. If the outcomes were not prespecified in the protocol, please define them in the Methods (Outcomes section) as post hoc and explain why they were added. Post-hoc comparisons should be presented as hypothesis generating rather than conclusive.

*Please ensure that all prespecified outcomes (primary, secondary, and exploratory) are listed in the Methods/Outcomes section and indicate whether there are outcomes that are not presented in the current report.

*Please specify the dates (Month Day, Year) during which study enrollment and follow up occurred.

*Please include absolute numbers wherever you report percentages; eg, n/N (%)

*Please present the safety data for the study including numbers of specific events and whether or not adverse events are thought to be related to treatment. AEs should be reported in the abstract, per CONSORT and CONSORT-Harms.

*Please complete the CONSORT checklist (https://www.equator-network.org/reporting-guidelines/consort/) and ensure that all components of CONSORT are present in the manuscript, including how randomization was performed, allocation concealment, blinding of intervention, definition of lost to follow-up, power statement. When completing the checklist, please use section and paragraph numbers, rather than page numbers.

*Please report your abstract according to CONSORT for abstracts, following the PLOS Medicine abstract structure (Background, Methods and Findings, Conclusions) https://www.equator-network.org/reporting-guidelines/consort-abstracts/

*If your trial had to undergo important modifications in response to extenuating circumstances, please complete the CONSERVE-CONSORT checklist and provide in your Supporting Information; (https://www.equator-network.org/reporting-guidelines/guidelines-for-reporting-trial-protocols-and-completed-trials-modified-due-to-the-covid-19-pandemic-and-other-extenuating-circumstances-the-conserve-2021-statement/). When completing the checklist, please use section and paragraph numbers, rather than page numbers.

*In keeping with our commitment to Open Science, please include the study protocol document and analysis plan (including any amendments) as Supporting Information to be published with the manuscript if accepted.

*Please note that PLOS Medicine requires prospective, public registration of a data sharing plan (as part of mandatory clinical trials registration) for all clinical trials that began enrollment on or after January 1, 2019, in accordance with ICMJE requirements.

OBSERVATIONAL STUDIES

*Abstract: Please include the study design, population and setting, number of participants, years during which the study took place (enrollment and follow up), length of follow up, and main outcome measures.

*Please ensure that the study is reported according to the STROBE (or appropriate STOBE extension) guideline (available from: https://www.equator-network.org/reporting-guidelines/strobe) and include the completed STROBE (or STROBE extension) checklist as Supporting Information. Please add the following statement, or similar, to the Methods: "This study is reported as per the Strengthening the Reporting of Observational Studies in Epidemiology (STROBE) guideline (S1 Checklist)." When completing the checklist, please use section and paragraph numbers, rather than page numbers.

*[FOR POPULATION HEALTH/REGISTRY STUDIES] Please ensure that the study is reported according to the RECORD guideline (available from https://www.record-statement.org) and include the completed checklist as Supporting Information. Please add the following statement, or similar, to the Methods: "This study is reported as per the Reporting of Studies Conducted using Observational Routinely-Collected Data (RECORD) guideline (S1 Checklist)." When completing the checklist, please use section and paragraph numbers, rather than page numbers.

*[FOR POPULATION HEALTH ESTIMATES] Please ensure that the study is reported according to the GATHER statement (available from https://www.equator-network.org/reporting-guidelines/gather-statement) and include the completed checklist as Supporting Information. Please add the following statement, or similar, to the Methods: "This study is reported as per the Guidelines for Accurate and Transparent Health Estimates Reporting (GATHER) statement (S1 Checklist)." When completing the checklist, please use section and paragraph numbers, rather than page numbers.

*[FOR MEDIATION ANALYSES] We recommend that the study is reported according to the AGReMA statement (https://agrema-statement.org/#:~:text=AGReMA%20is%20an%20evidence%2D%20and,randomised%20trials%20and%20observational%20studies) and include the completed checklist as Supporting Information. Please add the following statement, or similar, to the Methods: "This study is reported as per the Guideline for Reporting Mediation Analyses (AGReMA) statement (S1 Checklist)." When completing the checklist, please use section and paragraph numbers, rather than page numbers.

*For all observational studies, in the manuscript text, please indicate: (1) the specific hypotheses you intended to test, (2) the analytical methods by which you planned to test them, (3) the analyses you actually performed, and (4) when reported analyses differ from those that were planned, transparent explanations for differences that affect the reliability of the study's results. If a reported analysis was performed based on an interesting but unanticipated pattern in the data, please be clear that the analysis was data driven.

*Please state in the Methods section whether the study had a prospective protocol or analysis plan. If a prospective analysis plan (from your funding proposal, IRB or other ethics committee submission, study protocol, or other planning document written before analyzing the data) was used in designing the study, please include the relevant document(s) with your revised manuscript as a Supporting Information file to be published alongside your study and cite it in the Methods section. A legend for this file should be included at the end of your manuscript. If no such document exists, please make sure that the Methods section transparently describes when analyses were planned, and when/why any data-driven changes to analyses took place. Changes in the analysis, including those made in response to peer review comments, should be identified as such in the Methods section of the paper, with rationale.

MODELLING STUDIES

The following list is derived from Geoffrey P Garnett, Simon Cousens, Timothy B Hallett, Richard Steketee, Neff Walker. Mathematical models in the evaluation of health programmes. (2011) Lancet DOI:10.1016/S0140-6736(10)61505-X:

*If pertinent, please provide a diagram that shows the model structure, including how the natural history of the disease is represented, the process and determinants of disease acquisition, and how the putative intervention could affect the system.

*Please provide a complete list of model parameters, including clear and precise descriptions of the meaning of each parameter, together with the values or ranges for each, with justification or the primary source cited and important caveats about the use of these values noted.

*Please provide a clear statement about how the model was fitted to the data, including goodness-of-fit measure, the numerical algorithm used, which parameter varied, constraints imposed on parameter values, and starting conditions.

*For uncertainty analyses, please state the sources of uncertainties quantified and not quantified [can include parameter, data, and model structure].

*Please provide sensitivity analyses to identify which parameter values are most important in the model. Uncertainty estimates seek to derive a range of credible results on the basis of an exploration of the range of reasonable parameter values. The choice of method should be presented and justified.

*Please discuss the scientific rationale for the choice of model structure and identify points where this choice could influence conclusions drawn. Please also describe the strength of the scientific basis underlying the key model assumptions.

*For studies that develop a prediction model or evaluate its performance, please ensure that the study is reported according to the TRIPOD statement (https://www.equator-network.org/reporting-guidelines/tripod-statement) and include the completed checklist as Supporting Information. Please add the following statement, or similar, to the Methods: "This study is reported as per the Transparent Reporting of a Multivariable Prediction Model for Individual Prognosis Or Diagnosis (TRIPOD) statement (S1 Checklist)." For studies using machine learning, please use the TRIPOD-AI checklist. When completing the checklist, please use section and paragraph numbers, rather than page numbers.

DIAGNOSTIC STUDIES

*Please ensure that the study is reported according to the STARD guideline (https://www.equator-network.org/reporting-guidelines/stard/) and include the completed STARD checklist as Supporting Information. Please add the following statement, or similar, to the Methods: "This study is reported as per the Standards for Reporting of Diagnostic Accuracy (STARD) guideline (S1 Checklist)." When completing the checklist, please use section and paragraph numbers, rather than page numbers.

*Please structure your Abstract according to STARD for Abstracts (https://www.equator-network.org/reporting-guidelines/stard-abstracts/).

*Please structure the Methods section using the following sub-headings: Study design, Participants, Test methods, Analysis.

*Please include a diagram to describe the flow of participants through the study (typically figure 1).

MENDELIAN RANDOMIZATION STUDIES

*Please ensure that the study is reported according to the STROBE-MR guideline (https://www.equator-network.org/reporting-guidelines/strobe/) and include the completed STROBE-MR checklist as Supporting Information. Please add the following statement, or similar, to the Methods: "This study is reported as per the Strengthening the Reporting of Observational Studies in Epidemiology (STROBE) guideline, specific for mendelian randomization (S1 Checklist)." When completing the checklist, please use section and paragraph numbers, rather than page numbers.

*In the Introduction, please describe the exposure and the evidence for a potential causal relationship between exposure and outcome.

*In the Methods, please explicitly state the 3 core instrumental variable assumptions for the main analysis (relevance, independence, and exclusion restriction), as well assumptions for any additional or sensitivity analysis.

*In the Methods, please describe the MR estimator (e.g., 2-stage least squares, Wald ratio) and related statistics. Detail the included covariates and, in case of 2-sample MR, whether the same covariate set was used for adjustment in the 2 samples.

*If you are presenting an instrumental variable estimate, please compare this to the conventional observational estimate.

*Report the associations between genetic variant and exposure and between genetic variant and outcome, preferably on an interpretable scale.

*Report MR estimates of the relationship between exposure and outcome and the measures of uncertainty from the MR analysis, on an interpretable scale, such as odds ratio or relative risk per SD difference.

*If relevant, please consider translating estimates of relative risk into absolute risk for a meaningful time period.

*Please consider including plots to visualize results (e.g., forest plot, scatterplot of associations between genetic variants and outcome vs between genetic variants and exposure).

SURVEY-BASED STUDIES

*Please ensure that the study is reported according to the CROSS guideline (https://www.equator-network.org/reporting-guidelines/a-consensus-based-checklist-for-reporting-of-survey-studies-cross/) and include the completed CROSS checklist as Supporting Information. Please add the following statement, or similar, to the Methods: "This study is reported as per A Consensus-Based Checklist for Reporting of Survey Studies (CROSS) guideline (S1 Checklist)." When completing the checklist, please use section and paragraph numbers, rather than page numbers.

*Please report your survey response rates according to AAPOR recommendations (https://aapor.org/standards-and-ethics/best-practices/)

*Please define how the population surveyed was sampled.

*Please compare characteristics of respondents and nonrespondents if possible.

*If sequential waves of the survey were sent, please specify whether the characteristics of respondents changed over time or remained constant.

*Please include the survey response rate in the Abstract.

*Please include a copy of the survey in the supplementary files.

SYSTEMATIC REVIEWS & META-ANALYSES

*Please report your SR/MA according to the PRISMA guidelines provided at the EQUATOR site. http://www.equator-network.org/reporting-guidelines/prisma/. Please provide the completed PRISMA checklist as Supporting Information. When completing the checklist, please use section and paragraph numbers, rather than page numbers. Please add the following statement, or similar, to the Methods: "This study is reported as per the Preferred Reporting Items for Systematic Reviews and Meta-Analyses (PRISMA) guideline (S1 Checklist)."

*Abstract: Please report your abstract according to PRISMA for abstracts (https://doi.org/10.1371/journal.pmed.1001419) following the PLOS Medicine abstract structure (Background, Methods and Findings, Conclusions). Please ensure you provide dates of search, data sources, number of studies included, types of study designs included, eligibility criteria, and synthesis/appraisal methods.

*Please note that we expect searches to be updated to within 6 months of the time of submission.

QUALITATIVE STUDIES

*Please report your qualitative study according to the appropriate study design provided at (http://www.equator-network.org/?post_type=eq_guidelines&eq_guidelines_study_design=qualitative-research&eq_guidelines_clinical_specialty=0&eq_guidelines_report_section=0&s=) and provide the relevant completed checklist as a supplemental file. In the checklist, please include sufficient text excerpted from the manuscript to explain how you accomplished all applicable items. When completing checklists, please use section and paragraph numbers, rather than page numbers.

*We recommend that authors use the COREQ checklist, or other relevant checklists listed by the Equator Network, such as the SRQR, to ensure complete reporting (see: http://www.equator-network.org/?post_type=eq_guidelines&eq_guidelines_study_design=qualitative-research&eq_guidelines_clinical_specialty=0&eq_guidelines_report_section=0&s=). Please add the following statement, or similar, to the Methods: "This study is reported as per the Consolidated criteria for reporting qualitative research (COREQ): a 32-item checklist for interviews and focus groups (S1 Checklist)."

*In general, we expect qualitative studies to include the following: 1) defined objectives or research questions; 2) description of the sampling strategy, including rationale for the recruitment method, participant inclusion/exclusion criteria and the number of participants recruited; 3) detailed reporting of the data collection procedures; 4) data analysis procedures described in sufficient detail to enable replication; 5) a discussion of potential sources of bias; and 6) a discussion of limitations.

HEALTH ECONOMICS / COST-EFFECTIVENESS STUDIES

*Please ensure that the study is reported according to the CHEERS guideline (available from: https://www.equator-network.org/reporting-guidelines/cheers) and include the completed checklist as Supporting Information. Please add the following statement, or similar, to the Methods: "This study is reported as per the Strengthening the Consolidated Health Economic Evaluation Reporting Standards 2022 (CHEERS 2022) Statement (S1 Checklist)." When completing the checklist, please use section and paragraph numbers, rather than page numbers.

# Author comment: Author response to Decision Letter 2

**DOI:** 10.1371/journal.pmed.1004861.r003

18 Nov 2025

Attachment Submitted filename: Response to Reviewers.docx

# Peer review: Decision Letter 2

**DOI:** 10.1371/journal.pmed.1004861.r004

**Authors:** Alison Farrell, Alison Farrell, Alison Farrell

9 Jan 2026

Dear Dr Martin,

Many thanks for submitting your manuscript "THE GROWING DISCORDANCE IN ORPHAN DRUG APPROVALS BETWEEN THE FDA AND THE EMA: ASSOCIATIONS WITH THERAPEUTIC AREA, COMPANY SIZE, AND HEADQUARTERS LOCATION" (PMEDICINE-D-24-04261R2) to PLOS Medicine. The paper has been reviewed by subject experts and a statistician; their comments are included below and can also be accessed here: [LINK]

As you will see, the reviewers find the manuscript is improved but continue to raise important concerns that warrant addressing. After discussing the paper with the editorial team and an academic editor with relevant expertise, I'm pleased to invite you to revise the paper in response to all the reviewers' comments. We plan to send the revised paper to some or all of the original reviewers, and we cannot provide any guarantees at this stage regarding publication.

In addition to these revisions, you may need to complete some formatting changes, which you will receive in a follow up email. A member of our team will be in touch with a set of requests shortly. If you do not receive a separate email within a few days, please assume that checks have been completed, and no additional changes are required.

When you upload your revision, please include a point-by-point response that addresses all of the reviewer and editorial points, indicating the changes made in the manuscript and either an excerpt of the revised text or the location (eg: page and line number) where each change can be found. Please also be sure to check the general editorial comments at the end of this letter and include these in your point-by-point response. When you resubmit your paper, please include a clean version of the paper as the main article file and a version with changes tracked as a marked-up manuscript. It may also be helpful to check the guidelines for revised papers at http://journals.plos.org/plosmedicine/s/revising-your-manuscript for any that apply to your paper.

We ask that you submit your revision by Jan 30 2026 11:59PM. However, if this deadline is not feasible, please contact me by email, and we can discuss a suitable alternative.

Don't hesitate to contact me directly with any questions (afarrell@plos.org).

Best regards,

Alison

Alison Farrell, Ph.D.

Senior Editor

PLOS Medicine

afarrell@plos.org

-----------------------------------------------------------

Comments from the academic editor:

The manuscript is of interest to a broader audience beyond the EU and the US, as other regulatory agencies have had to make similar decisions regarding orphan drugs.

As PLOS Medicine is a medical journal, it would be valuable to see more evidence on the public health impact of these decisions in non-covered diseases.

-----------------------------------------------------------

Comments from the reviewers:

Reviewer #1: Major concerns

1. I would like to thank the authors for the considerable work on improving the description of the methodology sections. This is much improved and clearer. However, I have major concerns about the over-interpretation of the research based on the data derived from this methodology. Examples include claims made about therapeutic area when in reality is only whether a medicines falls under a cancer or non-cancer category - this is insufficiently granular to claim therapeutic assessment considering the breadth of rare diseases. There are also conclusions and statements - for example 'most are not designated as orphan products due to regulatory differences regarding significant benefit and sub-setting of diseases based on biomarkers, particularly in the field of oncology' that are unsubstantiated. Although it is agreed significant benefit and subsetting are main drivers for the difference, this does not come from the current research or linked clearly to the published literature. The authors are advised to ensure that any claims made based on their data are clearly defined, where other conclusions or claims are made these should be sufficiently supported by the published literature and well justified.

Other issues include:

2. Orphan approvals terminology within the text remains difficult to follow - this needs to be clearer for the reader. Table 1 is helpful but could better define to EMA description and FDA status

3. Pembrolizumab example should discuss whether not having orphan and non-orphan indications on the same MA is a barrier in the EU - there will be a commercial decision here

4. New errors are introduced - e.g. This shift is exemplified by the FDA's 2017 tissue-agnostic approval of pembrolizumab for solid tumours with NTRK-fusion proteins - this was not pembrolizumab - please carefully check your text for errors

5. Limitations - Based on the research goal to understand the discordance between US and EU, a major limitation of this study is not addressing from a research perspective the differences in regulatory ask on significant benefit in the EU, and other regulatory criteria e.g. allowance of subsetting or stage of condition from US perspective which is acceptable for FDA status.

6. Duplication of a reference is noted - 29 and 33

Reviewer #2: Thank you for the thoughtful revisions. They have strengthened the manuscript, while I have just one remaining methodological point.

I agree that many regulatory or policy analyses do not routinely report pseudo-R² or other global fit measures. But some recent work has included such metrics simply to illustrate how much of the variation is explained by the available covariates. For example, Hong et al. (Hong, D., Kesselheim, A.S., Sarpatwari, A. and Rome, B.N., 2024. Biosimilar Uptake In The US: Patient And Prescriber Factors: Article examines biosimilar uptake in the US. Health Affairs, 43(8), pp.1159-1164.) reported the McFadden pseudo-R² to help contextualize the limitations of the model, and they also discussed the contribution of unmeasured factors.

I understand that your analysis does not involve model selection or causal inference. My suggestion is purely about transparency: providing a brief pseudo-R² value in the Supplementary Appendix or notes below the table would help readers understand how much of the EMA decision variation the prespecified variables account for, given that several regulatory/pricing/political framework determinants cannot be directly measured.

Overall, the revisions are strong, and addressing this small point would further clarify the completeness and interpretability of the adjusted associations.

Reviewer #3: Thanks for the opportunity to read your manuscript. My role is statistical reviewer, so I have focused on the design, data, and analysis that are presented. I have put general comments first, followed by questions relevant to a specific section of the manuscript (with a page/line reference).

I agree with the authors about the use of odds ratios over other measures (e.g. pseudo R2 and AIC/BIC). Pseudo R2 is more useful as measure of discrimination of a particular model, rather than an estimate of how a covariate is associated with an outcome. Measures of model fit (AIC/BIC) also are useful to select between models with different sets of covariates but can't directly quantify the strength of association. The odds ratio is an appropriate effect estimate for this data.

There is some material in the results that belongs in the methods, i.e. the link between an analysis and a particular research question (e.g. L303).

The data for the study is likely to be non-independent, i.e. clustered at the level of the company. Can you confirm that there is no clustering, or else use a method that appropriately accounts for the clustering (e.g. GEE)?

P7, L221. I wasn't clear about the need for a multivariable logistic regression model. If the analysis is causal, and includes covariates that are potential confounders or mediators, then the proposed causal model needs to be explained to ensure that effects from the model are interpreted correction (i.e. 'table 2 fallacy' https://doi.org/10.1093/aje/kws412). Measures of discrimination (AUC, sens/spec) are presented, but the research question does not seem to be to build a prediction model for the different authorisation/approval outcomes. For a descriptive or causal analysis, measures of discrimination are not particularly useful as they simple quantify the overall level of predictions (concordance) rather than assessing the quality of the model. If the aim is predictive, an assessment of calibration should also be included. If the aim is more descriptive, then multivariable models may not be necessary and a series of univariate regression models for each outcome and covariate would be more appropriate.

P10, L314. It would be clearer to just report the odds ratio rather than the % higher or lower the odds are.

Figure S6. This is interesting data - but it is difficult to understand a 3d bar plot. This could be better displayed a time-series plot, with each therapeutic area as a series, or as a panel plot of the time-series with an individual section for each therapeutic area.

Any attachments provided with reviews can be seen via the following link: [LINK]

--------------------------------------------------------- ---

General editorial requests:

(Note: not all will apply to your paper, but please check each item carefully)

*We ask every co-author listed on the manuscript to fill in a contributing author statement, making sure to declare all competing interests. If any of the co-authors have not filled in the statement, we will remind them to do so when the paper is revised. If all statements are not completed in a timely fashion this could hold up the re-review process. If new competing interests are declared later in the revision process, this may also hold up the submission. Should there be a problem getting one of your co-authors to fill in a statement we will be in contact. Please do not add or remove authors without first discussing this with the handling editor. You can see our competing interests policy here: http://journals.plos.org/plosmedicine/s/competing-interests.

*Please upload any figures associated with your paper as individual TIF or EPS files with 300dpi resolution at resubmission; please read our figure guidelines for more information on our requirements: http://journals.plos.org/plosmedicine/s/figures. While revising your submission, we strongly recommend that you use PLOS's NAAS tool (https://ngplosjournals.pagemajik.ai/artanalysis) to test your figure files. NAAS can convert your figure files to the TIFF file type and meet basic requirements (such as print size, resolution), or provide you with a report on issues that do not meet our requirements and that NAAS cannot fix.

After uploading your figures to PLOS's NAAS tool - https://ngplosjournals.pagemajik.ai/artanalysis, NAAS will process the files provided and display the results in the "Uploaded Files" section of the page as the processing is complete.

If the uploaded figures meet our requirements (or NAAS is able to fix the files to meet our requirements), the figure will be marked as "fixed" above. If NAAS is unable to fix the files, a red "failed" label will appear above.

When NAAS has confirmed that the figure files meet our requirements, please download the file via the download option, and include these NAAS processed figure files when submitting your revised manuscript.

*Please ensure that the paper adheres to the PLOS Data Availability Policy (see http://journals.plos.org/plosmedicine/s/data-availability), which requires that all data underlying the study's findings be provided in a repository or as Supporting Information. For data residing with a third party, authors are required to provide instructions with contact information (web or email address) for obtaining the data. Please note that a study author cannot be the contact person for the data. PLOS journals do not allow statements supported by "data not shown" or "unpublished results." For such statements, authors must provide supporting data or cite public sources that include it.

*We expect all researchers with submissions to PLOS in which author-generated code underpins the findings in the manuscript to make all author-generated code available without restrictions upon publication of the work. In cases where code is central to the manuscript, we may require the code to be made available as a condition of publication. Authors are responsible for ensuring that the code is reusable and well documented. Please make any custom code available, either as part of your data deposition or as a supplementary file. Please add a sentence to your data availability statement regarding any code used in the study, e.g. "The code used in the analysis is available from Github [URL] and archived in Zenodo [DOI link]" Please review our guidelines at https://journals.plos.org/plosmedicine/s/materials-software-and-code-sharing and ensure that your code is shared in a way that follows best practice and facilitates reproducibility and reuse. Because Github depositions can be readily changed or deleted, we encourage you to make a permanent DOI'd copy (e.g. in Zenodo) and provide the URL.

*Please ensure that the study is reported according to the STOBE guideline and include the completed checklist as Supporting Information. When completing the checklist, please use section and paragraph numbers, rather than page numbers. Please add the following statement, or similar, to the Methods: "This study is reported as per [XXXX] guideline (S1 Checklist)."

FORMATTING - GENERAL

* Please use the active voice throughout.

* Please be consistent in use of verb tenses.

* Please be concise in presentation of the Results.

* The Discussion is >2500 words. Please reduce to 1000 words.

* Please retain the discussion of the potential health impacts of differences in drug approvals, and whether data are available on this issue.

*Abstract: Please structure your abstract using the PLOS Medicine headings (Background, Methods and Findings, Conclusions). Please combine the Methods and Findings sections into one section.

* In the last sentence of the Abstract Methods and Findings section, please describe the main limitation(s) of the study's methodology.

* Please limit the Conclusions section of the Abstract to 4 sentences pertaining to the findings and their implications. This should not reiterate background information.

*At this stage, we ask that you include a short, non-technical Author Summary of your research to make findings accessible to a wide audience that includes both scientists and non-scientists. The Author Summary should immediately follow the Abstract in your revised manuscript. This text is subject to editorial change and should be distinct from the scientific abstract. Ideally each sub-heading should contain 2-3 single sentence, concise bullet points containing the most salient points from your study. In the final bullet point of 'What Do These Findings Mean?', please include the main limitations of the study in non-technical language. Please see our author guidelines for more information: https://journals.plos.org/plosmedicine/s/revising-your-manuscript#loc-author-summary.

*Please express the main results with 95% CIs as well as p values. When reporting p values please report as p<0.001 and where higher as the exact p value p=0.002, for example. Throughout, suggest reporting statistical information as follows to improve clarity for the reader "22% (95% CI [13%,28%]; p</=)". Please be sure to define all numerical values at first use.

*Please include page numbers and line numbers in the manuscript file. Use continuous line numbers (do not restart the numbering on each page).

*Please cite the reference numbers in square brackets. Citations should precede punctuation.

* Please revise your title to comply with PLOS Medicine's style. Your title must be nondeclarative and not a question. It should begin with main concept if possible. "Effect of" should be used only if causality can be inferred, i.e., for an RCT. Please place the study design ("A randomized controlled trial," "A retrospective study," "A modelling study," etc.) in the subtitle (ie, after a colon).

* Please ensure that the Introduction ends with a clear description of the study question or hypothesis.

* Please ensure that all abbreviations are defined at first use throughout the text.

* Please confirm that all numbers presented in the abstract are present and identical to numbers presented in the main manuscript text.

* Please remove all subheadings from the Discussion.

* Please add URLs for funders.

FIGURES AND TABLES

*Please provide titles and legends for all figures and tables (including those in Supporting Information files).

*Please define all abbreviations used in each figure/table (including those in Supporting Information files).

*Please consider avoiding the use of red and green in order to make your figure more accessible to those with color blindness.

SUPPLEMENTARY MATERIAL

*Please note that supplementary material will be posted as supplied by the authors. Therefore, please amend it according to the relevant comments outlined here.

*Please cite your Supporting Information as outlined here: https://journals.plos.org/plosmedicine/s/supporting-information

REFERENCES

*PLOS uses the numbered citation (citation-sequence) method and first six authors, et al.

*Please ensure that journal name abbreviations match those found in the National Center for Biotechnology Information (NCBI) databases (http://www.ncbi.nlm.nih.gov/nlmcatalog/journals), and are appropriately formatted and capitalised.

*Where website addresses are cited, please include the complete URL and specify the date of access (e.g. [accessed: 12/06/2023]).

*Please also see https://journals.plos.org/plosmedicine/s/submission-guidelines#loc-references for further details on reference formatting.

OBSERVATIONAL STUDIES

*Abstract: Please include the study design, population and setting, number of participants, years during which the study took place (enrollment and follow up), length of follow up, and main outcome measures.

*Please ensure that the study is reported according to the STROBE (or appropriate STOBE extension) guideline (available from: https://www.equator-network.org/reporting-guidelines/strobe) and include the completed STROBE (or STROBE extension) checklist as Supporting Information. Please add the following statement, or similar, to the Methods: "This study is reported as per the Strengthening the Reporting of Observational Studies in Epidemiology (STROBE) guideline (S1 Checklist)." When completing the checklist, please use section and paragraph numbers, rather than page numbers.

*For all observational studies, in the manuscript text, please indicate: (1) the specific hypotheses you intended to test, (2) the analytical methods by which you planned to test them, (3) the analyses you actually performed, and (4) when reported analyses differ from those that were planned, transparent explanations for differences that affect the reliability of the study's results. If a reported analysis was performed based on an interesting but unanticipated pattern in the data, please be clear that the analysis was data driven.

*Please state in the Methods section whether the study had a prospective protocol or analysis plan. If a prospective analysis plan (from your funding proposal, IRB or other ethics committee submission, study protocol, or other planning document written before analyzing the data) was used in designing the study, please include the relevant document(s) with your revised manuscript as a Supporting Information file to be published alongside your study and cite it in the Methods section. A legend for this file should be included at the end of your manuscript. If no such document exists, please make sure that the Methods section transparently describes when analyses were planned, and when/why any data-driven changes to analyses took place. Changes in the analysis, including those made in response to peer review comments, should be identified as such in the Methods section of the paper, with rationale.

# Author comment: Author response to Decision Letter 3

**DOI:** 10.1371/journal.pmed.1004861.r005

6 Feb 2026

Attachment Submitted filename: Reponse to comments_v2.2.docx

# Peer review: Decision Letter 3

**DOI:** 10.1371/journal.pmed.1004861.r006

**Authors:** Alison Farrell, Alison Farrell, Alison Farrell, Alison Farrell

20 May 2026

Dear Dr. Martin,

Thank you very much for re-submitting your manuscript "THE GROWING DISCORDANCE IN ORPHAN DRUG APPROVALS BETWEEN THE FDA AND THE EMA: ASSOCIATIONS WITH THERAPEUTIC AREA, COMPANY SIZE, AND HEADQUARTERS LOCATION" (PMEDICINE-D-24-04261R3) for review by PLOS Medicine.

I have discussed the paper with my colleagues and the academic editor and it was also seen again by two reviewers. I am pleased to say that provided the remaining editorial and production issues are dealt with we are planning to accept the paper for publication in the journal.

The remaining issues that need to be addressed are listed at the end of this email and in the attached file. Any accompanying reviewer attachments can be seen via the link below. Please take these into account before resubmitting your manuscript and provide a point-by-point rebuttal to the requests in this letter:

[LINK]

***Please note while forming your response, if your article is accepted, you may have the opportunity to make the peer review history publicly available. The record will include editor decision letters (with reviews) and your responses to reviewer comments. If eligible, we will contact you to opt in or out.***

In revising the manuscript for further consideration here, please ensure you address the specific points made by each reviewer and the editors. In your rebuttal letter you should indicate your response to the reviewers' and editors' comments and the changes you have made in the manuscript. Please submit a clean version of the paper as the main article file. A version with changes marked must also be uploaded as a marked up manuscript file.

Please also check the guidelines for revised papers at http://journals.plos.org/plosmedicine/s/revising-your-manuscript for any that apply to your paper. If you haven't already, we ask that you provide a short, non-technical Author Summary of your research to make findings accessible to a wide audience that includes both scientists and non-scientists. The Author Summary should immediately follow the Abstract in your revised manuscript. This text is subject to editorial change and should be distinct from the scientific abstract.

In addition to these revisions, you may need to complete some formatting changes, which you will receive in a follow up email. A member of our team will be in touch with a set of requests shortly. If you do not receive a separate email within a few days, please assume that checks have been completed, and no additional changes are required.

We expect to receive your revised manuscript within 1 week. Please email us (plosmedicine@plos.org) if you have any questions or concerns.

We ask every co-author listed on the manuscript to fill in a contributing author statement. If any of the co-authors have not filled in the statement, we will remind them to do so when the paper is revised. If all statements are not completed in a timely fashion this could hold up the re-review process. Should there be a problem getting one of your co-authors to fill in a statement we will be in contact. YOU MUST NOT ADD OR REMOVE AUTHORS UNLESS YOU HAVE ALERTED THE EDITOR HANDLING THE MANUSCRIPT TO THE CHANGE AND THEY SPECIFICALLY HAVE AGREED TO IT.

Please ensure that the paper adheres to the PLOS Data Availability Policy (see http://journals.plos.org/plosmedicine/s/data-availability), which requires that all data underlying the study's findings be provided in a repository or as Supporting Information. For data residing with a third party, authors are required to provide instructions with contact information for obtaining the data. PLOS journals do not allow statements supported by "data not shown" or "unpublished results." For such statements, authors must provide supporting data or cite public sources that include it.

To enhance the reproducibility of your results, we recommend that you deposit your laboratory protocols in protocols.io, where a protocol can be assigned its own identifier (DOI) such that it can be cited independently in the future. Additionally, PLOS ONE offers an option to publish peer-reviewed clinical study protocols. Read more information on sharing protocols at https://plos.org/protocols?utm_medium=editorial-email&utm_source=authorletters&utm_campaign=protocols

Please review your reference list to ensure that it is complete and correct. If you have cited papers that have been retracted, please include the rationale for doing so in the manuscript text, or remove these references and replace them with relevant current references. Any changes to the reference list should be mentioned in the rebuttal letter that accompanies your revised manuscript.

Please note, when your manuscript is accepted, an uncorrected proof of your manuscript will be published online ahead of the final version, unless you've already opted out via the online submission form. If, for any reason, you do not want an earlier version of your manuscript published online or are unsure if you have already indicated as such, please let the journal staff know immediately at plosmedicine@plos.org.

If you have any questions in the meantime, please contact me or the journal staff on plosmedicine@plos.org.

We look forward to receiving the revised manuscript by May 27 2026 11:59PM.

Sincerely,

Alison Farrell, Ph.D.

Senior Editor

PLOS Medicine

plosmedicine.org

------------------------------------------------------------

Requests from Editors:

* In the final bullet point of the Author Summary, in ‘What Do These Findings Mean?’ Please include the main limitations of the study in non-technical language.

Please see our author guidelines for more information: https://journals.plos.org/plosmedicine/s/revising-your-manuscript#loc-author-summary.

* Please confirm that your title complies with PLOS Medicine's style. Your title must be nondeclarative and not a question. It should begin with main concept if possible. "Effect of" should be used only if causality can be inferred, i.e., for an RCT. Please place the study design ("A randomized controlled trial," "A retrospective study," "A modelling study," etc.) in the subtitle (ie, after a colon). Please see the suggested revised title.

* Please confirm that your abstract complies with our requirements, including format (three sections: Background, Methods and Findings, and Conclusions) and providing all the information relevant to this study type https://journals.plos.org/plosmedicine/s/submission-guidelines#loc-abstract

* Please ensure that the Introduction ends with a clear description of the study question or hypothesis.

* Please ensure that all abbreviations are defined at first use throughout the text (e.g. HTA).

* Please confirm that all numbers presented in the abstract are present and identical to numbers presented in the main manuscript text.

GENERAL

* Please disclose any use of AI tools and technologies to the study or article content in the Methods, including the name(s) of any tool(s) used and a description of their use. Please see our guidelines for more information: https://journals.plos.org/plosmedicine/s/ethical-publishing-practice#loc-artificial-intelligence-tools-and-technologies.

* Please review your text for claims of novelty or primacy (e.g. 'for the first time') and remove this language. In addition, please check that any use of statistical terms (such as trend or significant) are supported by the data, and if not please remove them.

* Statistical reporting: Please revise throughout the manuscript, including tables and figures.

- Please report statistical information as follows to improve clarity for the reader "22% (95% CI [13,28]; p</=)".

- Please separate upper and lower bounds with commas instead of hyphens as the latter can be confused with reporting of negative values.

- Please repeat statistical definitions (HR, CI etc.) for each set of parentheses.

* In the abstract, please include the important dependent variables that are adjusted for in the analyses.

* In the author summary, in the final bullet point of 'What Do These Findings Mean?', please include the main limitations of the study in non-technical language.

* Please remove all causal language from the study and use associative language.

FUNDING STATEMENT

* The funding statement should include: specific grant numbers, initials of authors who received each award, URLs to sponsors’ websites. Also, please state whether any sponsors or funders (other than the named authors) played any role in study design, data collection and analysis, the decision to publish, or preparation of the manuscript. If they had no role in the research, include this sentence: “The funders had no role in study design, data collection and analysis, decision to publish, or preparation of the manuscript.” A URL for NovoNordisk is missing.

COMPETING INTERESTS STATEMENT

* All authors must declare their relevant competing interests per the PLOS policy, which can be seen here: https://journals.plos.org/plosmedicine/s/competing-interests For authors with ties to industry, please indicate whether any of the interests has a financial stake in the results of the current study.

*Please amend your competing interests statement. Funding information should go to the relevant section and include URLs.

DATA AVAILABILITY

* PLOS defines the “minimal data set” to consist of the data set used to reach the conclusions drawn in the manuscript with related metadata and methods, and any additional data required to replicate the reported study findings in their entirety. Authors do not need to submit their entire data set, or the raw data collected during an investigation. Please submit the following data:

The values behind the means, standard deviations and other measures reported;

The values used to build graphs;

The points extracted from images for analysis.

* Thank you for agreeing to make your data available. At this time, please provide the link to the data repository and accession numbers required for access.

* If new code was used to generate results, please deposit the code in a public repository and include the statement on code availability in the data availability statement. Please see guidance here:https://journals.plos.org/plosmedicine/s/code-sharing-guidance#loc-data-availability-statement-examples

Tables and Figures

* When a p value is given, please specify the statistical test used to determine it in the legend.

* Please specify the variables controlled for in all relevant Tables

OBSERVATIONAL, COHORT, CROSS-SECTIONAL, AND CASE CONTROL STUDIES

* Please ensure that the study is reported according to the STROBE guideline, and include the completed STROBE checklist as Supporting Information. Please add the following statement, or similar, to the Methods: "This study is reported as per the Strengthening the Reporting of Observational Studies in Epidemiology (STROBE) guideline (S1 Checklist)."

The STROBE guideline can be found here: http://www.equator-network.org/reporting-guidelines/strobe/

When completing the checklist, please use section and paragraph numbers, rather than page numbers.

Comments from Reviewers:

Reviewer #2: The authors have already addressed my comments.

Reviewer #3: Thanks for the revised manuscript and responses to my original review.

The revised manuscript and responses to my questions have resolved my original questions.

Any attachments provided with reviews can be seen via the following link:

[LINK]

Attachment Submitted filename: Martin_2026.docx

# Author comment: Author response to Decision Letter 4

**DOI:** 10.1371/journal.pmed.1004861.r007

4 Jun 2026

Attachment Submitted filename: Responses to editors_6.2.2026.docx

# Editor report: Decision Letter 4

**DOI:** 10.1371/journal.pmed.1004861.r008

**Authors:** Alison Farrell, Alison Farrell, Alison Farrell, Alison Farrell, Alison Farrell

5 Jun 2026

Dear Dr Martin,

On behalf of my colleagues and the Academic Editor, Jorge Pacheco, I am pleased to inform you that we have agreed to publish your manuscript "Discordance in Orphan Drug Approvals between the U.S. Food and Drug Administration and the European Medicines Agency: A Retrospective Observational Analysis" (PMEDICINE-D-24-04261R4) in PLOS Medicine.

Before your manuscript can be formally accepted you will need to complete some formatting changes, which you will receive in a follow up email. Please be aware that it may take several days for you to receive this email; during this time no action is required by you. Once you have received these formatting requests, please note that your manuscript will not be scheduled for publication until you have made the required changes.

Please also make the follow minor edits. Please note the requirement for a statement regarding methodological limitations in the Abstract:

line 40: delete " EMA"

line 56: replace 'appear to' with 'may'

line 77: delete "is"

line 78-79: add potentially to read "potentially for commercial reasons"

line 144: insert space between approval and while

line 150: replace 'search' with 'searched'

line 281: replace 'largely' with 'perhaps'

line 401: replace "appears to' with 'may'

* In the last sentence of the Abstract Methods and Findings section, please describe the main limitation(s) of the study's methodology.

Please temper speculation throughout regarding company decisions in the absence of direct evidence, as noted above.

In the meantime, please log into Editorial Manager at http://www.editorialmanager.com/pmedicine/, click the "Update My Information" link at the top of the page, and update your user information to ensure an efficient production process.

PRESS

We frequently collaborate with press offices. If your institution or institutions have a press office, please notify them about your upcoming paper at this point, to enable them to help maximise its impact. If the press office is planning to promote your findings, we would be grateful if they could coordinate with medicinepress@plos.org. If you have not yet opted out of the early version process, we ask that you notify us immediately of any press plans so that we may do so on your behalf.

We also ask that you take this opportunity to read our Embargo Policy regarding the discussion, promotion and media coverage of work that is yet to be published by PLOS. As your manuscript is not yet published, it is bound by the conditions of our Embargo Policy. Please be aware that this policy is in place both to ensure that any press coverage of your article is fully substantiated and to provide a direct link between such coverage and the published work. For full details of our Embargo Policy, please visit http://www.plos.org/about/media-inquiries/embargo-policy/.

To enhance the reproducibility of your results, we recommend that you deposit your laboratory protocols in protocols.io, where a protocol can be assigned its own identifier (DOI) such that it can be cited independently in the future. Additionally, PLOS ONE offers an option to publish peer-reviewed clinical study protocols. Read more information on sharing protocols at https://plos.org/protocols?utm_medium=editorial-email&utm_source=authorletters&utm_campaign=protocols

Thank you again for submitting to PLOS Medicine. We look forward to publishing your paper.

Sincerely,

Alison Farrell, Ph.D.

Senior Editor

PLOS Medicine
